# Discovery of cytotoxic indolo[1,2-*c*]quinazoline derivatives through scaffold-based design

**DOI:** 10.3762/bjoc.21.161

**Published:** 2025-10-13

**Authors:** Daniil Viktorovich Khabarov, Valeria Alexandrovna Litvinova, Lyubov Georgievna Dezhenkova, Dmitry Nikolaevich Kaluzhny, Alexander S Tikhomirov, Andrey Egorovich Shchekotikhin

**Affiliations:** 1 Gause Institute of New Antibiotics, 11 B. Pirogovskaya Street, Moscow 119021, Russia; 2 Engelhardt Institute of Molecular Biology, Russian Academy of Sciences, 32 Vavilov Street, Moscow 11991, Russia

**Keywords:** antiproliferative activity, antitumor agents, indolo[1,2-*c*]quinazoline, modification, structure–activity relationship

## Abstract

Indolo[1,2-*c*]quinazoline derivatives have emerged as promising chemotype in drug discovery due to their versatile biological activities, including antimicrobial and antiviral properties. In this study, we report the design, synthesis, and biological evaluation of novel indolo[1,2-*c*]quinazoline derivatives, with a particular focus on their antiproliferative potential against human cancer cells. We introduced structural modifications at positions 5, 6, and 12 of the indolo[1,2-*c*]quinazoline core to explore the structure–activity relationships and enhance cytotoxicity. Our results highlight that 12-aminomethyl derivatives exhibited notable cytotoxicity against tumor cell lines, with the highest activity observed for compound **9c**, which showed significant selectivity toward tumor cells. In contrast, while the compounds demonstrated planar polycyclic structures, DNA was not the primary target for their antiproliferative effects, as confirmed by FID assay and fluorescence titration studies. This study represents the first comprehensive evaluation of indolo[1,2-*c*]quinazolines as potential scaffold for the development of antitumor agents, offering valuable insights into their SAR and paving the way for a future evaluation of these compounds as anticancer therapeutics.

## Introduction

Organic compounds featuring heterocyclic scaffolds are widely used in the treatment of various diseases, making them a common focus of research [1]. A substantial part of these compounds incorporate five- or six-membered nitrogen heterocycles. The indole and quinazoline cores represent two pharmacologically significant heterocyclic systems, exhibiting a wide range of biological activity, high level of druglikeness and broad opportunities for derivatizations, that make them privileged scaffolds in drug discovery [2–3]. The pharmaceutical significance of indole and quinazoline rings is evident by numerous FDA-approved drugs across diverse therapeutic areas, including oncology (gefitinib, erlotinib), antiviral therapy (delavirdine, umifenovirum), CNS disorders (sertindole), and other directions (tadalafil) [4–5].

The annelation of quinazoline with nitrogen-containing heterocycles at the N(3)–C(4) bond represents a strategically important modification for generating novel bioactive compounds with enhanced pharmacological potential [6–7]. A prominent example is copanlisib, a 2,3-dihydroimidazo[1,2-*c*]quinazoline derivative approved for relapsed follicular lymphoma, which, however, was later withdrawn by Bayer in 2023 [8]. The structural fusion of indole and quinazoline pharmacophores offers exceptional opportunities for designing new therapeutic agents, combining the proven bioactivities of both privileged scaffolds.

**Figure 1:**
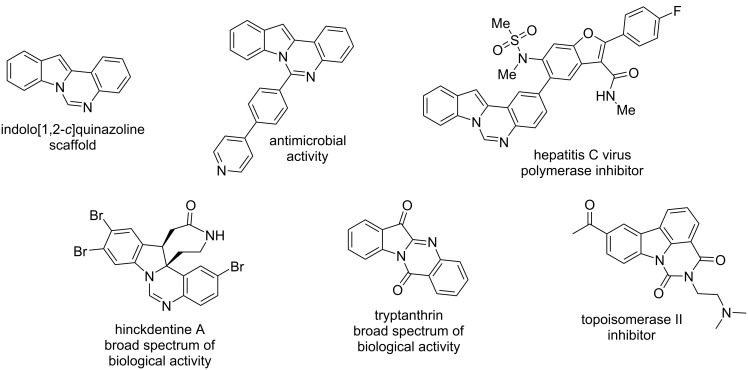
Structure of indolo[1,2-*c*]quinazoline, its selected derivatives, and related structures with biological activity.

Chemical ways for the synthesis of indolo[1,2-*c*]quinazolines (Figure 1, top) are numerous and cover different methodologies from traditional acylation/carbamoylation [9] to advanced Pd- or Rh-catalyzed C–H activation [10–11], Fe^III^–Cu^II^/*p*-TSA–Cu^I^ catalyzed ring expansion/cyclization [12], electrochemical C–H/N–H functionalization [13], Rh^III^-catalyzed C–H amidation [14], etc. In contrast to chemical studies, a systematic analysis of biological properties of indolo[1,2-*c*]quinazoline derivatives remains to be unexplored. Up to date, only limited results were presented. Rohini et al. have revealed antimicrobial potential in 6-substituted indolo[1,2-*c*]quinazoline derivatives (Figure 1, top) [15–16]. A series of indolo[1,2-*c*]quinazoline derivatives was patented as anti-HCV compounds acting through selective inhibition of viral polymerase (Figure 1, top) [17]. Other notable bioactive indoloquinazoline compounds include the natural alkaloids tryptanthrin and hinckdentine A (Figure 1, bottom). Tryptanthrin (indolo[2,1-*b*]quinazoline-6,12-dione) and its derivatives are particularly noteworthy as they demonstrate multiple biological activities, including antibacterial, antitumor, antifungal, antiviral, anti-inflammatory, antileishmanial, antiplasmodial, etc. [18–19]. The structurally isomeric class – pyrimido[5,6,1-*jk*]carbazoles possessed exceptionally high in vitro and in vivo antitumor potencies though topoisomerase II inhibition (Figure 1, bottom) [20].

Taken together, an emerging evidence points to the remarkable pharmacological versatility of indolo[1,2-c]quinazoline derivatives, highlighting the need for more extensive structure–activity relationship investigations. This work represents the first systematic evaluation of the anticancer potential of indolo[1,2-*c*]quinazoline derivatives, a chemotype previously studied mainly for synthetic accessibility but not for biological activity. Unlike prior reports that focused on natural alkaloids or limited antimicrobial studies, this study expands the pharmacological scope by demonstrating selective antiproliferative effects and identifying promising SAR trends. The originality of the research lies in linking strategic scaffold functionalization (positions 5, 6, and 12) with anticancer activity, thereby establishing indolo[1,2-*c*]quinazolines as a novel and underexplored platform for drug discovery.

## Results and Discussion

Planar polycyclic compounds including classical frameworks such as acridine, anthraquinone, naphthalenediimide, etc. demonstrate exceptional potential as ligands targeting secondary structures of nucleic acids, particularly G-quadruplexes (G4) [21–22]. The indolo[1,2-*c*]quinazolin-6(5*H*)-one scaffold **1** exemplifies this design principle, with its rigid polycyclic framework mimicking topologies of established DNA/RNA-interactive molecules. Such compounds can intercalate into nucleic acids, primarily through π–π stacking interactions with nitrogenous bases. The introduction of side chains with terminal amino groups enhances binding to oligonucleotides via additional ionic interactions and hydrogen bonds.

**Scheme 1:**
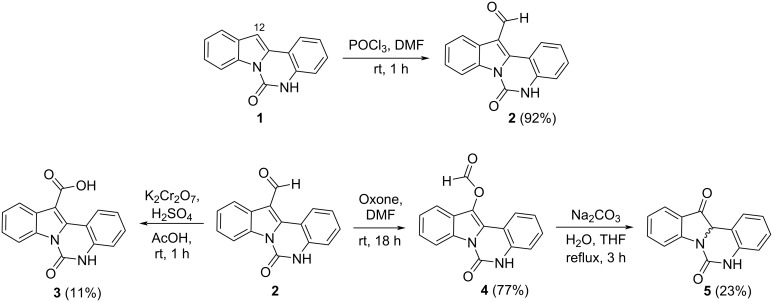
Synthesis of 12-modified indolo[1,2-*c*]quinazoline derivatives.

The indolo[1,2-*c*]quinazolin-6(5*H*)-one scaffold **1** was synthesized according to the optimized protocol developed by Bergman et al. [9]. Position 12 of indolo[1,2-*c*]quinazolin-6(5*H*)-one (**1**) (Scheme 1) corresponded to of the indole C3 position, which is typically used as a nucleophilic center for functionalization via reactions with electrophilic reagents. This site offers a strategic handle for introducing diverse substituents to modulate the compound's properties. Although the indolo[1,2-*c*]quinazolin-6(5*H*)-one scaffold offers considerable potential for structural diversification, current literature describes only the arylation at position 12 [23].

The most straightforward synthetic strategy for formation of an carboxamide group at position 12 of the indolo[1,2-*c*]quinazolin-6(5*H*)-one (**1**) scaffold involved a two-step sequence: (1) carboxylation of the nucleophilic C12 position, followed by coupling with appropriate amines. To introduce the carboxylic acid group a sequence of formylation/oxidation reactions was used. Vilsmeier–Haack reaction of **1** afforded 6-oxoindolo[1,2-*c*]quinazoline-12-carbaldehyde (**2**) (Scheme 1). All attempts to oxidize the aldehyde group of **2** to the corresponding carboxylic acid were hampered by the oxidative sensitivity of the indole moiety, resulting in poor selectivity and formation of complex product mixtures. In particular, Jones oxidation of **2** gave the corresponding 6-oxoindolo[1,2-*c*]quinazoline-12-carboxylic acid (**3**) in a low yield (Scheme 1) making it necessary to look another synthetic pathway.

Of interest, compound **2** applied as a useful substrate for a Baeyer–Villiger oxidation mediated by oxone, which selectively converted the aldehyde to the formate ester, yielding 6-oxo-5,6-dihydroindolo[1,2-*c*]quinazolin-12-yl formate (**4**). Subsequent hydrolysis of **4** furnished indolo[1,2-*c*]quinazoline-6,12-dione (**5**) (Scheme 1), a structural analogue of the biologically active alkaloid tryptanthrin (Figure 1).

**Scheme 2:**
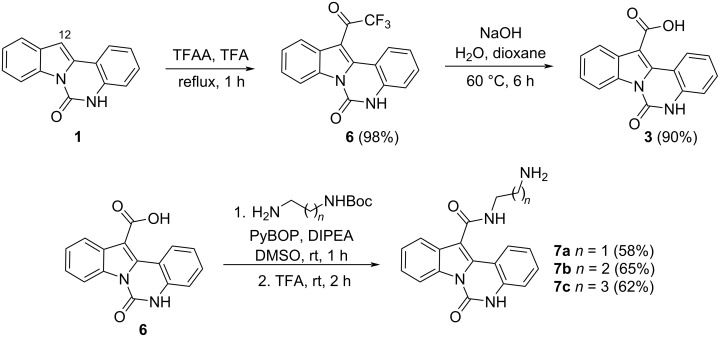
Synthesis of indolo[1,2-*c*]quinazoline-12-carboxamides **7a**–**c**.

An alternative scheme to indolo[1,2-*c*]quinazoline-12-carboxylic acid (**3**) was based on initial acylation followed by a haloform reaction. Refluxing compound **1** with trifluoroacetic anhydride (TFAA) in trifluoroacetic acid affords intermediate compound **6**, bearing a trifluoroacetyl group on the indole moiety. Treatment of **6** with the base yielded the acid **3** in high yield (Scheme 2). The carboxyl group of **3** was converted to the corresponding amides via coupling with mono-*N*-Boc-protected C_2_–C_4_ diamines using PyBOP as the activating agent under standard peptide coupling conditions. Cleavage of the Boc-protecting group with TFA afforded the target 6-oxoindolo[1,2-*c*]quinazoline-12-carboxamides **7a–c** (Scheme 2).

3-Aminomethylindole derivatives represent a well-established class of compounds with diverse biological activities, including antiviral, antibacterial, anti-inflammatory, antitumor, and insecticidal properties. Notably, gramine [24] and other Mannich bases [25] exhibit a broad range of bioactivity, and structurally related compounds such as sumatriptan and rizatriptan have been approved for clinical use [24]. Given the presence of the indole moiety in indolo[1,2-*c*]quinazolin-6(5*H*)-one (**1**), the design of novel gramine-like analogues via aminomethyl substitution at position 12 is feasible.

**Scheme 3:**
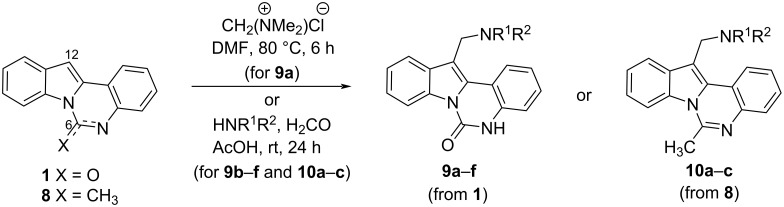
Mannich aminomethylation of indolo[1,2-*c*]quinazolines **1** and **8**.

To evaluate the influence of the urea carbonyl group on biological activity, a modified scaffold, 6-methylindolo[1,2-*c*]quinazoline (**8**) [26], was also used in which the carbonyl oxygen at position 6 is replaced by a methyl group. Substitution at position C6 will enable to investigate the influence of electronic and steric changes for target affinity, while retaining the key pharmacophoric features of the indoloquinazolinone core. Thus, a series of 12-aminomethyl derivatives **9a–f** and **10a–c** were synthesized from indolo[1,2-*c*]quinazolines **1** or **8**, respectively, via a Mannich reaction using Eschenmoser’s salt or by a mixture of formaldehyde and the corresponding amine in acetic acid [27] (Scheme 3, Table 1).

**Table 1:** Structure and yield of 12-aminomethyl derivatives of indolo[1,2-*c*]quinazolines **9a–f** and **10a–c**.

Compd.	NR^1^R^2^	Yield, %

**9a**	(CH_3_)_2_N	75
**9b**	(CH_3_CH_2_)_2_N	30
**9c**	pyrollidin-1-yl	78
**9d**	piperidine-1-yl	73
**9e**	4-methylpiperazin-1-yl	75
**9f**	4-(piperidin-1-yl)piperidin-1-yl	61
**10a**	(CH_3_)_2_N	30
**10b**	pyrrolidin-1-yl	76
**10c**	morpholin-1-yl	58

**Scheme 4:**
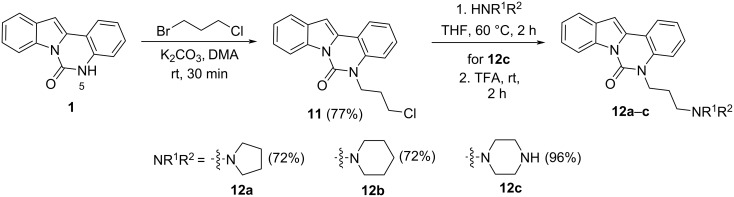
Synthesis of 5-(3-aminopropyl) derivatives of indolo[1,2-*c*]quinazolin-6(5*H*)-one **12a–c**.

The C12 and N5 positions of the indolo[1,2-*c*]quinazolin-6(5*H*)-one core serve as additional strategic handles for functionalization. Their synthetic versatility enables structural diversification, supporting comprehensive SAR investigations to explore pharmacological potential of this chemotype. The presence of an acidic NH proton in the urea moiety (position N5) of indolo[1,2-*c*]quinazolin-6(5*H*)-one (**1**) enables efficient *N*-alkylation. Accordingly, alkylation of **1** with 1-bromo-3-chloropropane afforded intermediate **11**, bearing a reactive chloropropyl side chain suitable for further derivatization. Nucleophilic substitution of the terminal chloride in **11** with various cyclic amines, including pyrrolidine, piperidine, and mono-*tert*-butoxycarbonyl (Boc)-protected piperazine, provided a set of aminoalkyl derivatives **12а**–**с** (Scheme 4). This strategy enables the expansion of structural diversity within this scaffold and facilitates exploration of SAR study related to the position and nature of the aminoalkyl substituent.

**Scheme 5:**
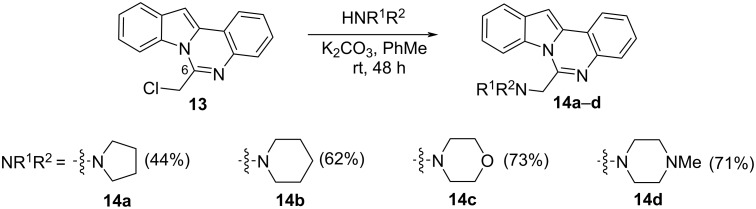
Synthesis of derivatives of 6-(aminomethyl)indolo[1,2-*c*]quinazolines **14a**–**d**.

Shifting the aminoalkyl substituent from position 12 to position 6 enables a comparative assessment of biological activity across the series. In this context, the synthesis of 6-(chloromethyl)indolo[1,2-*c*]quinazoline (**13**) was of particular interest, as the presence of a reactive chloromethyl group facilitates nucleophilic substitution with various amines. The compound **13** was synthesized according to a procedure described in the literature [28]. Subsequent displacement of the chloride in **13** with amines provided a series of 6-aminomethyl-substituted indoloquinazoline derivatives **14а–d** (Scheme 5).

The structures of all compounds were analyzed and confirmed by NMR and HRMS methods. Samples of all indolo[1,2-*c*]quinazoline derivatives with good analytical purity (HPLC, ≥95%) were further subjected to biological evaluation.

The antiproliferative activity of the novel indolo[1,2-*c*]quinazoline derivatives was evaluated against several human cancer cell lines, including colon carcinoma HCT116, lung adenocarcinoma A549, and chronic myeloid leukemia K562. To estimate selectivity for non-malignant cells human skin fibroblasts (HSF) were taken. Doxorubicin (**Dox**) was used as the positive control in all experiments.

Parent scaffold **1** and its 12-modified derivatives **2**–**4** showed no cytotoxicity against all tested cell lines (Table 2). Notably, compound **5** also exhibited no antiproliferative activity, in contrast to its isomer – the alkaloid tryptanthrin [17], indicating the importance of ring annelation sequence. In contrast, indolo[1,2-*c*]quinazolin-6(5*H*)-one (**1**), its unmodified 6-methyl analog **8**, and 6-chloromethyl analog **13** demonstrated a more pronounced, though still moderate, effect on cell growth (Table 3).

**Table 2:** Antiproliferative activity (MTT test, 72 h, IC_50_, μM ) of indolo[1,2-*c*]quinazoline derivatives and the reference drug Dox.

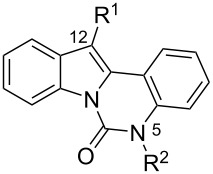						

			Cell line			
			
Compd.	R^1^	R^2^	K562	HCT116	A549	HSF

**1**	H	H	>50.0	>50.0	>50.0	>50.0
**2**	CHO	H	>50.0	>50.0	>50.0	>50.0
**3**	CO_2_H	H	>50.0	>50.0	>50.0	>50.0
**4**	OCHO	H	>50.0	>50.0	>50.0	>50.0
**5**	O	H	>50.0	>50.0	>50.0	>50.0
**9a**	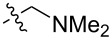	H	5.5 ± 0.7	4.9 ± 0.7	4.0 ± 0.5	5.0 ± 0.7
**9b**	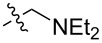	H	>50.0	43.0 ± 5.6	46.0 ± 6.4	>50.0
**9c**	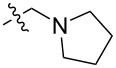	H	6.4 ± 0.9	1.0 ± 0.1	4.3 ± 0.6	19.0 ± 2.7
**9d**	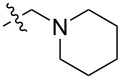	H	15.0 ± 1.8	21.0 ± 2.7	8.3 ± 1.1	17.0 ± 2.2
**9e**	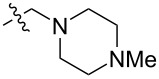	H	8.2 ± 1.1	18.0 ± 2.5	3.2 ± 0.4	14.0 ± 2.0
**9f**	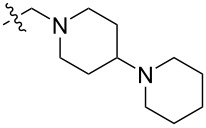	H	5.3 ± 0.7	7.5 ± 1.1	5.7 ± 0.7	13.6 ± 0.2
**12a**	H	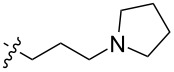	6.2 ± 0.9	5.7 ± 0.7	8.3 ± 1.2	4.4 ± 0.5
**12b**	H	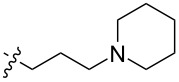	6.5 ± 0.9	5.7 ± 0.8	7.9 ± 1.03	5.0 ± 0.7
**12c**	H	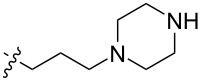	3.2 ± 0.4	3.2 ± 0.5	4.4 ± 0.6	2.4 ± 0.3
Dox			<0.10	0.30 ± 0.04	0.45 ± 0.06	0.15 ± 0.02

**Table 3:** Antiproliferative activity (MTT test, 72 h, IC_50_, μM) of indolo[1,2-*c*]quinazoline derivatives and the reference drug Dox.

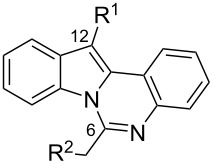						

			Cell line			
			
Compd.	R^1^	R^2^	K562	HCT116	A549	HSF

**8**	H	H	11.2 ± 1.5	7.4 ± 0.9	24.0 ± 3.1	13.0 ± 1.7
**13**	H	Cl	7.3 ± 0.9	10.8 ± 1.4	21.0 ± 2.9	8.1 ± 1.1
**10a**	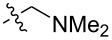	H	6.8 ± 0.8	9.0 ± 1.2	6.20 ± 0.8	14.8 ± 1.9
**10b**	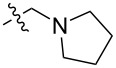	H	2.7 ± 0.3	2.0 ± 0.3	4.8 ± 0.7	3.7 ± 0.5
**10c**	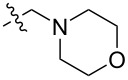	H	45.0 ± 5.9	50.0 ± 7.0	47.0 ± 6.1	>50.0
**14a**	H	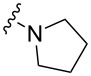	2.4 ± 0.3	4.5 ± 0.6	5.1 ± 0.6	3.5 ± 0.5
**14b**	H	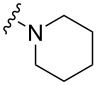	8.2 ± 1.0	9.2 ± 0.6	16.7 ± 2.3	8.4 ± 1.1
**14c**	H	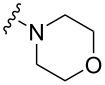	25.0 ± 3.5	33.0 ± 4.3	50.0 ± 7.0	>50.0
**14d**	H	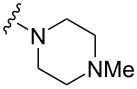	11.0 ± 1.3	8.8 ± 1.0	14.5 ± 1.7	6.5 ± 0.8
Dox			<0.10	0.30 ± 0.04	0.45 ± 0.06	0.15 ± 0.02

The introduction of a substituted aminomethyl group at position 12 of the indolo[1,2-*c*]quinazolin-6(5*H*)-one scaffold conferred significant cytotoxic efficacy. Gramine analog **9a** and the majority of compounds from this series exhibited notable antiproliferative activity, with a micromolar range of IC_50_ values towards all tested cancer cell lines. However, no clear correlation between structure and activity was observed. For example, elongation of the alkyl substituents (*N*,*N*-diethylamino derivative **9b**) led to a complete loss of antiproliferative activity, while its cyclic analog **9c** showed the lowest IC_50_ value in the series for the HCT116 cell line. Incorporation of an additional aminoalkyl moiety in the structure of cyclic amine (compounds **9e**, **9f**) does not result in any meaningful changes of potency. The same modification of 6-methylindolo[1,2-*c*]quinazoline scaffold yielding derivatives **10a**–**c** also gave superior results compared to the parent structure and again pyrrolidine as the key part of the 12-aminomethyl fragment (in **10b**) demonstrated the best activity.

The introduction of an aminopropyl group at the position 5 of indolo[1,2-*c*]quinazolin-6(5*H*)-one (derivatives **12a**–**c**) is also accompanied by the emergence of antiproliferative activity, which is not strongly affected by the structure of the terminal cyclic amine. Replacement of the substituents from position 12 to position 6 of 6-methylindolo[1,2-*c*]quinazoline core (compounds **14a**–**d**) leads to increased cytotoxicity compared to the initial structure. However, the antiproliferative potency of 12 and 6-funtionalized derivatives with the same cyclic amine, **10b** and **14a**, respectively, are close (Table 3).

As evidenced by their comparable cytotoxicity in non-malignant human fibroblasts, almost all tested compounds demonstrated a general lack of selectivity. Among the four types of indolo[1,2-*c*]quinazoline derivatives evaluated, only the 12-aminomethyl derivatives of indolo[1,2-*c*]quinazolin-6(*5H*)-one exhibited some degree of therapeutic selectivity. Specifically, compound **9c** showed a pronounced, 3–19-fold selectivity for tumor cells over non-tumor cells. Comparative analysis of the most active derivative **9c** (Table 2) with its structural analogue **10b** (Table 3) revealed that replacing the carbonyl group with a methyl moiety appears to increase the non-specific cytotoxicity of this chemotype, thereby reducing its selectivity.

**Figure 2:**
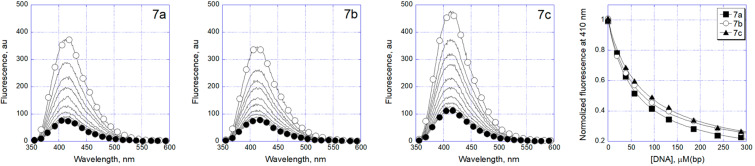
Fluorescence quenching of compounds **7a**–**c** (2 μM) upon titration with calf thymus DNA (0–290 μM base pairs) in 100 mM KCl, 20 mM Tris-HCl, pH 8.0.

We also estimated the potency of the new indolo[1,2-*c*]quinazoline derivatives to interact with nucleic acids. Since not all compounds exhibited intrinsic fluorescence, we initially performed a screening using the fluorescent intercalator displacement (FID) assay. Only compounds **7a**–**c** showed a positive response and interaction with DNA (Figure S1 in Supporting Information File 1). The interaction of compounds **7a**–**c** with duplex DNA was then investigated by fluorescence titration, measuring the quenching of ligand fluorescence upon binding to the nucleic acid. Fluorimetric titration studies confirmed the interaction of **7a**–**c** with double-stranded calf thymus DNA, as evidenced by ligand fluorescence quenching (Figure 2). Elongation of the linker in the carboxamide residue is accompanied by weaker DNA complexation (Table 4). The interaction of the compounds with DNA was found to be largely dependent on electrostatic forces. Reducing the ionic strength of the solution to 20 mM Tris buffer significantly enhanced DNA binding (Figure S2 in Supporting Information File 1). Notably, the spectral characteristics of the compounds remained virtually unchanged upon DNA binding. Scatchard plot analysis of the binding isotherms revealed a binding stoichiometry of one ligand per two base pairs at maximal DNA saturation. While the DNA binding affinity increased by an order of magnitude at low ionic strength, the characteristic decrease in affinity with longer linker lengths was still maintained. A similar trend was observed in the results of MTT assay: indolo[1,2-*c*]quinazoline derivative **7c** bearing a 4-aminobutyl substituent showed weaker DNA binding and cell growth inhibition.

**Table 4:** Antiproliferative activity (MTT test, 72 h, IC_50_, μM) and DNA binding (*K*_D_, μM) of 6-oxoindolo[1,2-*c*]quinazoline-12-carboxamides **7a**–**c** and the reference drug Dox.

			Cell line			
			
Compd.	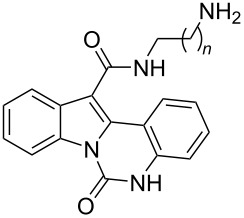	KD, µM (100 mM KCl)	K562	HCT116	A549	HSF

**7a**	*n* = 1	57 ± 3	27.0 ± 3.5	25.0 ± 3.3	13.5 ± 1.9	>50.0
**7b**	*n* = 2	60 ± 3	39.0 ± 5.1	32.0 ± 3.8	19.5 ± 2.7	>50.0
**7c**	*n* = 3	74 ± 4	>50.0	30.0 ± 4.2	20.0 ± 2.6	>50.0
Dox		0.7	<0.10	0.30 ± 0.04	0.45 ± 0.06	0.15 ± 0.02

Moreover, the measured dissociation constants for the most active derivatives indicated a lack of significant binding to dupex DNA, suggesting that their cytotoxic effects on tumor cells may proceed via an alternative, DNA-independent mechanism.

## Conclusion

In this study, a diverse set of indolo[1,2-*c*]quinazoline derivatives was synthesized through functionalization at positions 5, 6, and 12 of the polyannelated scaffold. The work highlights the synthetic versatility of the indolo[1,2-*c*]quinazoline framework, which enabled the generation of carboxamides, aminomethylated analogues using classical and advanced methodologies. Among these, 12-substituted aminomethyl derivatives exhibited the most promising antiproliferative activity, with compound **9c** showing notable cytotoxicity and reasonable selectivity toward cancer cells over non-malignant fibroblasts. In contrast, derivatives modified at positions 5 and 6 also demonstrated cytotoxic potential, but without a significant improvement in selectivity.

Fluorescence titration assays revealed that only the 12-carboxamide derivatives **7a**–**c** of indolo[1,2-*c*]quinazolin-6(5*H*)-one possess detectable interactions with double-stranded DNA. Notably, despite the planar polycyclic structure and emergence of the terminal basic center and a part of introduced side chain of these compounds, typically favorable for DNA intercalation, the results suggest that DNA is not the primary biological target of most derivatives in this series.

Importantly, this is the first study to demonstrate the antitumor potential of indolo[1,2-*c*]quinazoline derivatives. These findings establish this chemotype as a promising scaffold for anticancer drug development. Further investigations are warranted to elucidate their precise mechanism of action and optimize their therapeutic profile.

## Supporting Information

File 1 Experimental section.

## Data Availability

Data generated and analyzed during this study is available from the corresponding author upon reasonable request.
