# Supplementary material for: Discovery of cytotoxic indolo[1,2-c]quinazoline derivatives through scaffold-based design
Source: Beilstein J Org Chem. 2025 Oct 13;21:2062–71. doi: 10.3762/bjoc.21.161 (PMC12536455; doi:10.3762/bjoc.21.161)
Supplement: File 1 — Experimental section. [file Beilstein_J_Org_Chem-21-2062-s001.pdf]

BEILSTEIN JOURNAL OF ORGANIC CHEMISTRY

Supporting Information

for

Discovery of cytotoxic indolo[1,2-*c*]quinazoline derivatives through scaffold-based design

Daniil V. Khabarov, Valeria A. Litvinova, Lyubov G. Dezhenkova, Dmitry N. Kaluzhny, Alexander S. Tikhomirov and Andrey E. Shchekotikhin

Beilstein J. Org. Chem. **2025,** *21,* 2062–2071. doi:10.3762/bjoc.21.161

Experimental section

License and Terms: This is a supporting information file under the terms of the Creative Commons Attribution License (https://creativecommons.org/licenses/by/4.0). Please note that the reuse, redistribution and reproduction in particular requires that the author(s) and source are credited and that individual graphics may be subject to special legal provisions.
The license is subject to the *Beilstein Journal of Organic Chemistry* terms and conditions: (https://www.beilstein-journals.org/bjoc/terms)

Instruments, general information and synthetic procedures

NMR spectra were recorded on a Varian Mercury 400 Plus instrument operated at 400 MHz (^{1}H NMR) and 100 MHz (^{13}C NMR). Chemical shifts were measured in $CDCl_3$, CD_3OD or DMSO-d_6 using the residual solvent peak as a standard. High-resolution mass spectra were recorded with electron spray ionization on a Bruker Daltonics microOTOF-QII instrument. Melting points were determined using a Büchi SMP-20 apparatus and are given uncorrected. Analytical TLC was performed on Silica Gel F254 plates (Merck). Column chromatography was performed using SilicaGel Merck 60. Analysis of the purity was performed by HPLC on a Shimadzu LC-20 AD chromatograph, Kromasil-100-5-µm C-18 column (4.6 × 250 mm), LW = 210 nm using the system: A: 0.01 M H_3PO_4, pH 2.6; B: MeCN. All solutions were dried over Na_2SO_4 and evaporated at a reduced pressure using IKA RV 10 rotary evaporator at <45 °C. All products were vacuum-dried at room temperature. All solvents, chemicals and reagents were obtained from Sigma-Aldrich, St. Louis, MO unless specified otherwise, and used without purification.

*6-Oxo-5,6-dihydroindolo[1,2-c]quinazoline-12-carbaldehyde (**2**)*

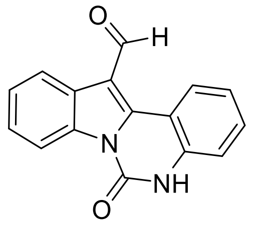

$POCl_3$ (0.25 mL, 2.68 mmol) was added dropwise to DMF (0.65 mL) at 0 °C and the mixture was stirred for 30 min. A solution of indolo[1,2-*c*]quinazolin-6(5*H*)-one (**1**) [1] (0.10 g, 0.43 mmol) in DMF (2.0 mL) was added to the generated Vilsmeier reagent and the reaction mixture was stirred at room temperature for 1 h. The mixture was poured into crushed ice and quenched with 20% aqueous NaOH solution to pH 8. The resulting precipitate was filtered off and dried under reduced pressure. Yield of **2** is 0.10 g (92%) as a yellow solid, mp 273-274 °C. HPLC: gradient B 30/70% (30 min) t_R = 21.1 min, purity 97%. ^{1}H NMR (400 MHz, DMSO-d_6) δ, ppm: 11.78 (s, 1H, NH), 10.78 (s, 1H, CHO), 8.71 (d, J = 8.2 Hz, 1H, CH), 8.66–8.57 (m, 1H, CH), 8.37

(dd, J = 6.5, 2.4 Hz, 1H, CH), 7.59 (t, J = 7.7 Hz, 1H, CH), 7.48–7.36 (m, 2H, CH), 7.33 (d, J = 8.2 Hz, 1H, CH), 7.29 (t, J = 7.7 Hz, 1H, CH). ^{13}C NMR (100 MHz, DMSO-d_6) δ, ppm: 186.4, 146.9, 140.5, 136.7, 133.4, 132.5, 128.1, 127.6, 125.7, 125.2, 123.9, 120.9, 116.4, 116.4, 113.7, 112.9. HRMS (ESI) calcd for $C_{16}H_{11}N_2O_2$, 263.0815 [M+H]$^+$; found, 263.0829.

*6-Oxo-5,6-dihydroindolo[1,2-c]quinazoline-12-carboxylic acid (**3**)*

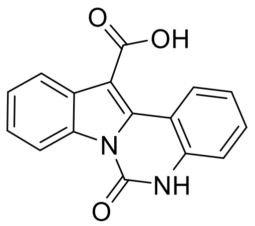

Method A. A solution of $K_2Cr_2O_7$ (10 mg, 3.4 mmol) and conc. H_2SO_4 (8 mL, 14.7 mmol) in H_2O (1.0 mL) was added dropwise to a solution of compound **2** (0.10 g, 0.38 mmol) in AcOH (28.0 mL). The reaction mixture was stirred at room temperature for 3 h, the resulting green precipitate was filtered off, washed with water (3 × 5 mL), and dried under reduced pressure. Yield of **3** is 11% (0.12 mg).

Method B. Aqueous solution of NaOH (10 mass %, 15.0 mL) was added to a solution of compound **3** (0.60 g, 1.82 mmol) in dioxane (18.0 mL). The reaction mixture was stirred at 60 °C for 6 h. The solution was cooled and neutralized with 5% aqueous HCl to pH 6, the precipitate was filtered off and dried under reduced pressure. Yield of **3** is 0.45g (90%) as a yellow solid, mp 284-286 °C. HPLC: gradient B 30/70% (30 min) t_R = 14.9 min, purity 96%. ^{1}H NMR (400 MHz, DMSO-d_6) δ, ppm: 11.80 (s, 1H, NH), 9.25–9.12 (m, 1H, CH), 8.70 (dd, J = 6.7, 2.5 Hz, 1H, CH), 8.21–8.09 (m, 1H, CH), 7.57–7.47 (m, 1H, CH), 7.45–7.38 (m, 2H, CH), 7.35–7.19 (m, 2H, CH). ^{13}C NMR (100 MHz, DMSO-d_6) δ, ppm: 167.1, 147.2, 136.7, 136.0, 133.0, 131.4, 128.9, 127.9, 124.8, 124.4, 123.0, 121.7, 116.3, 115.9, 113.1, 107.1. HRMS (ESI) calcd for $C_{16}H_{11}N_2O_3$, 279.0764 [M+H]$^+$; found, 279.0780.

*6-Oxo-5,6-dihydroindolo[1,2-c]quinazolin-12-yl formate (**4**)*

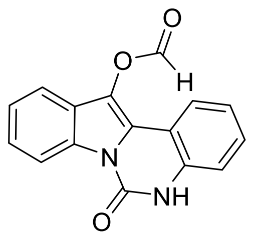

To a solution of compound **2** (50 mg, 0.20 mmol) in DMF (2.0 mL) was added Oxone (0.10 g, 0.60 mmol) and the reaction mixture was stirred at room temperature for 18 h. The reaction mixture was diluted with H_2O (5.0 mL), the resulted precipitate was filtrated off and dried under reduced pressure. Yield of **4** is 43 mg (77%) as a white solid, mp 237-239 °C. HPLC: gradient B 30/70% (30 min) t_R = 22.1 min, purity 97%. ^{1}H NMR (400 MHz, DMSO-*d*$_6$) δ, ppm: 11.47 (s, 1H, NH), 8.83 (s, 1H, CH), 8.63–8.56 (m, 1H, CH), 8.09–8.03 (m, 1H, CH), 7.60–7.52 (m, 1H, CH), 7.49–7.32 (m, 3H, CH), 7.32–7.19 (m, 2H, CH). ^{13}C NMR (100 MHz, DMSO-*d*$_6$) δ, ppm: 161.0, 147.1, 134.8, 130.7, 130.1, 125.6, 124.6, 124.1, 124.1, 123.6, 123.5, 122.5, 117.7, 116.1, 115.8, 112.6. HRMS (ESI) calcd for $C_{16}H_{11}N_2O_3$, 279.0764 $[M+H]^+$; found, 279.0772.

*5,12a-Dihydroindolo[1,2-c]quinazoline-6,12-dione (**5**)*

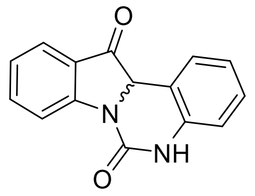

To a solution of compound **4** (0.53 g, 1.9 mmol) in THF (35 mL) was added saturated aqueous $NaHCO_3$ (11 mL) and refluxed for 3 h. The solution was concentrated under reduced pressure, diluted with EtOAc (20 mL), organic layer washed with water (2 × 10 mL), dried and concentrated under reduced pressure. The crude residue was purified by column chromatography, using a toluene/EtOAc 6:1 gradient as eluent. Yield of **5** is 110 mg (23%) as a white solid, mp 160-163 °C (decomp.). HPLC: gradient B 30/70% (30 min) t_R = 12.1 min, purity 99%. ^{1}H NMR (400 MHz, DMSO-*d*$_6$) δ, ppm: δ 10.41 (s, 1H, NH), 8.13–8.02 (m, 1H, CH), 7.92-7.90 (m, 1H, CH), 7.79–7.67 (m, 2H, 2CH), 7.65 (s, 1H, CH), 7.34-7.30 (m, 1H, CH), 7.27–7.10 (m, 2H, 2CH), 7.08–

6.98 (m, 2H, 2CH). ^{13}C NMR (100 MHz, DMSO-d_6) δ, ppm: 196.2, 151.9, 149.4, 138.6, 138.1, 130.3, 125.2 (2C), 124.0, 122.3, 120.2, 119.5, 116.1, 115.4, 82.7. HRMS (ESI) calcd for $C_{15}H_{11}N_2O_2$, 251.0815 $[M+H]^+$; found, 251.0802.

*12-(2,2,2-Trifluoroacetyl)indolo[1,2-c]quinazolin-6(5H)-one (**6**)*

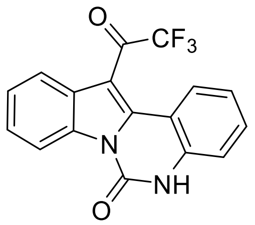

A solution of compound **1** (0.10 g, 0.43 mmol) in TFA (2.0 mL) and TFAA (0.40 mL, 1.42 mmol) was boiled for 40 min, cooled to room temperature, and concentrated under reduced pressure. Yield of **6** is 0.14 g (98%) as a yellow solid, mp 239-240 °C. HPLC: gradient B 50/90% (30 min) t_R = 18.8 min, purity 95%. ^1H NMR (400 MHz, DMSO-d_6) δ, ppm: 12.13 (s, 1H, NH), 8.77–8.69 (m, 1H, CH), 8.18 (d, J = 8.3 Hz, 1H, CH), 7.77 (d, J = 7.3 Hz, 1H, CH), 7.59 (t, J = 7.6 Hz, 1H, CH), 7.51–7.43 (m, 2H, CH), 7.34 (d, J = 8.2 Hz, 1H, CH), 7.24 (t, J = 7.8 Hz, 1H, CH). ^{13}C NMR (100 MHz, DMSO-d_6) δ, ppm: 180.8 (d, J = 36.0 Hz), 146.5, 140.5, 136.8, 133.2, 133.0, 126.8, 126.1, 125.7, 125.2, 123.2, 119.4 (d, J = 3.8 Hz), 117.0, 116.8 (q, J = 292.2 Hz), 116.4, 112.0, 106.7. HRMS (ESI) calcd for $C_{17}H_{10}F_3N_2O_2$, 331.0689 $[M+H]^+$; found, 331.0693.

*N-(2-Aminoethyl)-6-oxo-5,6-dihydroindolo[1,2-c]quinazoline-12-carboxamide (7**a**)*

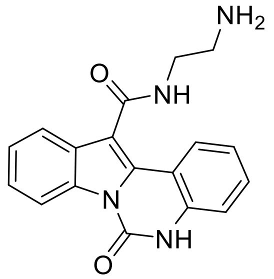

To a solution of compound **3** (50 mg, 0.19 mmol) and DIPEA (66 µL, 0.38 mmol) in DMSO (3.0 mL) were added PyBOP (148 mg, 0.38 mmol) and *tert*-butyl (2-aminoethyl)carbamate (61 mg, 0.38 mmol). The reaction mixture was stirred at room temperature for 1 h, diluted with

EtOAc (15 mL), organic layer washed with 5% aqueous HCl (5 mL), with saturated aqueous NaCl (2 × 5 mL), dried and concentrated under reduced pressure. The crude residue was purified by flash chromatography, eluent toluene/EtOAc 3:1. The Boc-protected intermediate was dissolved in TFA (1.0 mL, 19.6 mmol) and the solution stirred for 2 h at room temperature. The mixture concentrated under reduced pressure and the residue was dried. Yield of **7a** is 30 mg (58%) as a brown solid, mp 255-256 °C. HPLC: gradient B 10/50% (30 min) t_R = 14.1 min, purity 97%. ^1H NMR (400 MHz, DMSO-d_6) δ, ppm: 11.59 (s, 1H, NH), 8.80 (t, *J* = 5.6 Hz, 1H, CH), 8.68–8.59 (m, 1H, CH), 8.30 (d, *J* = 8.1 Hz, 1H, CH), 8.01 (s, 3H,NH_3), 7.83–7.79 (m, 1H, NH), 7.50–7.45 (m, 1H, CH), 7.44–7.40 (m, *J* = 7.3, 3.7 Hz, 2H, CH), 7.31 (d, *J* = 8.1 Hz, 1H, CH), 7.22 (t, *J* = 7.7 Hz, 1H, CH), 3.66 (q, *J* = 6.4 Hz, 2H, CH_2), 3.09 (t, *J* = 6.8 Hz, 2H, CH_2). ^{13}C NMR (100 MHz, DMSO-d_6) δ, ppm: 165.7, 147.3, 135.4, 132.7, 131.7, 130.7, 128.2, 125.9, 124.4, 124.3, 123.3, 120.1, 116.2, 116.0, 113.1, 110.5, 38.9, 37.6. HRMS (ESI) calcd for $C_{18}H_{17}N_4O_2$, 321.1346 $[M+H]^+$; found, 321.1335.

*N-(3-Aminopropyl)-6-oxo-5,6-dihydroindolo[1,2-c]quinazoline-12-carboxamide (7**b**)*

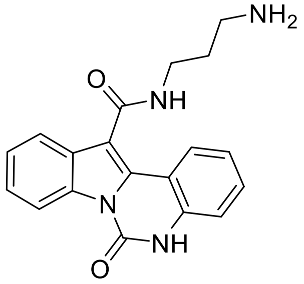

Compound **7b** was prepared from compound **3** as described for derivative **7a**. Yield of **7b** is 34 mg (65%) as a brown solid, mp 258-259 °C. HPLC: gradient B 10/50% (30 min) t_R = 14.5 min, purity 96% ^1H NMR (400 MHz, DMSO-d_6) δ, ppm: 11.63 (s, 1H, NH), 8.84 (t, *J* = 5.7 Hz, 1H, CH), 8.63–8.60 (m, 1H, CH), 8.19 (d, *J* = 8.1 Hz, 1H, CH), 7.91 (s, 3H, NH_2), 7.71-7.68 (m, *J* = 6.2, 3.1 Hz 1H, NH), 7.52 – 7.40 (m, 2H, CH), 7.41 (d, *J* = 3.3 Hz, 1H, CH), 7.31 (d, *J* = 8.1 Hz, 1H, CH), 7.23 (t, *J* = 7.6 Hz, 1H, CH), 3.47 (d, *J* = 6.1 Hz, 2H, CH_2), 2.92 (t, *J* = 7.6 Hz, 2H, CH_2), 1.91 (p, *J* = 6.9 Hz, 2H, CH_2). ^{13}C NMR (100 MHz, DMSO-d_6) δ, ppm: 165.5, 147.3, 135.2,

132.6, 131.1, 130.6, 128.4, 125.4, 124.4 (2C), 123.4, 119.6, 116.2, 116.1, 113.2, 110.9, 37.5, 36.8, 27.8. HRMS (ESI) calcd for $C_{19}H_{19}N_4O_2$ 335.1503 [M+H]$^+$; found, 335.1486.

*N-(4-Aminobutyl)-6-oxo-5,6-dihydroindolo[1,2-c]quinazoline-12-carboxamide (7**c**)*

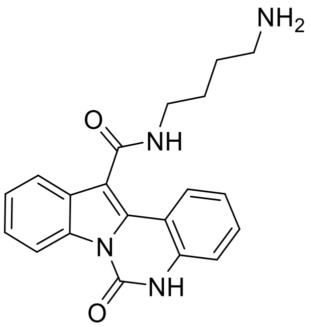

Compound **7c** was prepared from compound **3** as described for derivative **7a**. Yield of **7c** is 57 mg (62%) as a brown solid, mp 260-262 °C. HPLC: gradient B 10/50% (30 min) t_R = 14.8 min, purity 97%. ^{1}H NMR (400 MHz, DMSO-d_6) δ, ppm: 8.76 (t, J = 5.7 Hz, 1H, CH), 8.64-8.60 (m, 1H, CH), 8.22 (d, J = 8.0 Hz, 1H, CH), 7.89 (s, 3H, NH_2), 7.72-7.68 (m, 1H, NH), 7.51–7.43 (m, 1H, CH), 7.46–7.37 (m, 2H, CH), 7.31 (d, J = 8.1 Hz, 1H, CH), 7.24 (t, J = 7.7 Hz, 1H, CH), 3.41 (d, J = 5.6 Hz, 2H, CH), 2.87 (s, 2H, CH_2), 1.71–1.63 (m, 4H, CH_2). ^{13}C NMR (100 MHz, DMSO-d_6) δ, ppm: 165.1, 147.3, 135.2, 132.6, 131.0, 130.5, 128.5, 125.5, 124.3 (2C), 123.3, 119.7, 116.2, 116.0, 113.3, 111.3, 39.1, 39.0, 26.6, 25.2. HRMS (ESI) calcd for $C_{20}H_{21}N_4O_2$ 349.1659 [M+H]$^+$; found, 349.1673.

*12-((Dimethylamino)methyl)indolo[1,2-c]quinazolin-6(5H)-one (**9a**)*

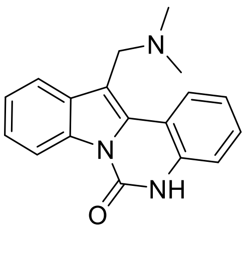

To a solution of compound **1** (0.10 g, 0.43 mmol) in DMF (2.0 mL) was added *N,N*-dimethylmethyleneiminium chloride (88 mg, 0.99 mmol). The mixture was stirred at 80 °C for 6 h, cooled to room temperature and carefully quenched with saturated aqueous $NaHCO_3$ (8 mL). The white precipitate was filtered off, washed with water (3 × 5 mL), and dried under reduced

pressure. Yield of **9a** is 93 mg (75%) as a white solid, mp 279-280 °C. HPLC: gradient B 10/50% (30 min) t_R = 16.8 min, purity 100%. ^{1}H NMR (400 MHz, DMSO-d_6) δ, ppm: 11.74 (s, 1H, NH), 10.34 (s, 1H, NH), 8.67–8.58 (m, 1H, CH), 8.37 (d, *J* = 8.1 Hz, 1H, CH), 8.12–8.05 (m, 1H, CH), 7.50 (t, *J* = 7.7 Hz, 1H, CH), 7.47–7.34 (m, 3H, CH), 7.28 (t, *J* = 7.6 Hz, 1H, CH), 4.99 (s, 2H, CH_2), 2.88–2.83 (m, 6H, CH_3). ^{13}C NMR (100 MHz, DMSO-d_6) δ, ppm: 147.2, 135.7, 133.8, 132.9, 130.6, 130.5, 125.8, 124.5, 124.1, 123.5, 119.4, 116.2, 116.2, 113.4, 50.3, 42.6 (2C), 34.4. HRMS (ESI) calcd for $C_{18}H_{18}N_3O$, 292.1444 $[M+H]^+$; found, 292.1453.

*12-((Diethylamino)methyl)indolo[1,2-c]quinazolin-6(5H)-one (**9b**)*

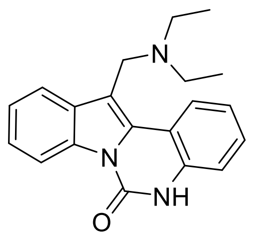

To a solution of compound **1** (50 mg, 0.21 mmol) in acetic acid (10 mL) were added diethylamine (0.1 mL, 1.28 mmol) and 37% aqueous formaldehyde (25 µL, 0.32 mmol). The reaction mixture was stirred at room temperature for 24 h, and concentrated under reduced pressure. The residue was diluted with saturated aqueous $NaHCO_3$ (10.0 mL), the residue was filtered off, washed with water (1 × 5 mL), and dried under reduced pressure. Yield of **9b** is 20 mg (30%) as a white solid, mp 264-265 °C (decomp.). HPLC: gradient B 10/50% (30 min) t_R = 19.1 min, purity 95%. ^{1}H NMR (400 MHz, DMSO-d_6) δ, ppm: 11.77 (s, 1H, NH), 9.76 (s, 1H, NH), 8.69–8.60 (m, 1H, CH), 8.28 (d, *J* = 8.2 Hz, 1H), 8.07–7.99 (m, 1H, CH), 7.57–7.35 (m, 4H, CH), 7.31 (t, *J* = 7.7 Hz, 1H, CH), 5.01 (d, *J* = 5.4 Hz, 2H, CH), 3.39–3.27 (m, 2H, CH_2), 3.27–3.16 (m, 2H, CH_2), 1.31 (t, *J* = 7.1 Hz, 6H, CH_3). ^{13}C NMR (100 MHz, DMSO-d_6) δ, ppm: 147.2, 135.7, 133.8, 132.9, 130.6 (2C), 125.6, 124.6, 124.2, 123.4, 119.3, 116.3, 116.3, 113.5, 102.6, 47.3, 47.0 (2C), 9.2 (2C). HRMS (ESI) calcd for $C_{20}H_{22}N_3O$, 320.1757 $[M+H]^+$; found, 320.1773.

*12-(Pyrrolidin-1-ylmethyl)indolo[1,2-c]quinazolin-6(5H)-one **(9c)***

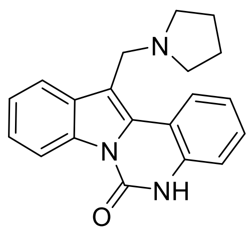

Compound **9c** was prepared from compound **1** as described for derivative **9b**. Yield of **9c** is 53 mg (78%) as a white solid, mp 258-260 °C. HPLC: gradient B 30/70% (30 min) t_R = 4.8 min, purity 98%. ^{1}H NMR (400 MHz, DMSO-d_6) δ, ppm: 11.37 (s, 1H, NH), 8.63–8.54 (m, 1H, CH), 8.32–8.25 (m, 1H, CH), 7.90–7.82 (m, 1H, CH), 7.44–7.34 (m, 2H, CH), 7.34 (d, J = 4.2 Hz, 1H, CH), 7.24 (dd, J = 8.0, 5.7 Hz, 2H, CH), 4.01 (s, 2H, CH_2), 2.52 (d, J = 5.6 Hz, 4H, CH_2), 1.65 (q, J = 3.3 Hz, 4H, CH_2). ^{13}C NMR (100 MHz, DMSO-d_6) δ, ppm: 147.6, 135.1, 132.6, 131.1, 130.9, 129.3 (C), 126.6, 123.7, 123.6, 123.3, 118.7, 116.0, 115.5, 114.8, 111.8, 54.0 (2C), 49.2, 23.6 (2C). HRMS (ESI) calcd for C20H20N3O, 318.1601 [M+H]+; found, 318.1613.

*12-(Piperidin-1-ylmethyl)indolo[1,2-c]quinazolin-6(5H)-one **(9d)***

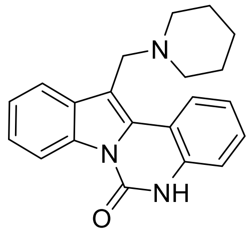

Compound **9d** was prepared from compound **1** as described for derivative **9b**. Yield of **9d** is 50 mg (73%) as a white solid, mp 254-256 °C. HPLC: gradient B 30/70% (30 min) t_R = 5.9 min, purity 97%. ^{1}H NMR (400 MHz, DMSO-d_6) δ, ppm: 11.35 (s, 1H, NH), 8.61–8.54 (m, 1H, CH), 8.30 (d, J = 7.9 Hz, 1H, CH), 7.81 (d, J = 7.0 Hz, 1H, CH), 7.44–7.30 (m, 3H, CH), 7.24 (t, J = 7.8 Hz, 2H, CH), 3.84 (s, 2H, CH_2), 2.45 (s, 4H, CH_2), 1.46–1.35 (m, 6H, CH_2). ^{13}C NMR (100 MHz, DMSO-d_6) δ, ppm: 147.6, 135.2, 132.6, 131.6, 131.3 (C), 129.4, 126.8, 123.7, 123.7, 123.2, 118.8, 116.0, 115.5, 114.7, 110.9, 54.3 (2C), 52.6, 26.1 (2C), 24.5. HRMS (ESI) calcd for $C_{21}H_{22}N_3O$, 332.1757 $[M+H]^+$; found, 332.1743.

*12-((4-Methylpiperazin-1-yl)methyl)indolo[1,2-c]quinazolin-6(5H)-one (**9e**)*

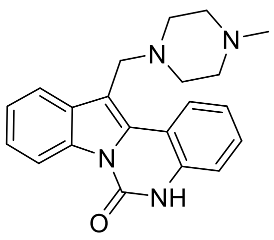

Compound **9e** was prepared from compound **1** as described for **9b**. Yield of **9e** is 55 mg (75%) as a white solid, mp 260 °C (decomp.). HPLC: gradient B 30/70% (30 min) t_R = 4.8 min, purity 96%. ^{1}H NMR (400 MHz, DMSO-d_6) δ, ppm: 11.42 (s, 1H, NH), 8.61–8.55 (m, 1H, CH), 8.28 (d, J = 8.0 Hz, 1H, CH), 7.86–7.79 (m, 1H, CH), 7.40 (t, J = 7.7 Hz, 1H, CH), 7.36–7.30 (m, J = 8.2, 4.0 Hz, 2H, CH), 7.24 (dd, J = 16.5, 8.1 Hz, 2H, CH), 3.88 (s, 2H, CH_2), 2.25 (s, 4H, CH_2), 2.09 (s, 3H, CH_2), 1.83 (s, 3H, CH_3). ^{13}C NMR (100 MHz, DMSO-d_6) δ, ppm: 173.4, 147.6, 135.2, 132.6, 131.5, 131.4, 129.4, 126.8, 123.7, 123.7, 123.2, 118.7, 116.0, 115.5, 114.7, 110.4, 55.2, 52.9, 51.8, 46.1, 22.7. HRMS (ESI) calcd for $C_{21}H_{23}N_4O$, 347.1866 [M+H]$^+$; found, 347.1881.

*12-([1,4'-Bipiperidin]-1'-ylmethyl)indolo[1,2-c]quinazolin-6(5H)-one (**9f**)*

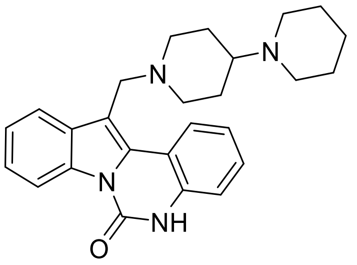

Compound **9f** was prepared from compound **1** as described for **9b**. Yield of **9f** is 53 mg (61%) as a white solid, mp 273-274 °C. HPLC: gradient B 10/50% (30 min) t_R = 12.2 min, purity 98%. ^{1}H NMR (400 MHz, DMSO-d_6) δ, ppm: 8.64–8.57 (m, 1H, CH), 8.31 (d, J = 8.0 Hz, 1H, CH), 7.84–7.78 (m, 1H, CH), 7.43–7.18 (m, 5H, CH), 3.88 (s, 2H, CH_2), 2.93 – 2.89 (m, 1H, CH), 2.36 (t, J = 5.1 Hz, 4H, CH_2), 2.22–2.04 (m, 4H, CH_2), 1.67–1.60 (m, 2H, CH_2), 1.44–1.29 (m, 8H, CH_2). ^{13}C NMR (100 MHz, DMSO-d_6) δ, ppm: 132.7, 131.6, 129.2, 126.7, 123.6, 123.5, 122.9, 118.7, 116.1, 62.5, 53.3 (3C), 52.0, 50.2 (3C), 28.3 (4C), 26.6 (4C), 25.0. HRMS (ESI) calcd for $C_{26}H_{31}N_4O$, 415.2492 [M+H]$^+$; found, 415.2498.

N,N-Dimethyl-1-(6-methylindolo[1,2-c]quinazolin-12-yl)methanamine (***10a***)

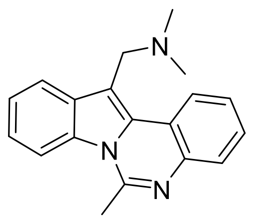

Compound **8** (0.2 g, 0.86 mmol) [2] was dissolved in acetic acid (20.0 mL), followed by the addition of 33% aqueous dimethylamine (40 µL, 0.28 mmol) and 37% aqueous formaldehyde (25 µL, 0.32 mmol). The reaction mixture was stirred for 24 h at room temperature. After completion, the mixture was concentrated under reduced pressure. Saturated aqueous $NaHCO_3$ (10.0 mL) was then added, and the resulting precipitate was collected by filtration. The residue was purified by column chromatography, eluent toluene/EtOAc 3:1. Yield of **10a** is 71 mg (30%) as a brown solid, mp 213-215 °C. HPLC (Gradient B 10/50% (30 min)) t_R = 16.0 min, purity 96%. ^{1}H NMR (400 MHz, CD_3OD) δ 8.70–8.63 (m, 1H, CH), 8.46 (d, *J* = 8.3 Hz, 1H, CH), 8.27 (dd, *J* = 7.6, 1.6 Hz, 1H, CH), 7.97–7.85 (m, 3H, CH), 7.85–7.72 (m, 2H, CH), 5.30 (s, 2H, CH_2), 3.55 (d, *J* = 4.9 Hz, 3H, CH_3), 3.09 (s, 6H, CH_3). ^{13}C NMR (100 MHz, CD_3OD) δ 156.5, 132.2, 131.7, 131.6, 131.3, 129.9, 129.8, 127.1, 126.2, 124.7, 119.6, 119.4, 117.8, 116.5, 106.0, 50.1, 42.3 (2C), 20.8. HRMS (ESI) calcd for $C_{19}H_{20}N_3$, 290.1652 [M+H]$^+$; found, 290.1664.

6-Methyl-12-(pyrrolidin-1-ylmethyl)indolo[1,2-c]quinazoline (***10b***)

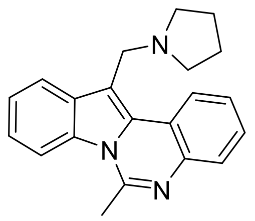

Compound **10b** was prepared from compound **8** as described for **10a**. Yield of **10b** is 45 mg (76%) as a brown solid, mp 133-135 °C. HPLC: gradient B 30/70% (30 min) t_R = 4.8 min, purity 96%. ^{1}H NMR (400 MHz, DMSO-d_6) δ, ppm: 8.47–8.38 (m, 1H, CH), 8.19 (d, *J* = 8.2 Hz, 1H, CH), 7.95 (d, *J* = 7.8 Hz, 1H, CH), 7.67–7.59 (m, 1H, CH), 7.55–7.48 (m, 2H, CH), 7.46–7.34 (m, 2H, CH), 4.06 (s, 2H, CH_2), 3.06 (s, 3H, CH_3), 2.54 (d, *J* = 5.7 Hz, 4H, CH_2), 1.65 (q, *J* = 3.3 Hz,

4H, CH_2). ^{13}C NMR (100 MHz, DMSO-d_6) δ, ppm: 149.0, 139.8, 131.2, 131.0, 130.4, 129.0, 127.0, 126.7, 126.0, 123.4, 122.7, 120.9, 118.8, 115.8, 109.0, 54.0 (2C), 49.3, 25.6, 23.6 (2C). HRMS (ESI) calcd for $C_{21}H_{22}N_3$, 316.1808 $[M+H]^+$; found, 316.1819.

*4-((6-Methylindolo[1,2-c]quinazolin-12-yl)methyl)morpholine (**10c**)*

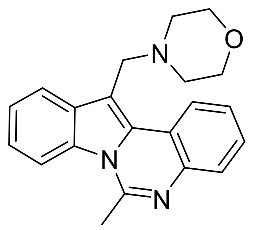

Compound **10c** was prepared from compound **8** as described for **10a**. Yield of **10c** is 40 mg (58%) as a brown solid, mp 174-175 °C. HPLC: gradient B 10/50% (30 min) t_R = 16.6 min, purity 97%. ^{1}H NMR (400 MHz, DMSO-d_6) δ, ppm: 8.46–8.43 (m, J = 8.0, 3.1 Hz, 1H, CH), 8.19 (d, J = 8.3 Hz, 1H, CH), 7.99–7.92 (m, 1H, CH), 7.69–7.61 (m, 1H, CH), 7.58–7.34 (m, 4H, CH), 3.95 (s, 2H, CH_2), 3.49 (t, J = 4.6 Hz, 4H, CH_2), 3.05 (s, 3H, CH_3), 2.52–2.45 (m, 4H, CH_2). ^{13}C NMR (100 MHz, DMSO-d_6) δ, ppm: 149.0, 139.9, 131.7, 131.5, 130.4, 129.1, 127.1, 126.8, 126.1, 123.6, 122.8, 120.8, 118.8, 115.9, 107.1, 66.7 (2C), 53.5 (2C), 52.3, 25.7. HRMS (ESI) calcd for $C_{21}H_{22}N_3O$, 332.1757 $[M+H]^+$; found, 332.1748.

*5-(3-Chloropropyl)indolo[1,2-c]quinazolin-6(5H)-one (**11**)*

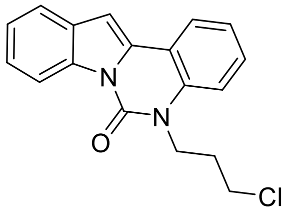

To a solution of compound **1** (0.15 g, 0.64 mmol) in DMA were added anhydrous potassium carbonate (0.16 g, 1.26 mmol) and 1-bromo-3-chloropropane (70 μL, 0.70 mmol). The reaction mixture was stirred at room temperature for 30 min, then quenched with 5% aqueous HCl solution. The product was extracted with ethyl acetate (2 × 20 mL), the combined organic layer was washed with saturated NaCl solution (3 × 20 mL), water (20 mL), dried over Na_2SO_4, and concentrated

under reduced pressure. The crude product was purified by column chromatography, using $CHCl_3$/MeOH (30:0.5) as eluent, followed by precipitation from DCM with petroleum ether (1:3). Yield of **11** is 0.15 g (77%) as a white solid, mp 120-124 °C. ^{1}H NMR (400 MHz, $CDCl_3$) δ, ppm: 8.71–8.63 (m, 1H), 7.93 (dd, *J* = 7.8, 1.5 Hz, 1H), 7.72–7.63 (m, 1H), 7.46–7.43 (m, 1H), 7.40–7.36 (m, 2H), 7.32–7.19 (m, 2H), 7.02 (s, 1H), 4.41 (dd, *J* = 8.6, 6.4 Hz, 2H), 3.74 (t, *J* = 6.2 Hz, 2H), 2.33–2.26 (m, 2H). ^{13}C NMR (100 MHz, $CDCl_3$) δ, ppm: 147.8, 134.5, 134.2, 133.0, 129.9, 129.4, 124.1, 123.8, 123.5, 123.2, 120.1, 116.2, 115.6, 114.1, 98.1, 42.6, 40.9, 30.3. HRMS (ESI) calcd for $C_{18}H_{15}ClN_2O$, 311.0946 $[M+H]^+$; found 311.0955.

5-(3-(Pyrrolidin-1-yl)propyl)indolo[1,2-c]quinazolin-6(5H)-one hydrochloride (***12a***)

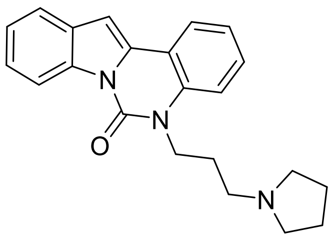

To a solution of compound **11** (50 mg, 0.16 mmol) in THF (10 mL) was added pyrrolidine (0.16 mL, 1.6 mmol) and was stirred 60 °C for 2 h. The reaction mixture was concentrated under reduced pressure. The crude residue was purified by column chromatography, using $CHCl_3$/MeOH 20:5 as eluent, followed by precipitation from DCM with petroleum ether (1:3). Yield of **12a** is 44 mg (72%) as a beige solid, mp 150-151°C. HPLC (LW = 265 nm, gradient B 40/95% (30 min)) t_R = 8.0 min, purity 99%. ^{1}H NMR (400 MHz, $CDCl_3$) δ 8.70 – 8.63 (m, 1H, CH), 7.94-7.92 (m, 1H, CH), 7.69-7.66 (m, 1H, CH), 7.42 (t, *J* = 7.9 Hz, 1H, CH), 7.39–7.28 (m, 3H, CH), 7.24–7.20 (m, 1H, CH), 7.03 (d, *J* = 3.0 Hz, 1H, CH), 4.39–4.30 (m, 2H, CH_2), 2.80 (t, *J* = 7.5 Hz, 2H, CH_2), 2.73 (d, *J* = 6.2 Hz, 6H, CH_2), 2.14 (br s, 2H, CH_2), 1.26 (d, *J* = 11.9 Hz, 2H, CH_2). ^{13}C NMR (100 MHz, $CDCl_3$) δ 147.9, 134.6, 134.2, 133.1, 129.9, 129.4, 124.0, 123.8, 123.4, 123.1, 120.1, 116.3, 115.5, 114.5, 98.0, 54.0 (2C), 53.4, 41.3, 26.3, 23.4 (2C). HRMS (ESI) calcd for $C_{22}H_{24}N_3O$, 346.1914 $[M+H]^+$; found, 346.1925.

5-(3-(Piperidin-1-yl)propyl)indolo[1,2-c]quinazolin-6(5H)-one hydrochloride (***12b***)

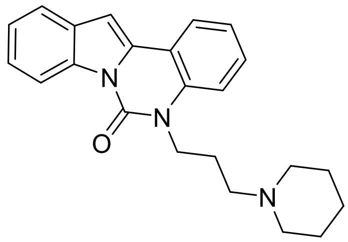

Compound **12b** was prepared from **1** as described for **12a**. Yield of **12b** is 44 mg (72%) as a beige solid, mp 132-135°C. HPLC: gradient B 40/95% (30 min) t_R = 9.5 min, purity 100%. ^{1}H NMR (400 MHz, $CDCl_3$) δ, ppm: 8.61 – 8.55 (m, 1H, CH), 7.90 (d, *J* = 7.2 Hz, 1H, CH), 7.68-7.66 (m, 1H, CH), 7.48 (s, 1H, CH), 7.39–7.32 (m, 2H, CH), 7.23 (s, 1H, CH), 7.03 (s, 1H, CH), 4.37 (s, 2H, CH_2), 3.56 (s, 2H, CH_2), 3.19 (s, 2H, CH_2), 2.63 (s, 2H, CH_2), 2.52 (s, 2H, CH_2), 2.26 (s, 2H, CH_2), 2.14 (s, 1H, CH_2), 1.85 (s, 3H, CH_2).^{13}C NMR (101 MHz, $CDCl_3$) δ, ppm: 148.1, 134.1, 134.0, 133.0, 130.0, 130.0, 124.1, 123.9, 123.6, 123.5, 120.2, 116.1, 115.4, 114.7, 98.4, 55.4, 53.7 (2C), 40.8, 22.9, 22.6 (2C), 22.1. HRMS (ESI) calcd for $C_{23}H_{26}N_3O$, 360.2070 $[M+H]^+$; found, 360.2081.

5-(3-(Piperazin-1-yl)propyl)indolo[1,2-c]quinazolin-6(5H)-one dihydrochloride (***12c***)

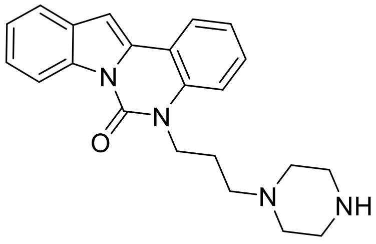

To a solution of compound **1** (50 mg, 0.16 mmol) in THF (10 mL) was added mono-Boc-piperazine (0.30 g, 1.6 mmol). The reaction mixture was refluxed for 2 h, cooled to room temperature, and poured into water acidified by HCl solution. The product was extracted with ethyl acetate (2 × 20 mL), the combined organic layer was washed with saturated NaCl solution (2 × 10 mL), dried over Na_2SO_4, and concentrated under reduced pressure. The crude residue was purified by column chromatography, using $CHCl_3$/MeOH 30:1 as eluent. The mono-Boc intermediate (74 mg, 0.16 mmol) was dissolved in chloroform (1 mL), TFA (61 µL, 0.80 mmol)

was added and the reaction mixture was stirred at room temperature for 2 h. The solvent was removed under reduced pressure. The resulting oily residue was treated with methanol (1 mL) and HCl in Et_2O (100 μL), and the mixture was stirred for 10 min. The reaction mixture was concentrated under reduced pressure. This crude material was triturated with acetone (10 mL) and diethyl ether (10 mL), with careful decantation of the supernatant after each wash. The remaining solid residue was dried under reduced pressure. Yield of **12c** is 66 mg (96%) as a beige sticky oil. HPLC: gradient B 20/50% (30 min) t_R = 15.7 min, purity 97%. HRMS (ESI) calcd for $C_{22}H_{25}N_4O$, 361.2028 $[M+H]^+$; found, 361.1995. ^{1}H NMR (400 MHz, DMSO-d_6) δ 8.65 – 8.42 (m, 1H, CH), 8.18 (d, J = 7.8 Hz, 1H, CH), 7.80–7.66 (m, 1H, CH), 7.63–7.48 (m, 2H, CH), 7.41–7.24 (m, 4H, CH), 4.33 (t, J = 7.1 Hz, 2H, , CH_2), 3.65 (s, 2H, CH_2), 3.34 (s, 8H, CH_2), 2.20 (t, J = 8.1 Hz, 2H, CH_2). ^{13}C NMR (100 MHz, DMSO-d_6) δ, ppm 161.7, 159.3, 159.0, 147.8, 134.6, 134.1, 133.6, 130.2, 124.8, 124.2, 123.8, 123.6, 120.7, 116.2, 115.4, 115.4, 98.6, 53.8, 48.7, 40.9, 22.8, 22.7. HRMS (ESI) calcd for $C_{22}H_{25}N_4O$, 361.2023 $[M+H]^+$; found, 361.2013.

*6-(Pyrrolidin-1-ylmethyl)indolo[1,2-c]quinazoline (**14a**)*

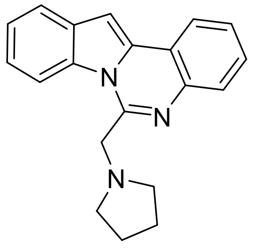

To a solution of compound **13** [3] (80 mg, 0.30 mmol) in toluene (3.0 mL), were added pyrrolidine (33 μL, 0.39 mmol) and potassium carbonate (0.15 g, 1.1 mmol). The reaction mixture was stirred at room temperature for 48 h. The reaction mixture was diluted with EtOAc (15 mL), washed with water (2 × 10 mL), dried over Na_2SO_4 and concentrated under reduced pressure. The residue was precipitated from DCM with petroleum ether (1:3). Yield of **14a** is 40 mg (44%) as a white solid, mp 103-105°C. HPLC: gradient B 30/70% (30 min) t_R = 13.3 min, purity 97%. ^{1}H NMR (400 MHz, DMSO-d_6) δ, ppm: 8.28-8.24 (m, 1H, CH), 8.13–8.10 (m, 1H, CH), 7.81 (dd, J = 6.1, 3.2 Hz, 1H, CH), 7.75–7.68 (m, 1H, CH), 7.62–7.50 (m, 2H, CH), 7.51 (s, 1H, CH), 7.47–

7.37 (m, 2H, CH), 4.24 (s, 2H, CH_2), 2.66 (d, J = 5.8 Hz, 4H, CH_2), 1.72–1.64 (m, 4H, CH_2). ^{13}C NMR (100 MHz, DMSO-d_6) δ, ppm: 149.0, 138.6, 134.8, 131.4, 130.1, 129.5, 127.9, 127.6, 123.7, 123.4, 122.5, 120.7, 120.6, 117.2, 96.4, 60.6, 53.8 (2C), 23.6 (2C). HRMS (ESI) calcd for $C_{20}H_{20}N_3$, 302.1652 $[M+H]^+$; found, 302.1661.

*6-(Piperidin-1-ylmethyl)indolo[1,2-c]quinazoline (**14b**)*

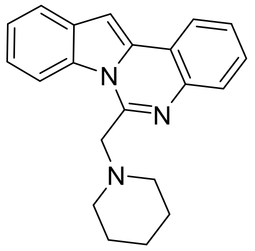

Compound **14b** was prepared from **13** as described for **14a**. Yield of **14b** is 62% (58 mg) as a white solid, mp 120-122°C. HPLC: gradient B 30/70% (30 min) t_R = 14.3 min, purity 98%. ^1H NMR (400 MHz, DMSO-d_6) δ 8.24 (dd, J = 6.9, 2.2 Hz, 1H, CH), 8.12–8.04 (m, 1H, CH), 7.85–7.77 (m, 1H, CH), 7.70 (dd, J = 7.0, 2.0 Hz, 1H, CH), 7.61–7.48 (m, 3H, CH), 7.43–7.34 (m, 2H, CH), 4.05 (s, 2H, CH_2), 2.55 (s, 4H, CH_2), 1.48–1.33 (m, 6H, CH_2). ^{13}C NMR (100 MHz, DMSO-d_6) δ, ppm: 148.0, 138.4, 134.8, 131.4, 130.0, 129.5, 128.0, 127.6, 123.7, 123.4, 122.4, 120.6, 120.5, 117.3, 96.5, 63.5, 54.3 (2C), 25.9 (2C), 24.2. HRMS (ESI) calcd for $C_{21}H_{22}N_3$, 316.1808 $[M+H]^+$; found, 316.1816.

*4-(Indolo[1,2-c]quinazolin-6-ylmethyl)morpholine (**14c**)*

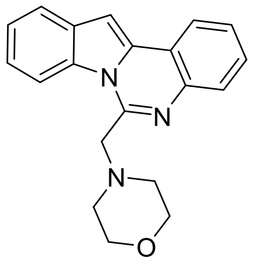

Compound **14c** was prepared from **13** as described for **14a**. Yield of **14c** is 69 mg (73%) as a brown solid, mp 150-154°C (decomp.). HPLC: gradient B 30/70% (30 min) t_R = 11.5 min, purity 96%. ^1H NMR (400 MHz, DMSO-d_6) δ, ppm: 8.25–8.22 (m, 1H, CH), 8.10–8.07 (m, 1H, CH),

7.85–7.78 (m, 1H, CH), 7.75–7.67 (m, 1H, CH), 7.60–7.49 (m, 3H, CH), 7.42–7.37 (m, 2H, CH), 4.13 (s, 2H, CH_2), 3.52 (t, J = 4.6 Hz, 4H, CH_2), 2.61 (t, J = 4.4 Hz, 4H, CH_2). ^{13}C NMR (100 MHz, DMSO-d_6) δ, ppm: 147.4, 138.3, 134.8, 131.3, 130.1, 129.6, 128.1, 127.6, 123.8, 123.4, 122.5, 120.7, 120.6, 117.2, 96.5, 66.5 (2C), 63.0, 53.6 (2C). HRMS (ESI) calcd for $C_{20}H_{18}N_3O$, 316.1455 [M+H]$^+$; found, 316.1439.

*6-((4-Methylpiperazin-1-yl)methyl)indolo[1,2-c]quinazoline (**14d**)*

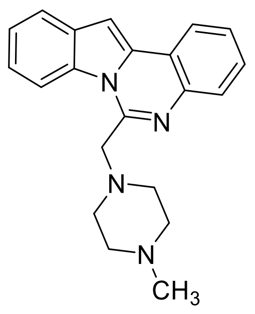

Compound **14d** was prepared from **13** as described for **14a**. Yield of **14d** is 70 mg (71%) as a brown solid. HPLC: gradient B 30/70% (30 min)) t_R = 9.5 min, purity 98%. ^{1}H NMR (400 MHz, DMSO-d_6) δ 8.28–8.20 (m, 1H, CH), 8.10–8.04 (m, 1H, CH), 7.84–7.77 (m, 1H, CH), 7.76–7.67 (m, 1H, CH), 7.60–7.49 (m, 3H, CH), 7.42–7.33 (m, 2H, CH), 4.10 (s, 2H, CH_2), 2.60 (s, 4H, CH_2), 2.25 (s, 4H, CH_2), 2.08 (s, 3H, CH_3). ^{13}C NMR (100 MHz, DMSO-d_6) δ, ppm: 147.8, 138.4, 134.8, 131.3, 130.1, 129.5, 128.1, 127.6, 123.8, 123.4, 122.4, 120.7, 120.5, 117.3, 96.5, 62.8, 54.9 (2C), 53.0 (2C), 46.0. HRMS (ESI) calcd for $C_{21}H_{23}N_4$, 331.1917 [M+H]$^+$; found, 331.1922.

Cell lines, drug treatment and cytotoxicity assays

In a manner similar to [4] all tumor cell lines were from American Type Culture Collection (Manassas, VA). Non-malignant hFB-hTERT6 human skin fibroblasts (HSF) were obtained via a lentiviral transduction of full-length TERT gene under a cytomegalovirus promoter (generated at Engelhardt Institute of Molecular Biology, Moscow by E. Dashinimaev). Cells were cultured in Dulbecco's modified eagle medium (DMEM, PanEco, Russia) (adherent lines) or RPMI-1640 medium (PanEco, Russia) (suspension lines), supplemented with 5% fetal calf serum (HyClone,

Logan, UT), 2 mM L-glutamine, 100 U/mL penicillin, and 100 µg/mL streptomycin at 37 °C, 5% CO_2 in a humidified atmosphere. Cells in logarithmic growth phase were used in all experiments. Compounds were dissolved in 10% aqueous DMSO as 10 mM stock solutions followed by serial dilutions in water immediately before experiments. Briefly, cells (5×10^3 in 190 mL of culture medium) were plated into a 96-well plate (Becton Dickinson, Franklin Lakes, NJ) and treated with 0.1% DMSO (vehicle control) or with increasing concentrations of compounds for 72 h. After the completion of drug exposure, 0.5 mg/mL of 3-(4,5-dimethylthiazol-2-yl)-2,5-diphenyltetrazolium bromide was added into each well for an additional 2 h. Formazan was dissolved in DMSO, and the absorbance at 570 nm was measured. The cytotoxicity at a given drug concentration was calculated as the percentage of absorbance in wells with drug-treated cells to that of vehicle control cells (100%). The IC_{50} (50% growth inhibitory concentration) was defined as the concentration of the compound that inhibited MTT conversion by 50%.

DNA binding assay by fluorescence titration

In a manner similar to [5] formation of complexes of compounds with calf thymus DNA (Sigma Aldrich) was quantitatively assessed by fluorescence spectroscopy. Drug–DNA binding was determined in 100 mM KCl, 20 mM tris-HCl buffer pH 8.0 at 25 °C. Two µM of compounds were incubated with ctDNA aliquots (5 min) after each DNA addition. Fluorescence spectra were recorded with a Cary Eclipse Fluorescence Spectrometer (Australia). The excitation wavelength was 340 nm (slits 5 nm). Emission was recorded in range 350–600 nm. The concentration of ctDNA was determined in a sodium phosphate buffered solution at 20 °C using the molar extinction coefficient ε[ctDNA] = 13200 M (base pairs)$^{-1}$·cm^{-1}. The concentration of free and bound compounds was determined by changes of ligand fluorescence upon DNA titration. Binding parameters were determined by approximation of DNA concentration dependence of fluorescence with equation $F/F_0 = 1/1 + C/K_D$.

DNA binding estimation by FID assay

To study ligand binding, we employed a DNA double helix model using the self-complementary oligonucleotide ds26, d(CAATCGGATCGAATTCGATCCGATTG), which forms a 26-base-pair duplex. DNA–ligand interactions were screened using the thiazole orange (TO) displacement assay (FID method) [7]. This assay is based on the competitive binding of the test ligand and TO, a known fluorescent intercalator, to DNA. TO fluorescence was recorded at 530 nm (emission slit width 20 nm) with excitation at 480 nm (excitation slit width 10 nm) using a TECAN Spark microplate fluorometer at room temperature. Each test sample contained DNA (0.1 μM), TO (1 μM), and compound (25 μM). Data represent the average of three independent measurements.

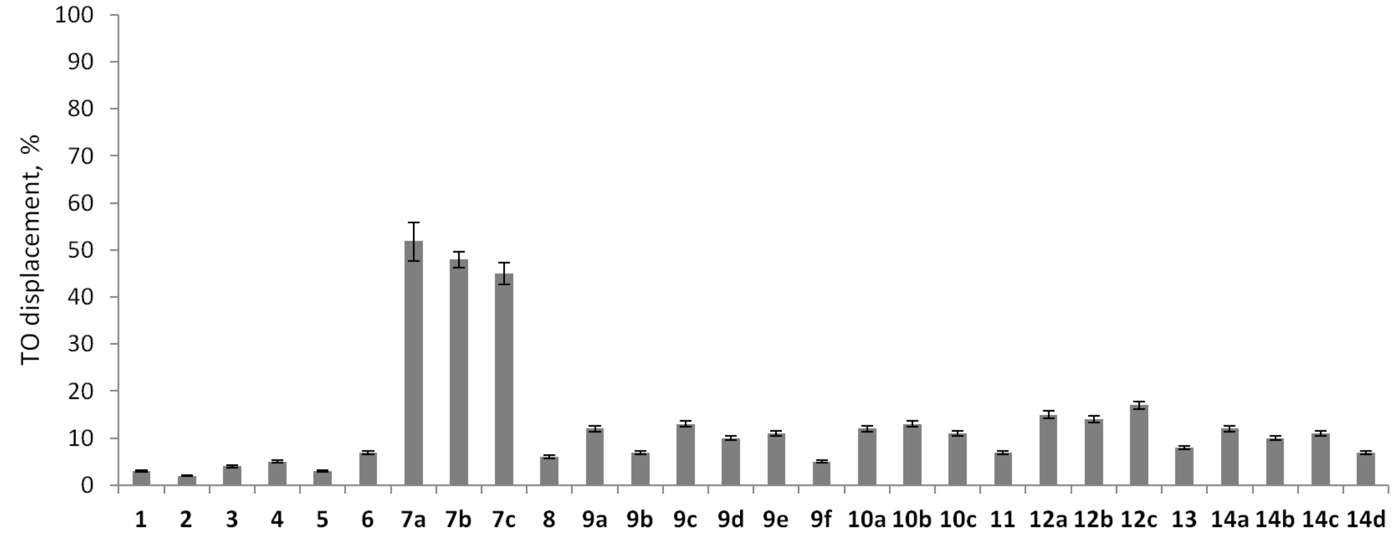

Figure S1. Thiazole orange (TO) displacement from DNA duplex (ds26) by the compounds at 25 µM at 20 °C.

A

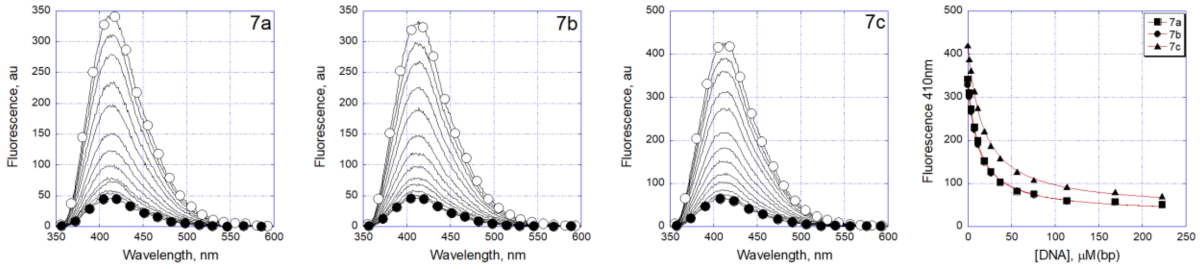

B

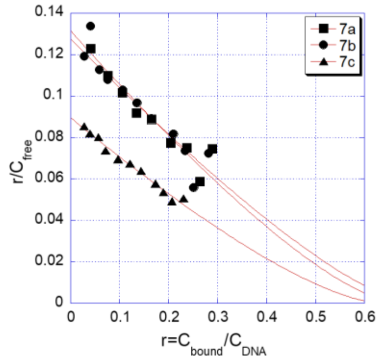

Compound	K_a, 10^5 M^{-1}	K_D, µM	L, bp
**7a**	1.2±0.1	8.3±0.7	1.4±0.1
**7b**	1.3±0.1	7.7±0.6	1.5±0.1
**7c**	0.9±0.1	11.1±1.2	1.57±0.05

Figure S2. (A) Fluorescence titration of compounds **7a–c** (5 µM) with calf thymus DNA at low ionic strength. Spectra were recorded upon addition of DNA (0–220 µM base pairs) in 20 mM Tris-HCl buffer (pH 8.0) at 20 °C. (B) Scatchard plot of the binding isotherm derived from the fluorescence titration data. The table presents the binding parameters obtained by approximation with the McGhee–von Hippel equation [6].

References:

1. Bergman, J.; Carlsson, R.; Sjöberg, B. *J. Heterocycl. Chem.* **1977**, 14, 1123-1134. DOI: 10.1002/jhet.5570140701.

2. Xu, M.; Hou, Q.; Wang, S.; Wang, H.; Yao, Z.-J. *Synthesis* **2011**, 4, 626-634. DOI: 10.1055/s-0030-1258411.

3. Duncan, R. L.; Helsley, G. C.; Boswell, R. F. *J. Heterocycl. Chem.* **1973**, 10, 65-70. DOI: 10.1002/jhet.5570100115.

4. Tikhomirov, A. S.; Shchekotikhin, A. E.; Lee, Y. H.; Chen, Y. A.; Yeh, C. A.; Tatarskiy, V. V.; Dezhenkova, L. G.; Glazunova, V. A.; Balzarini, J.; Shtil, A. A.; Preobrazhenskaya, M. N.; Chueh, P. J. *J. Med. Chem.* **2015**, 58, 9522-9534. DOI: 10.1021/acs.jmedchem.5b00859.

5. Tikhomirov, A.S.; Sinkevich, Y.B.; Dezhenkova, L.G.; Kaluzhny, D.N.; Ilyinsky, N.S.; Borshchevskiy, V.I.; Schols, D.; Shchekotikhin, A.E. *Eur. J. Med. Chem.* **2024**, *265*, 116103, https://doi.org/10.1016/j.ejmech.2023.116103.

6. McGhee J. D., von Hippel P. H. *J. Mol. Biol.* **1974**, *86*, 469-489.

7. Monchaud, D.; Teulade-Fichou, M.-P. *Methods Mol. Biol.* **2010**, *608*, 257, https://doi.org/10.1007/978-1-59745-363-9_15

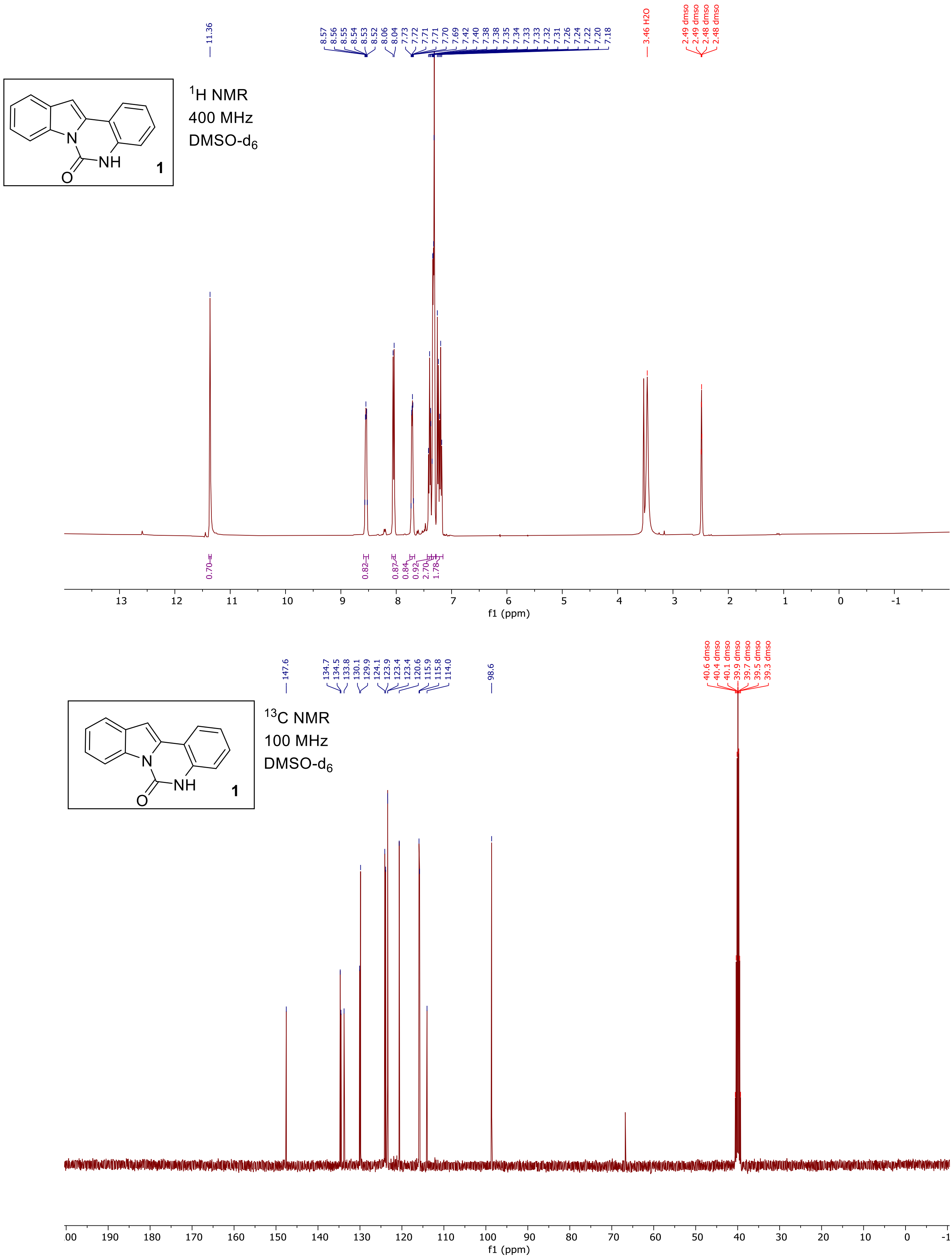

Figure S3. ^{1}H and ^{13}C NMR spectra of compound **1**.

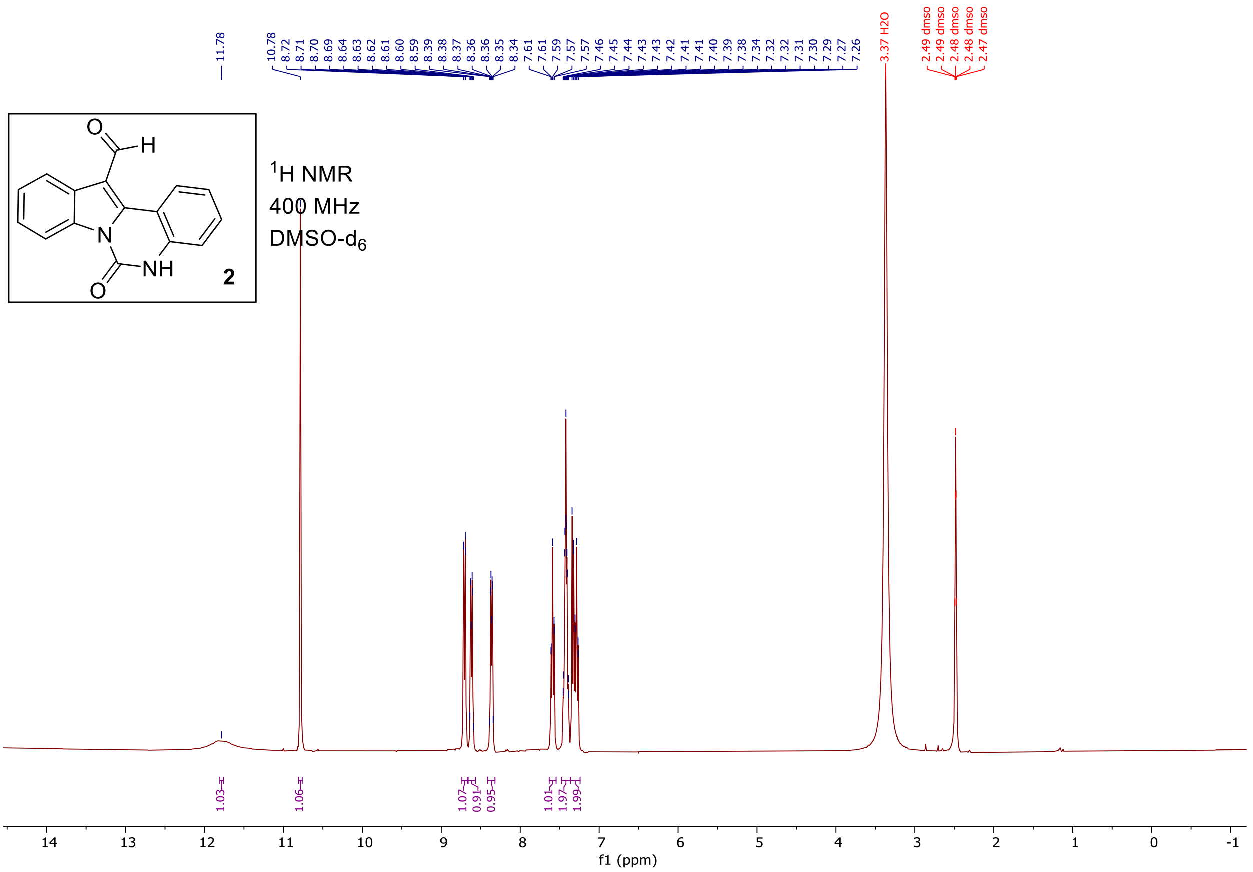

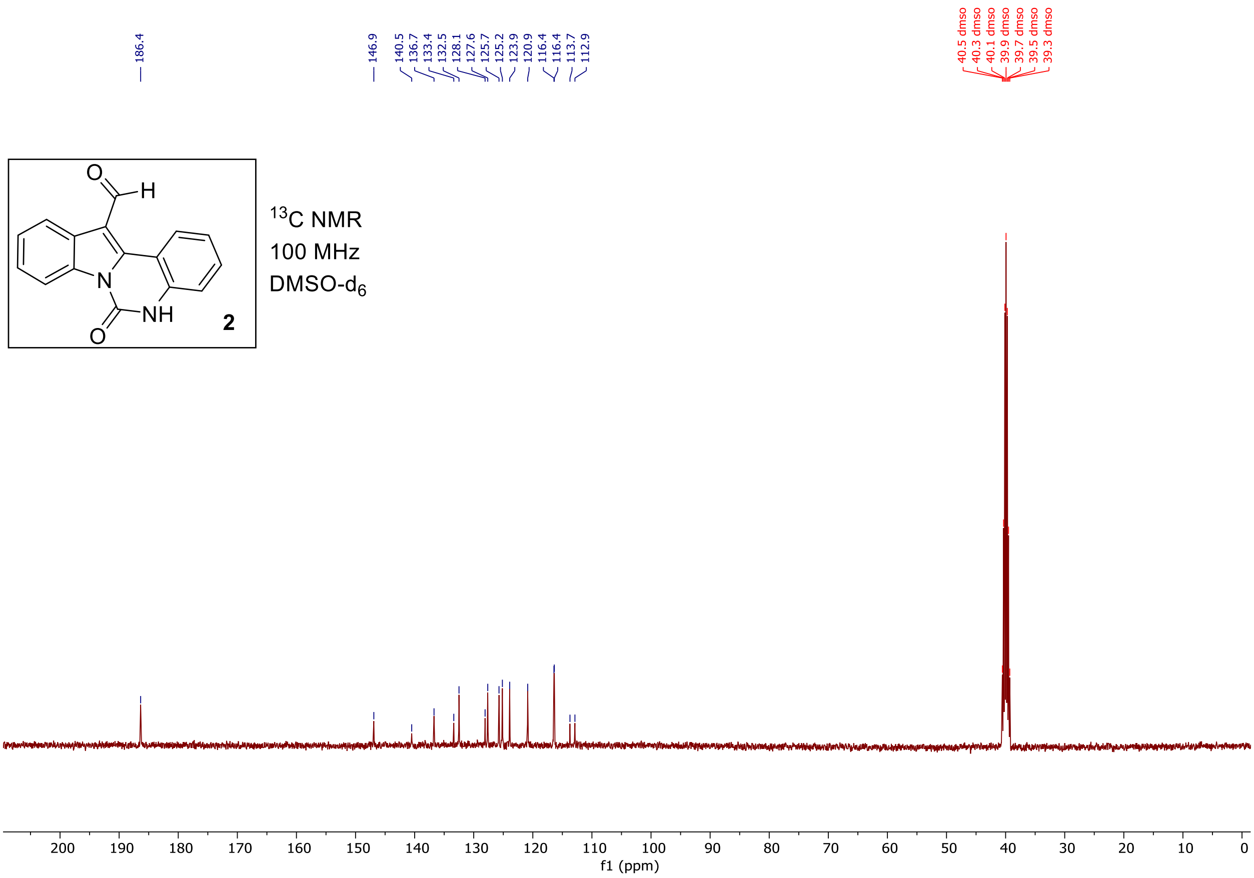

Figure S4. ^{1}H and ^{13}C NMR spectra of compound **2**.

Compound Spectrum List Report

Analysis Info

Analysis Name	D:\Data\AST\KDV-18.d	Acquisition Date	6/26/2025
Method	tune_low_norm.m	Operator	Mitrokhov
Sample Name	Tune wide	Instrument / Ser#	micrOTOF-Q II 10225
Comment			

Acquisition Parameter

Source Type	ESI	Ion Polarity	Positive	Set Nebulizer	0.4 Bar
Focus	Active	Set Capillary	4500 V	Set Dry Heater	180 °C
Scan Begin	50 m/z	Set End Plate Offset	-500 V	Set Dry Gas	4.0 l/min
Scan End	3000 m/z	Set Collision Cell RF	150.0 Vpp	Set Divert Valve	Source

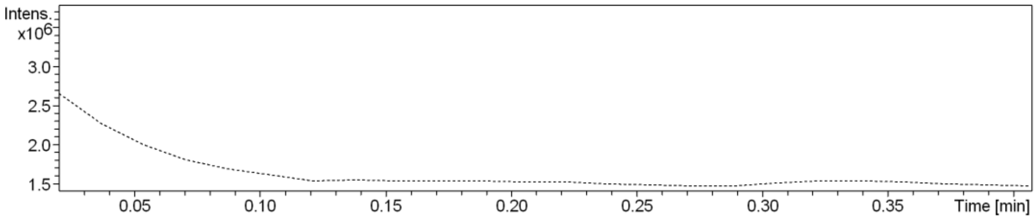

#	RT [min]	Area	Max. m/z
n.a.	0.2	n.a.	263.0829

+MS, 0.1-0.3min #(5-19)

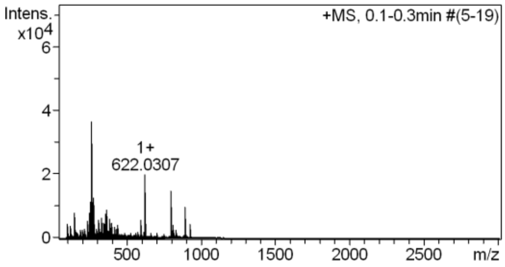

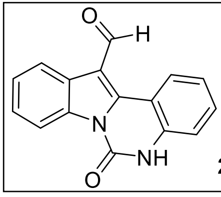

2

#	m/z	I	I %
1	149.0273	7929	21.7
2	249.0696	7948	21.7
3	258.1761	11416	31.2
4	263.0829	36572	100.0
5	264.0929	8192	22.4
6	279.0803	12734	34.8
7	361.2233	8727	23.9
8	622.0307	19978	54.6
9	796.4235	14894	40.7
10	891.5323	9711	26.6

Bruker Compass DataAnalysis 4.0 printed: 6/26/2025 9:50:03 AM Page 1 of 1

Figure S5. Copy of HRMS (ESI) spectra of compound **2**.

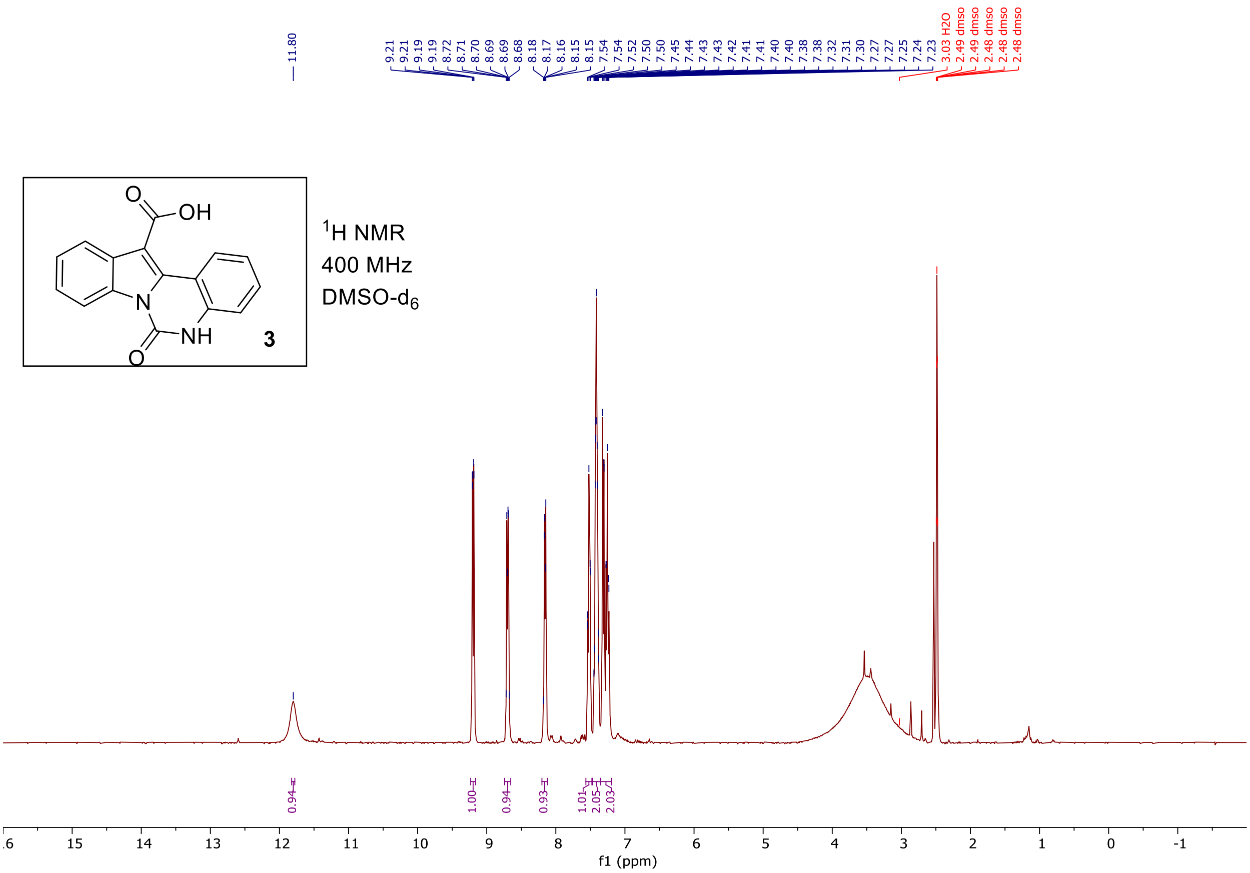

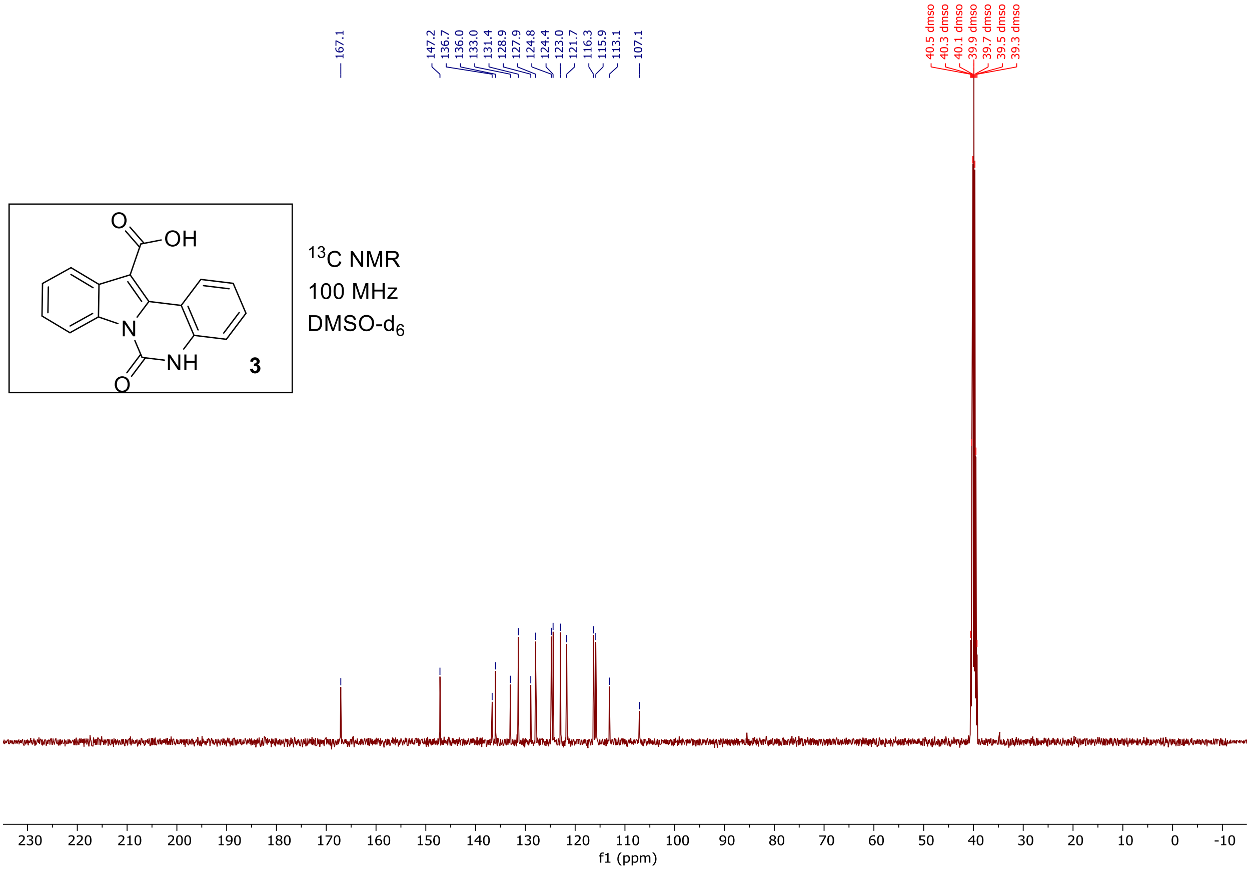

Figure S6. ^{1}H and ^{13}C NMR spectra of compound **3**.

Compound Spectrum List Report

Analysis Info

Analysis Name	D:\Data\AST\KDV-22.d	Acquisition Date	6/27/2025
Method	tune_low_norm.m	Operator	Mitrokhov
Sample Name	Tune wide	Instrument / Ser#	micrOTOF-Q II 10225
Comment			

Acquisition Parameter

Source Type	ESI	Ion Polarity	Positive	Set Nebulizer	0.4 Bar
Focus	Active	Set Capillary	4500 V	Set Dry Heater	180 °C
Scan Begin	50 m/z	Set End Plate Offset	-500 V	Set Dry Gas	4.0 l/min
Scan End	3000 m/z	Set Collision Cell RF	150.0 Vpp	Set Divert Valve	Source

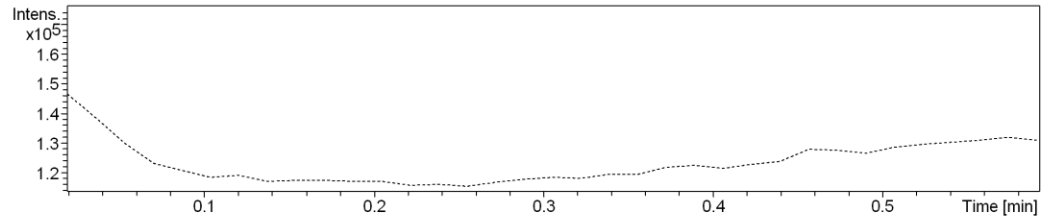

#	RT [min]	Area	Max. m/z
n.a.	0.2	n.a.	235.0900

+MS, 0.1-0.3min #(4-15)

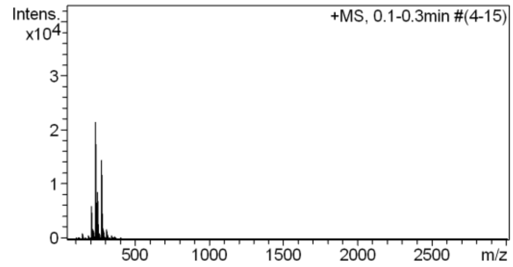

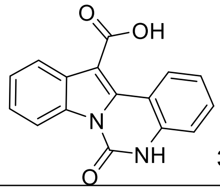

3

#	m/z	I	I %
1	209.1104	5891	27.5
2	219.0936	1435	6.7
3	223.0896	1425	6.6
4	235.0900	21459	100.0
5	236.0925	3918	18.3
6	249.0695	8470	39.5
7	279.0780	14479	67.5
8	280.0806	2397	11.2
9	287.0804	1564	7.3
10	313.2745	1677	7.8

Bruker Compass DataAnalysis 4.0 printed: 6/27/2025 9:52:16 AM Page 1 of 1

Figure S7. Copy of HRMS (ESI) spectra of compound **3**.

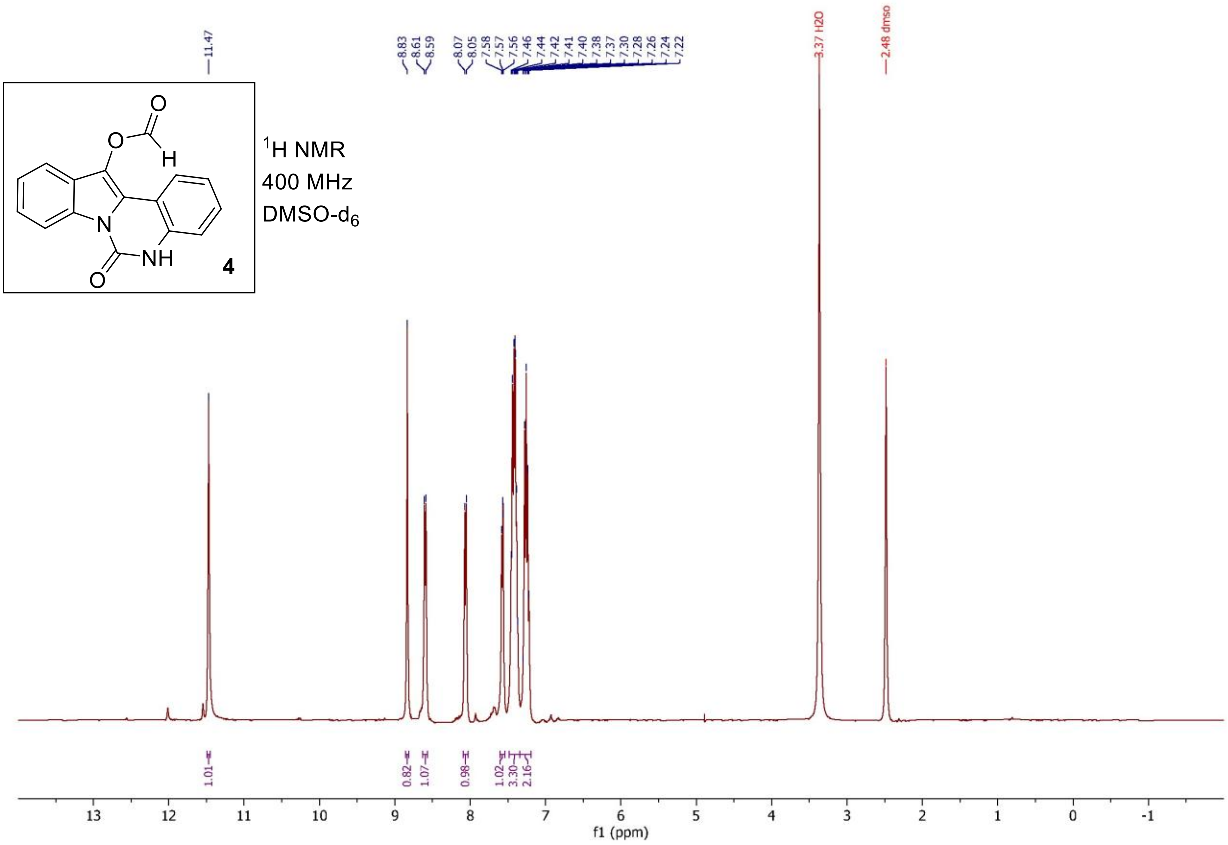

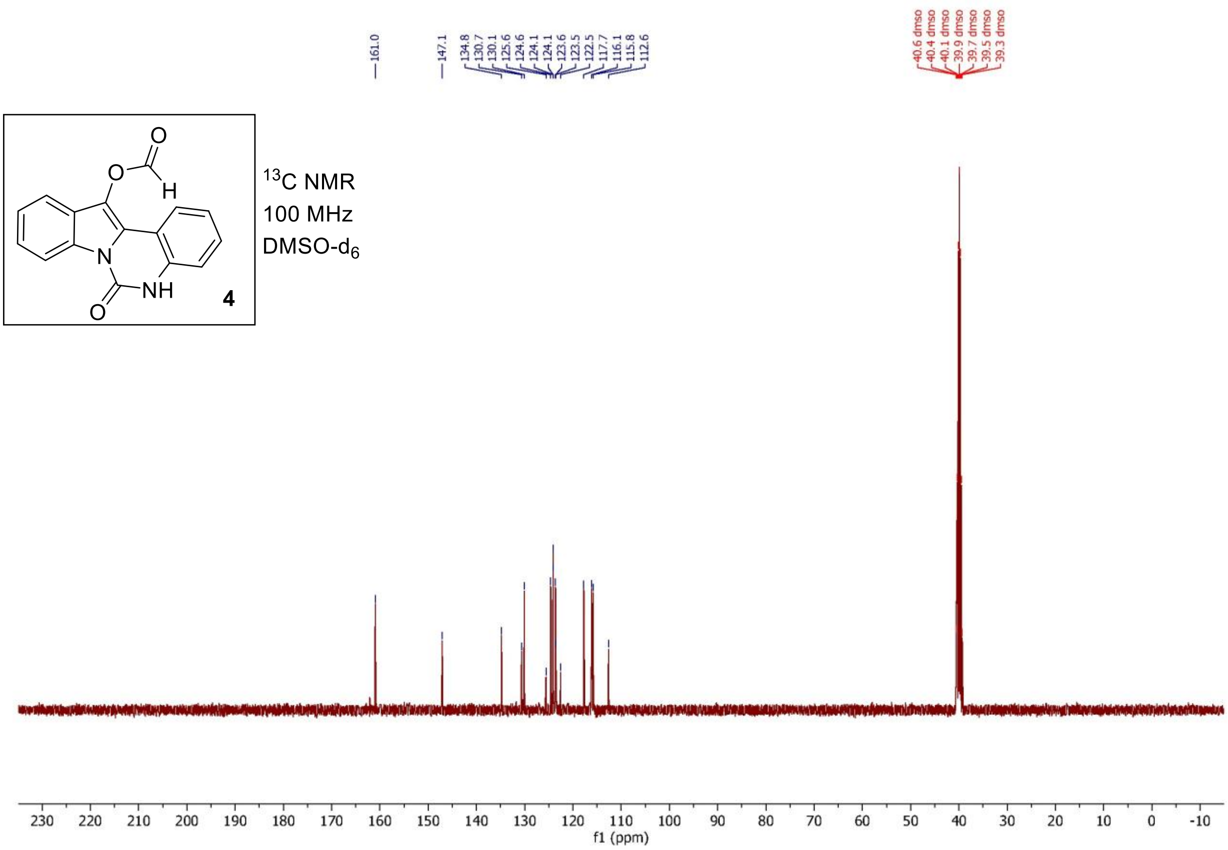

Figure S8. ^{1}H and ^{13}C NMR spectra of compound **4**.

Compound Spectrum List Report

Analysis Info

Analysis Name	D:\Data\AST\KDV-20.d	Acquisition Date	6/26/2025
Method	tune_low_norm.m	Operator	Mitrokhov
Sample Name	Tune wide	Instrument / Ser#	micrOTOF-Q II 10225
Comment			

Acquisition Parameter

Source Type	ESI	Ion Polarity	Positive	Set Nebulizer	0.4 Bar
Focus	Active	Set Capillary	4500 V	Set Dry Heater	180 °C
Scan Begin	50 m/z	Set End Plate Offset	-500 V	Set Dry Gas	4.0 l/min
Scan End	3000 m/z	Set Collision Cell RF	150.0 Vpp	Set Divert Valve	Source

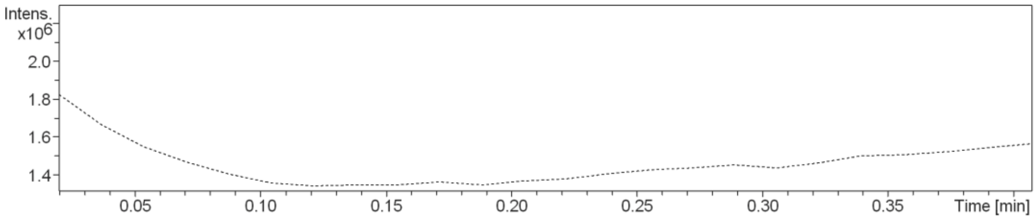

#	RT [min]	Area	Max. m/z
n.a.	0.2	n.a.	279.0772

+MS, 0.1-0.3min #(4-18)

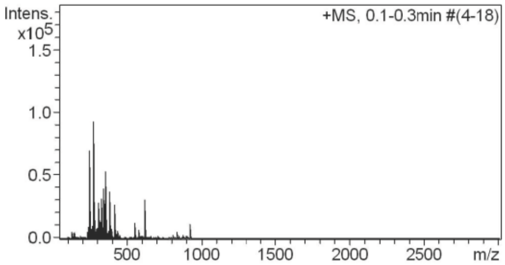

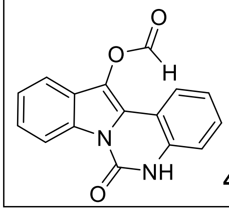

4

#	m/z	I	I %
1	249.0704	69922	75.2
2	279.0772	92937	100.0
3	313.2764	27925	30.0
4	331.2872	31412	33.8
5	341.3079	39551	42.6
6	353.2683	27170	29.2
7	359.3188	53057	57.1
8	381.3004	36821	39.6
9	418.2895	26372	28.4
10	622.0339	30171	32.5

Bruker Compass DataAnalysis 4.0 printed: 6/26/2025 9:50:39 AM Page 1 of 1

Figure S9. Copy of HRMS (ESI) spectra of compound **4**.

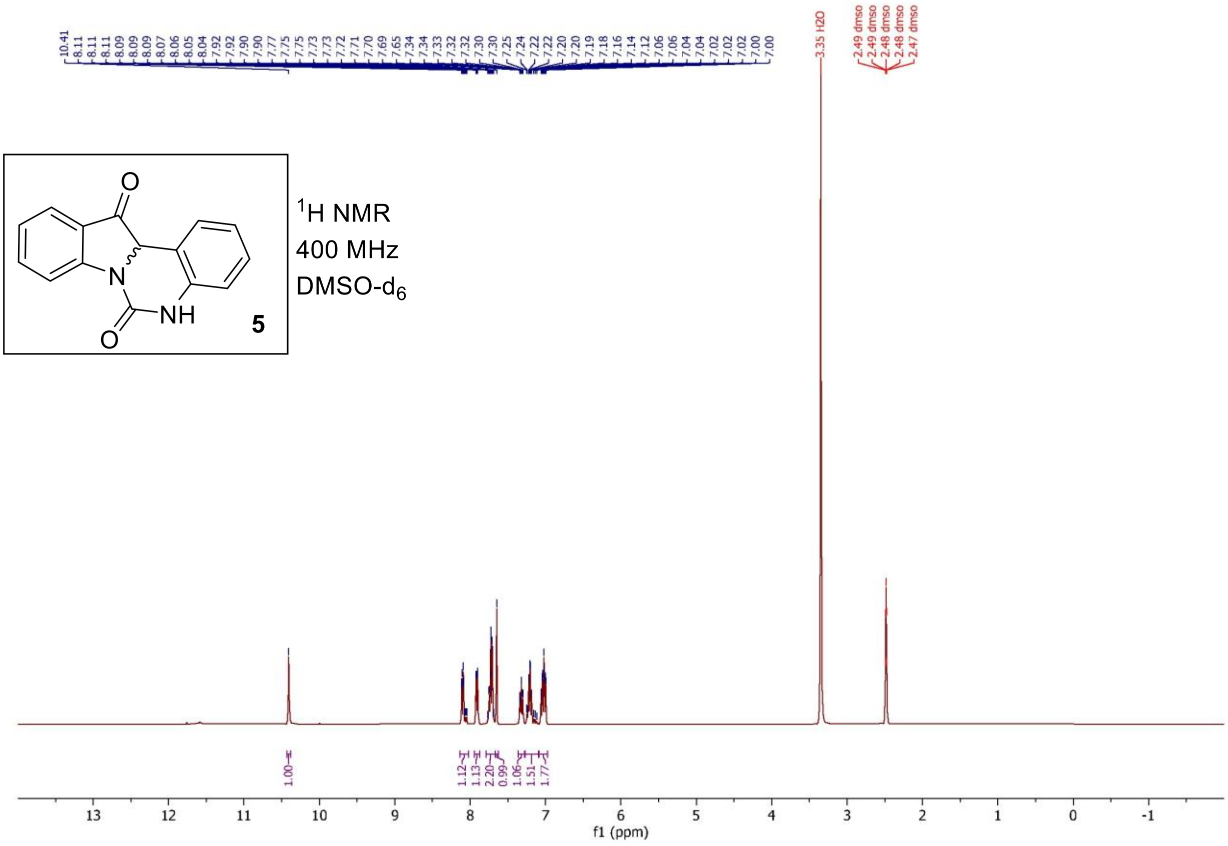

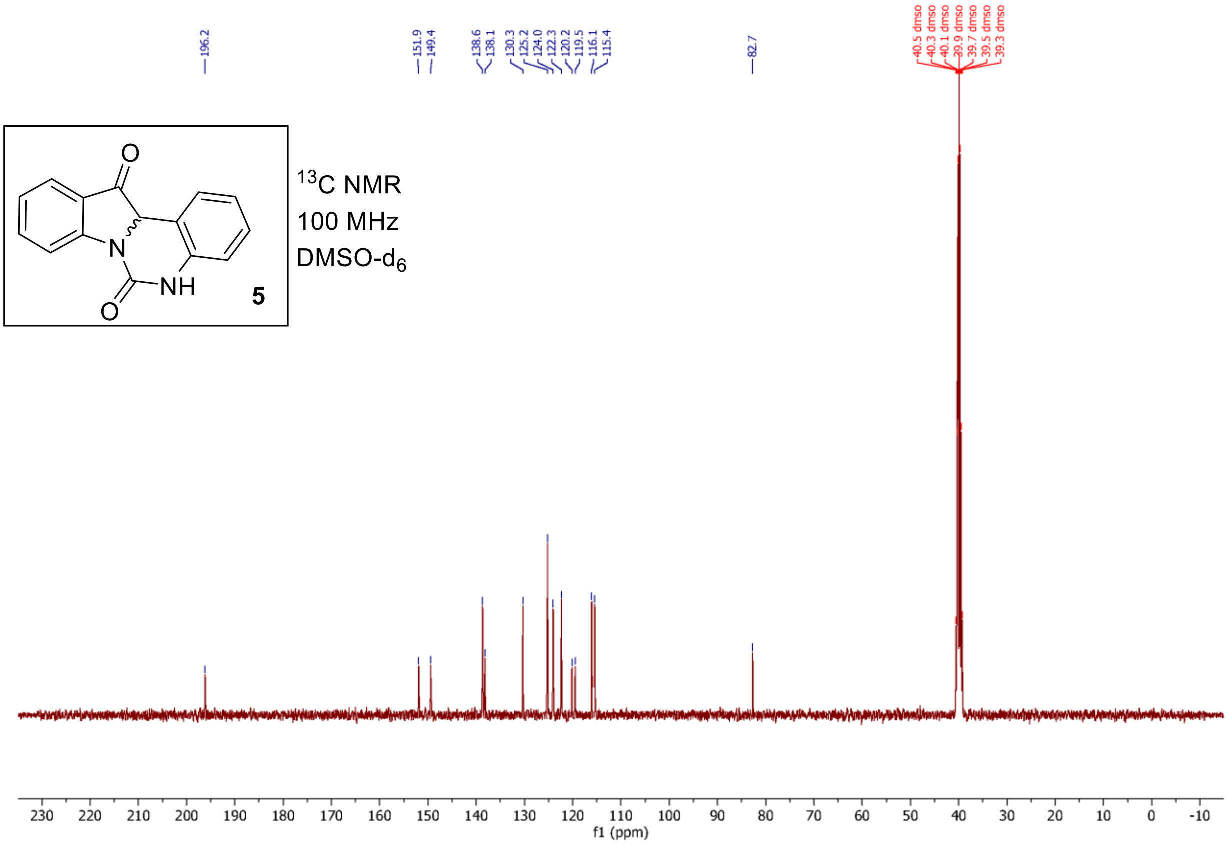

Figure S10. ^{1}H and ^{13}C NMR spectra of compound **5**.

Compound Spectrum List Report

Analysis Info

Analysis Name	D:\Data\AST\AST-739.d	Acquisition Date	6/28/2025
Method	tune_low_norm.m	Operator	Mitrokhov
Sample Name	Tune wide	Instrument / Ser#	micrOTOF-Q II 10225
Comment			

Acquisition Parameter

Source Type	ESI	Ion Polarity	Positive	Set Nebulizer	0.4 Bar
Focus	Active	Set Capillary	4500 V	Set Dry Heater	180 °C
Scan Begin	50 m/z	Set End Plate Offset	-500 V	Set Dry Gas	4.0 l/min
Scan End	3000 m/z	Set Collision Cell RF	150.0 Vpp	Set Divert Valve	Source

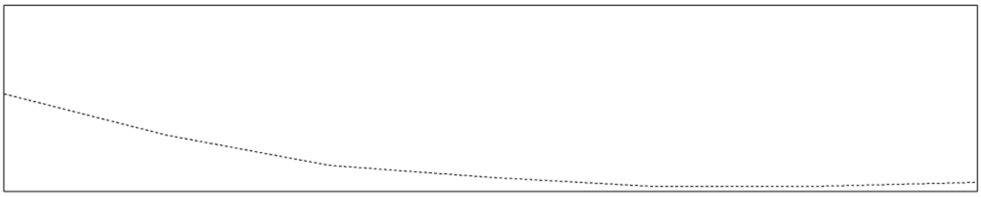

#	RT [min]	Area	Max. m/z
n.a.	0.1	n.a.	251.0802

+MS, 0.0-0.1min #(2-6)

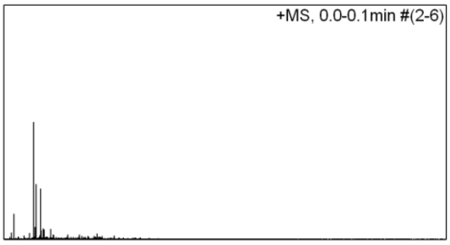

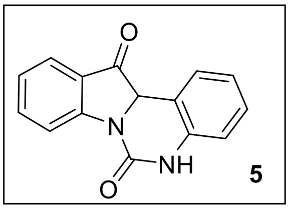

#	m/z	I	I %
1	118.0880	228336	21.3
2	251.0802	1073124	100.0
3	255.0710	161475	15.0
4	258.1750	108158	10.1
5	265.0638	497397	46.4
6	297.0900	457848	42.7
7	298.0926	76043	7.1
8	314.1151	93748	8.7
9	319.0687	85038	7.9
10	361.2224	86599	8.1

Bruker Compass DataAnalysis 4.0 printed: 6/28/2025 1:28:42 PM Page 1 of 1

Figure S11. Copy of HRMS (ESI) spectra of compound **5**.

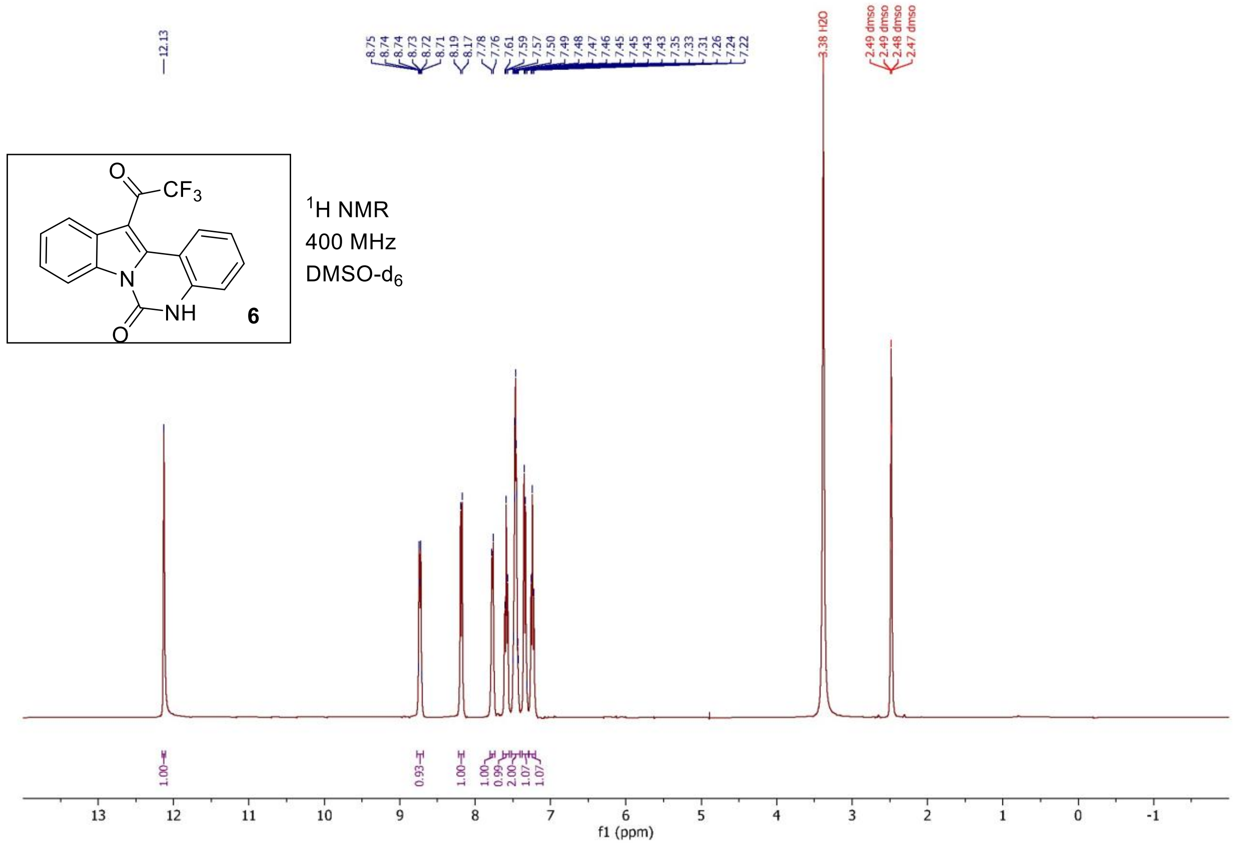

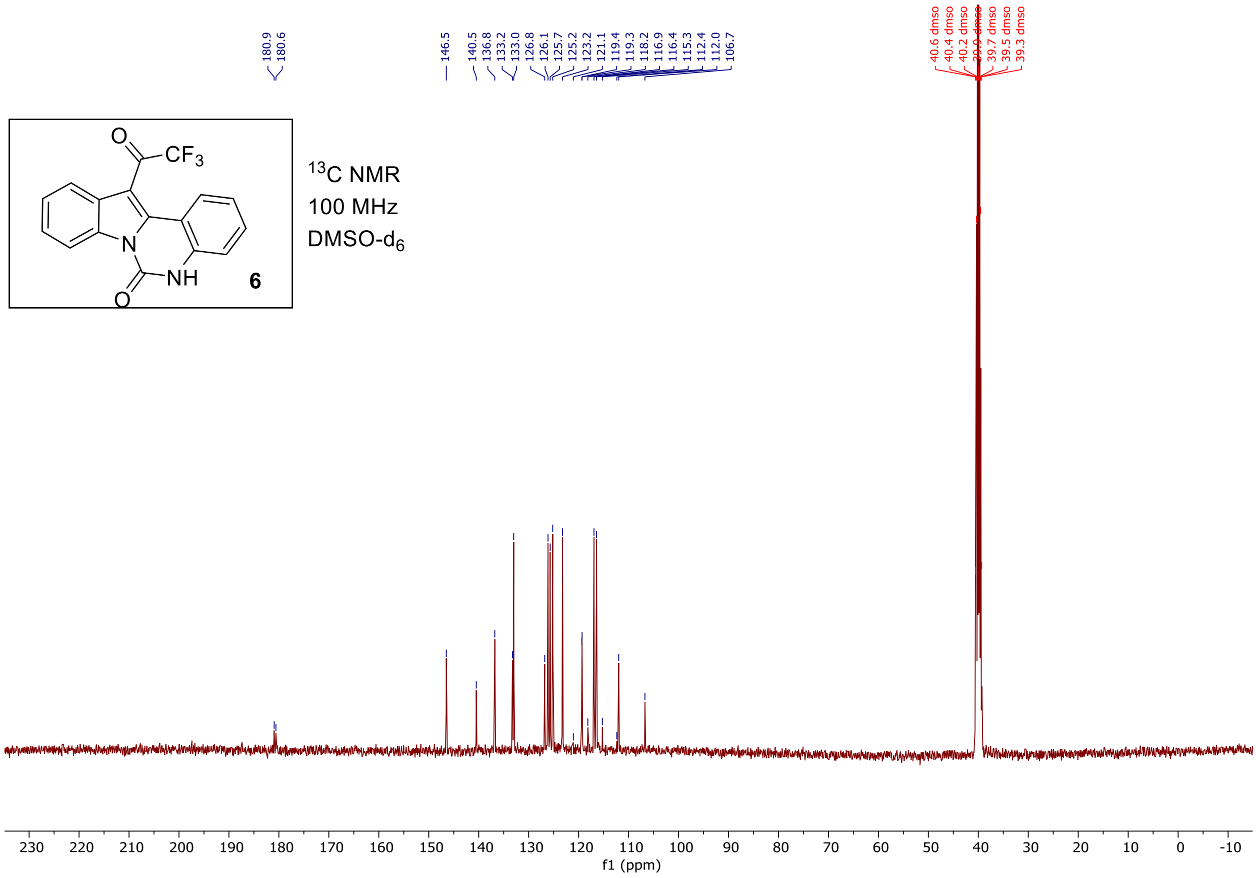

Figure S12. ^{1}H and ^{13}C NMR spectra of compound **6**.

Compound Spectrum List Report

Analysis Info

Analysis Name	D:\Data\AST\KDV-21.d	Acquisition Date	6/29/2025
Method	tune_norm.m	Operator	Mitrokhov
Sample Name	Tune wide	Instrument / Ser#	micrOTOF-Q II 10225
Comment			

Acquisition Parameter

Source Type	ESI	Ion Polarity	Positive	Set Nebulizer	0.4 Bar
Focus	Active	Set Capillary	4500 V	Set Dry Heater	180 °C
Scan Begin	50 m/z	Set End Plate Offset	-500 V	Set Dry Gas	4.0 l/min
Scan End	3000 m/z	Set Collision Cell RF	550.0 Vpp	Set Divert Valve	Source

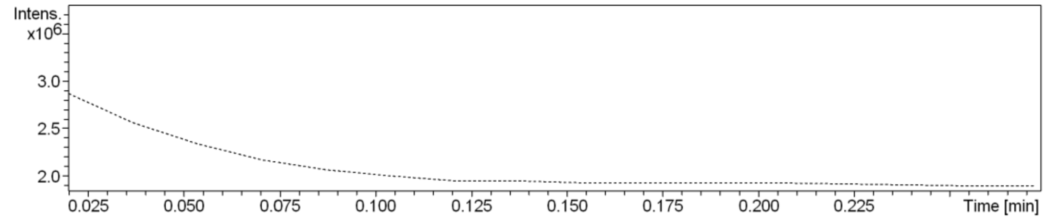

#	RT [min]	Area	Int. Type	Intens.	S/N	Chromatogram	Max. m/z
n.a.	0.2	n.a.	Average spectrum	n.a.	n.a.	n.a.	341.3058

+MS, 0.1-0.3min #(3-16)

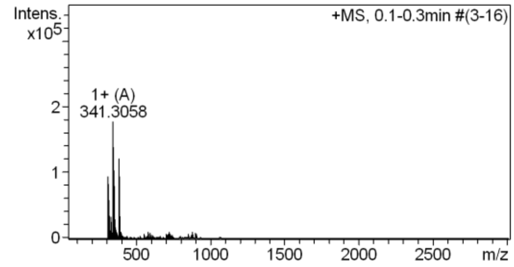

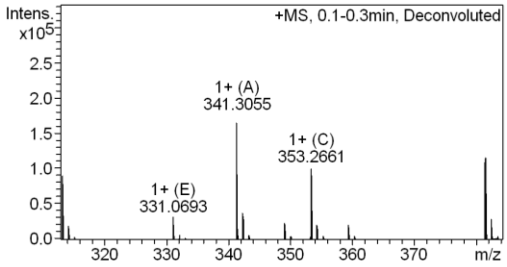

#	m/z	Res.	S/N	I	I %
1	313.2742	10036	1371.0	93304	52.6
2	331.0693	9372	438.4	32860	18.5
3	341.3058	10487	2253.9	177522	100.0
4	342.3080	8534	478.7	37776	21.3
5	349.0795	9380	292.6	23372	13.2
6	353.2657	9722	1281.7	103068	58.1
7	354.2689	8632	258.0	20795	11.7
8	359.3142	9047	257.9	20964	11.8
9	381.2968	10194	1431.9	120676	68.0
10	382.2993	9475	343.2	28988	16.3

#	m/z	Res.	S/N	I	I %
1	313.2740			93303	52.6
2	331.0693			32860	18.5
3	341.3055			177522	100.0
4	349.0793			23371	13.2
5	353.2661			103067	58.1
6	359.3141			20964	11.8
7	381.2965			120676	68.0

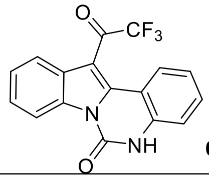

6

Figure S13. Copy of HRMS (ESI) spectra of compound **6**.

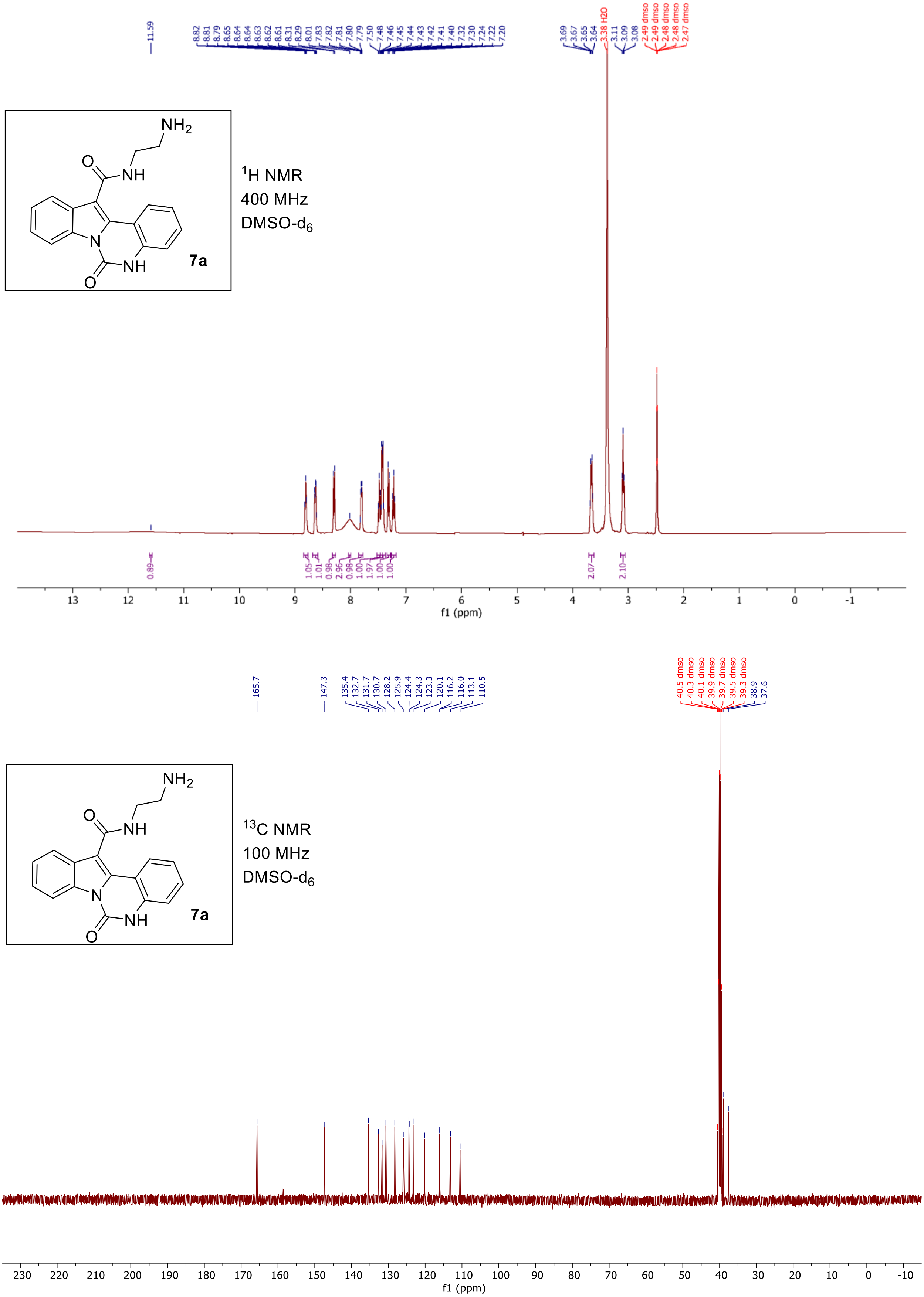

Figure S14. ^{1}H and ^{13}C NMR spectra of compound **7a**.

Compound Spectrum List Report

Analysis Info

Analysis Name	D:\Data\AST\KDV-24().d	Acquisition Date	6/26/2025
Method	tune_norm.m	Operator	Mitrokhov
Sample Name	Tune wide	Instrument / Ser#	micrOTOF-Q II 10225
Comment			

Acquisition Parameter

Source Type	ESI	Ion Polarity	Positive	Set Nebulizer	0.4 Bar
Focus	Active	Set Capillary	4500 V	Set Dry Heater	180 °C
Scan Begin	50 m/z	Set End Plate Offset	-500 V	Set Dry Gas	4.0 l/min
Scan End	3000 m/z	Set Collision Cell RF	550.0 Vpp	Set Divert Valve	Source

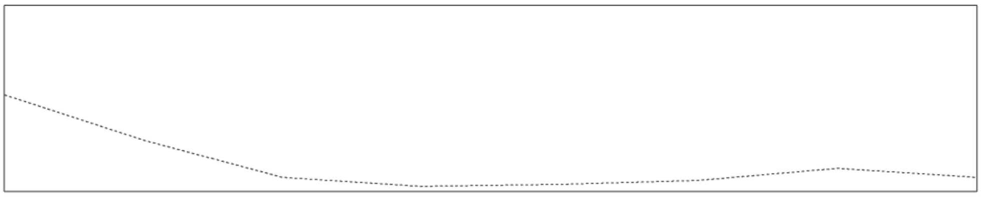

#	RT [min]	Area	Int. Type	Intens.	S/N	Chromatogram	Max. m/z
n.a.	0.1	n.a.	Average spectrum	n.a.	n.a.	n.a.	321.1335

+MS, 0.1-0.1min #(3-8)

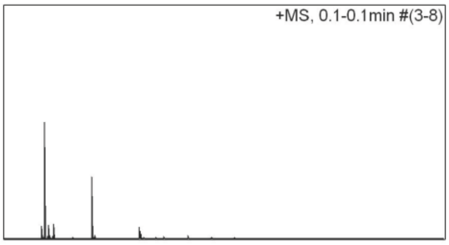

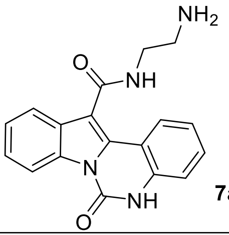

7a

#	m/z	Res.	S/N	I	I %
1	304.1007	10626	1800.0	233856	10.0
2	321.1335	13837	16395.4	2329899	100.0
3	322.1317	12873	4740.1	677026	29.1
4	353.2572	11195	1771.8	271918	11.7
5	381.2880	11510	1855.1	273180	11.7
6	641.2544	15435	7645.6	1226926	52.7
7	642.2547	14082	3156.2	508005	21.8
8	643.2508	10479	626.6	101190	4.3
9	961.3723	13566	1367.3	213308	9.2
10	962.3726	12280	899.8	140554	6.0

Bruker Compass DataAnalysis 4.0 printed: 6/28/2025 6:18:24 PM Page 1 of 1

Figure S15. Copy of HRMS (ESI) spectra of compound **7a**.

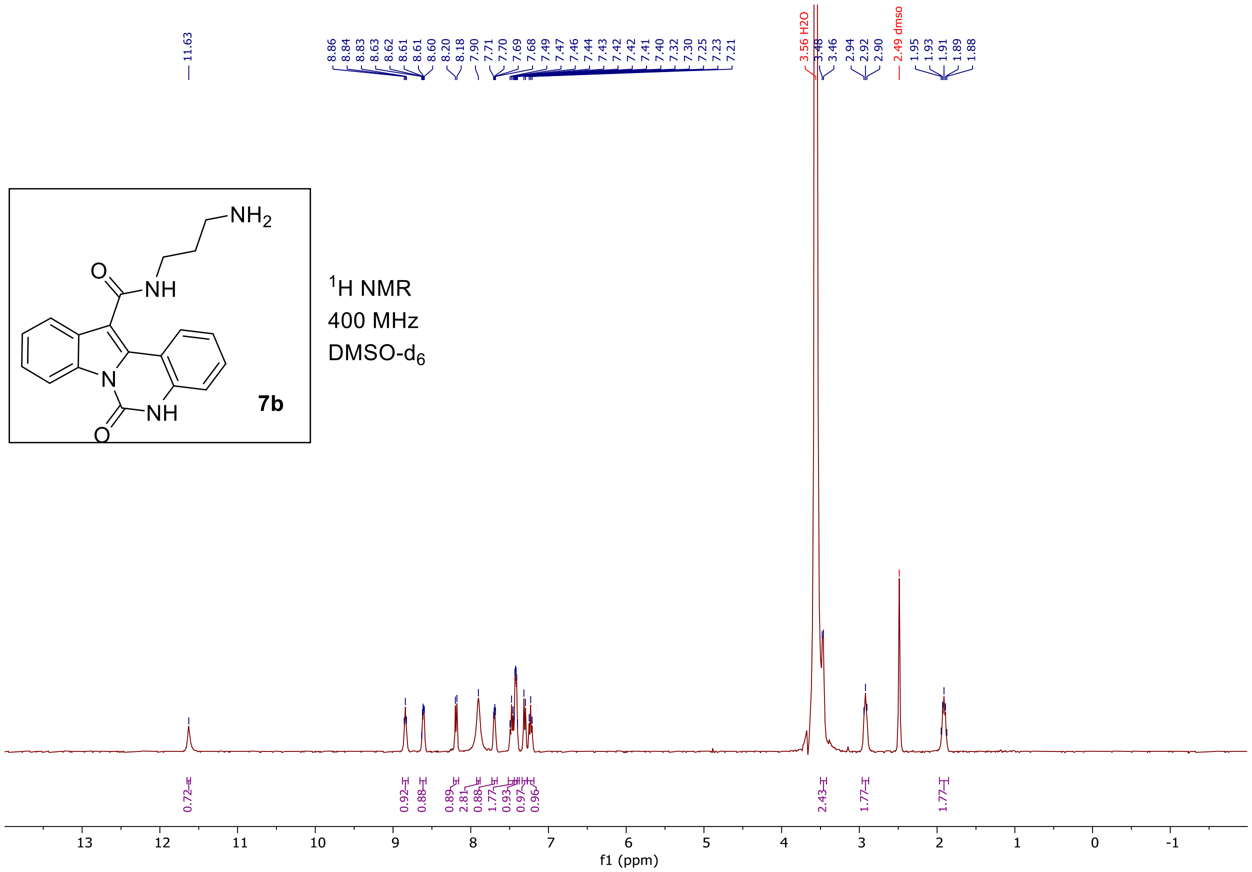

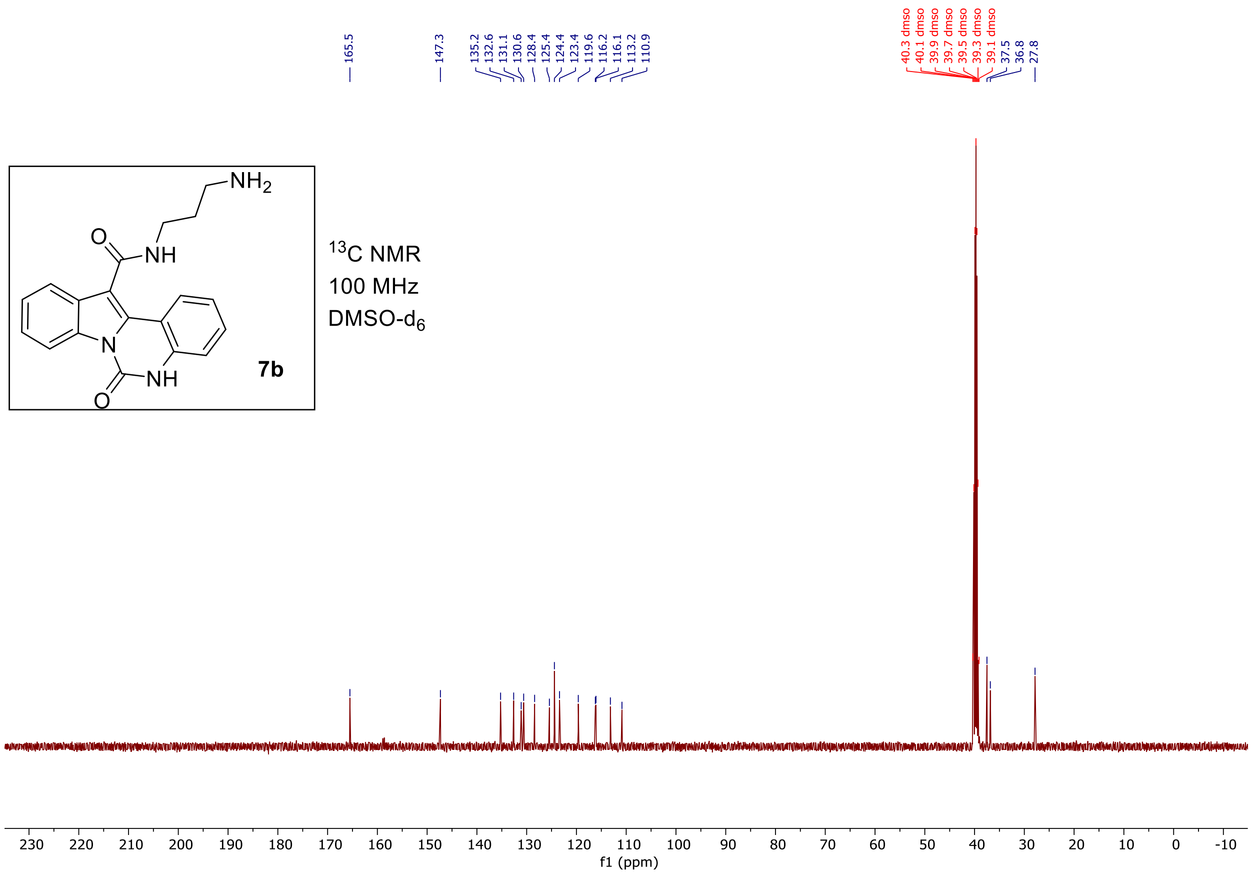

Figure S16. ^{1}H and ^{13}C NMR spectra of compound **7b**.

Compound Spectrum List Report

Analysis Info

Analysis Name	D:\Data\AST\KDV-27.d	Acquisition Date	6/28/2025
Method	tune_norm.m	Operator	Mitrokhov
Sample Name	Tune wide	Instrument / Ser#	micrOTOF-Q II 10225
Comment			

Acquisition Parameter

Source Type	ESI	Ion Polarity	Positive	Set Nebulizer	0.4 Bar
Focus	Active	Set Capillary	4500 V	Set Dry Heater	180 °C
Scan Begin	50 m/z	Set End Plate Offset	-500 V	Set Dry Gas	4.0 l/min
Scan End	3000 m/z	Set Collision Cell RF	550.0 Vpp	Set Divert Valve	Source

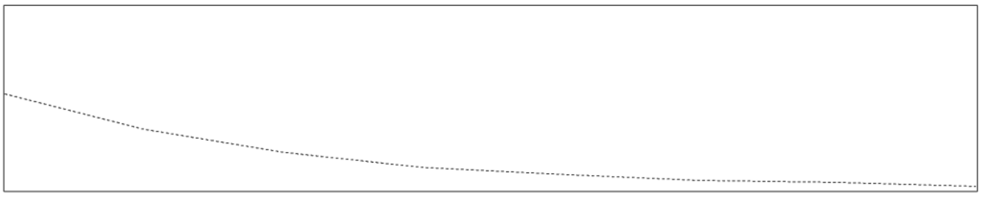

#	RT [min]	Area	Int. Type	Intens.	S/N	Chromatogram	Max. m/z
n.a.	0.1	n.a.	Average spectrum	n.a.	n.a.	n.a.	335.1486

+MS, 0.1-0.1min #(3-8)

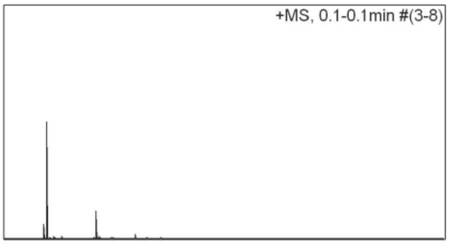

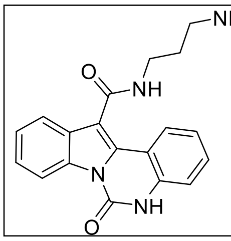

7b

#	m/z	Res.	S/N	I	I %
1	318.1144	11708	2866.0	321619	12.4
2	319.1154	9841	638.1	71992	2.8
3	335.1486	17021	21214.2	2587161	100.0
4	336.1451	14394	4966.8	608581	23.5
5	337.1441	9185	481.6	59311	2.3
6	669.2787	17169	2994.0	603620	23.3
7	670.2784	14220	1205.2	243723	9.4
8	671.2763	12170	310.2	62978	2.4
9	929.3248	12257	455.7	88673	3.4
10	930.3276	12121	310.8	60457	2.3

Bruker Compass DataAnalysis 4.0 printed: 6/28/2025 6:22:11 PM Page 1 of 1

Figure S17. Copy of HRMS (ESI) spectra of compound **7b**.

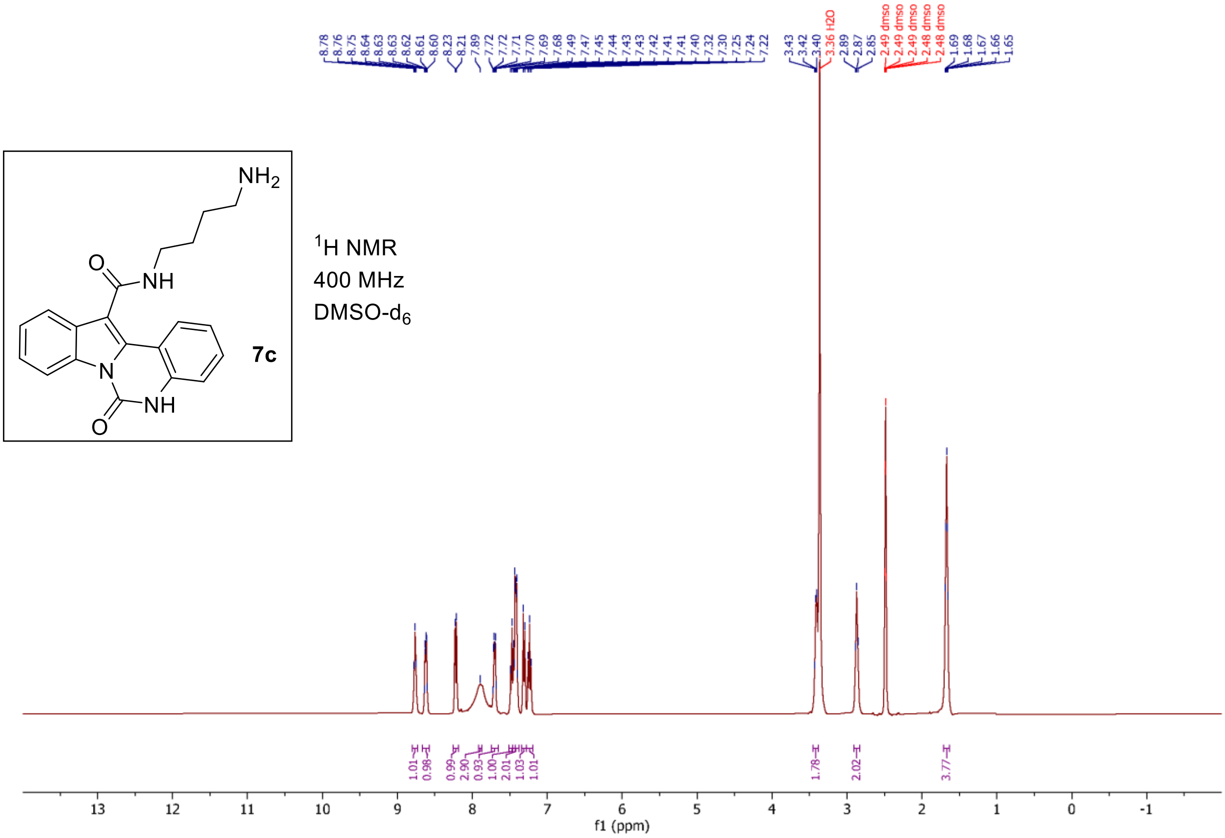

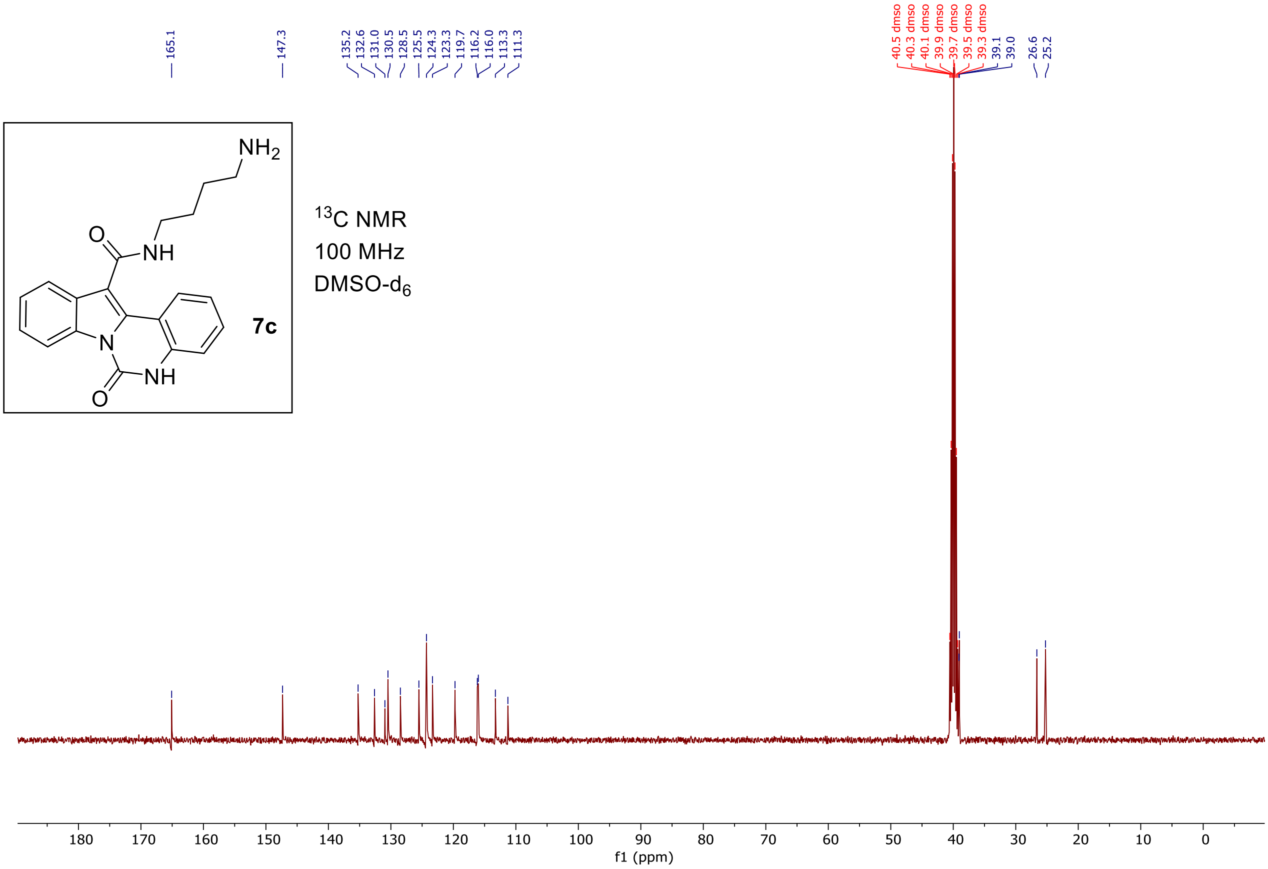

Figure S18. ^{1}H and ^{13}C NMR spectra of compound **7c**.

Compound Spectrum List Report

Analysis Info

Analysis Name	D:\Data\AST\KDV-28.d	Acquisition Date	6/9/2024
Method	tune_norm.m	Operator	Mitrokhov
Sample Name	Tune wide	Instrument / Ser#	micrOTOF-Q II 10225
Comment			

Acquisition Parameter

Source Type	ESI	Ion Polarity	Positive	Set Nebulizer	0.6 Bar
Focus	Not active	Set Capillary	4500 V	Set Dry Heater	180 °C
Scan Begin	50 m/z	Set End Plate Offset	-500 V	Set Dry Gas	4.0 l/min
Scan End	3000 m/z	Set Collision Cell RF	550.0 Vpp	Set Divert Valve	Source

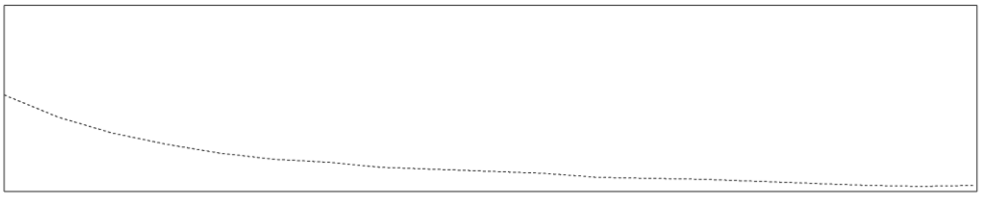

#	RT [min]	Area	Int. Type	Intens.	S/N	Chromatogram	Max. m/z
n.a.	0.2	n.a.	Average spectrum	n.a.	n.a.	n.a.	349.1673

+MS, 0.1-0.3min #(3-18)

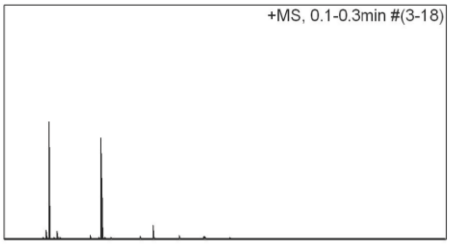

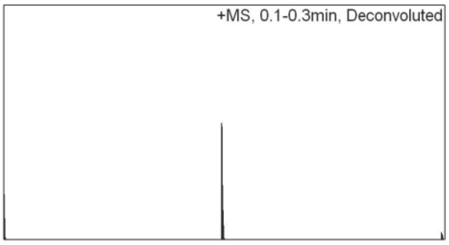

#	m/z	Res.	S/N	I	I %
1	332.1389	5280	1545.3	87619	6.9
2	349.1673	3159	21914.5	1274998	100.0
3	350.1705	4522	18015.1	1046257	82.1
4	351.1707	5483	2939.7	170508	13.4
5	405.2268	5532	1518.0	79519	6.2
6	697.3236	5225	13524.1	1096323	86.0
7	698.3251	5829	7962.4	647661	50.8
8	699.3257	6150	1995.4	162937	12.8
9	1045.4787	6654	1517.2	143555	11.3
10	1046.4810	6583	1095.3	103876	8.1

#	m/z	Res.	S/N	I	I %
1	350.1700			1046256	44.1
2	697.3238			2371320	100.0
3	1045.4777			143554	6.1

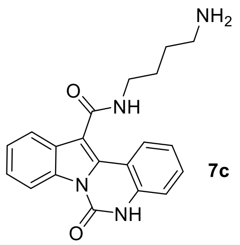

7c

Figure S19. Copy of HRMS (ESI) spectra of compound **7c**.

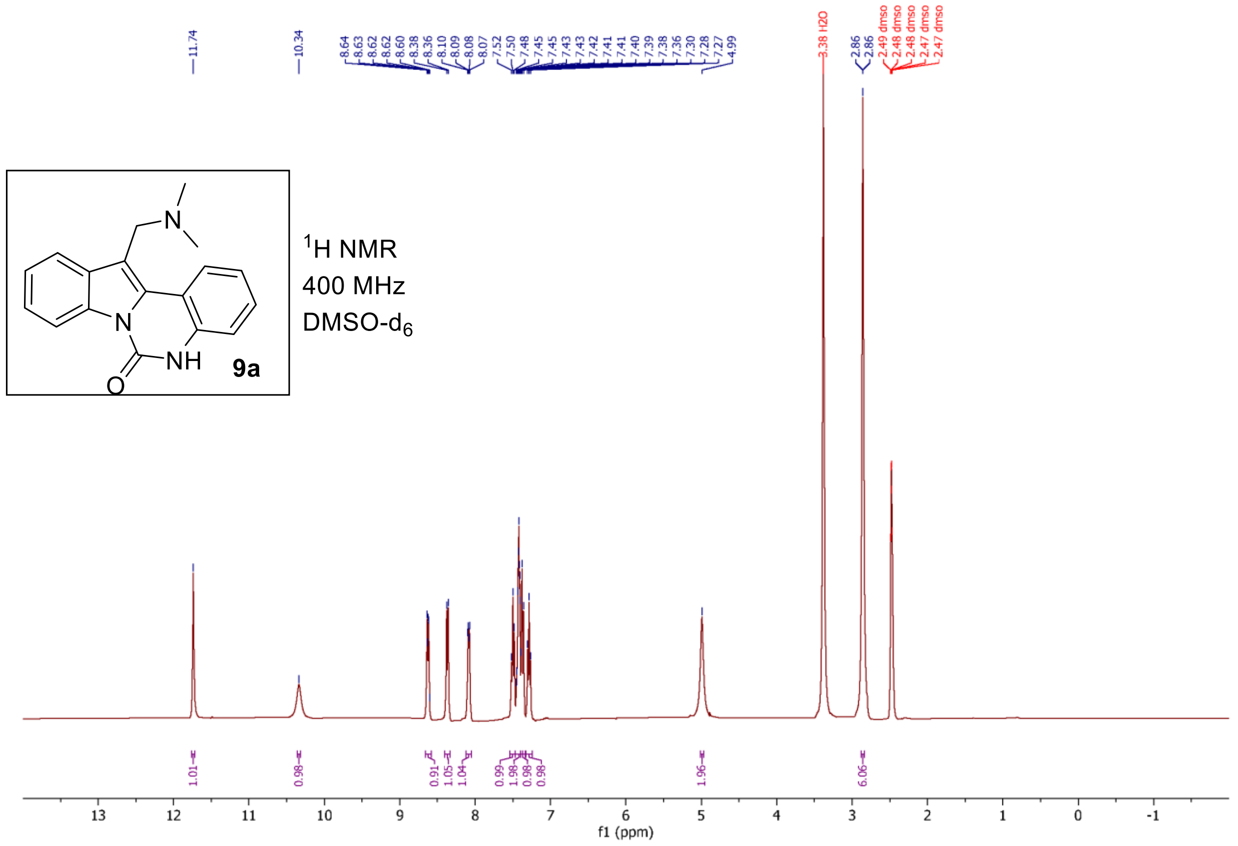

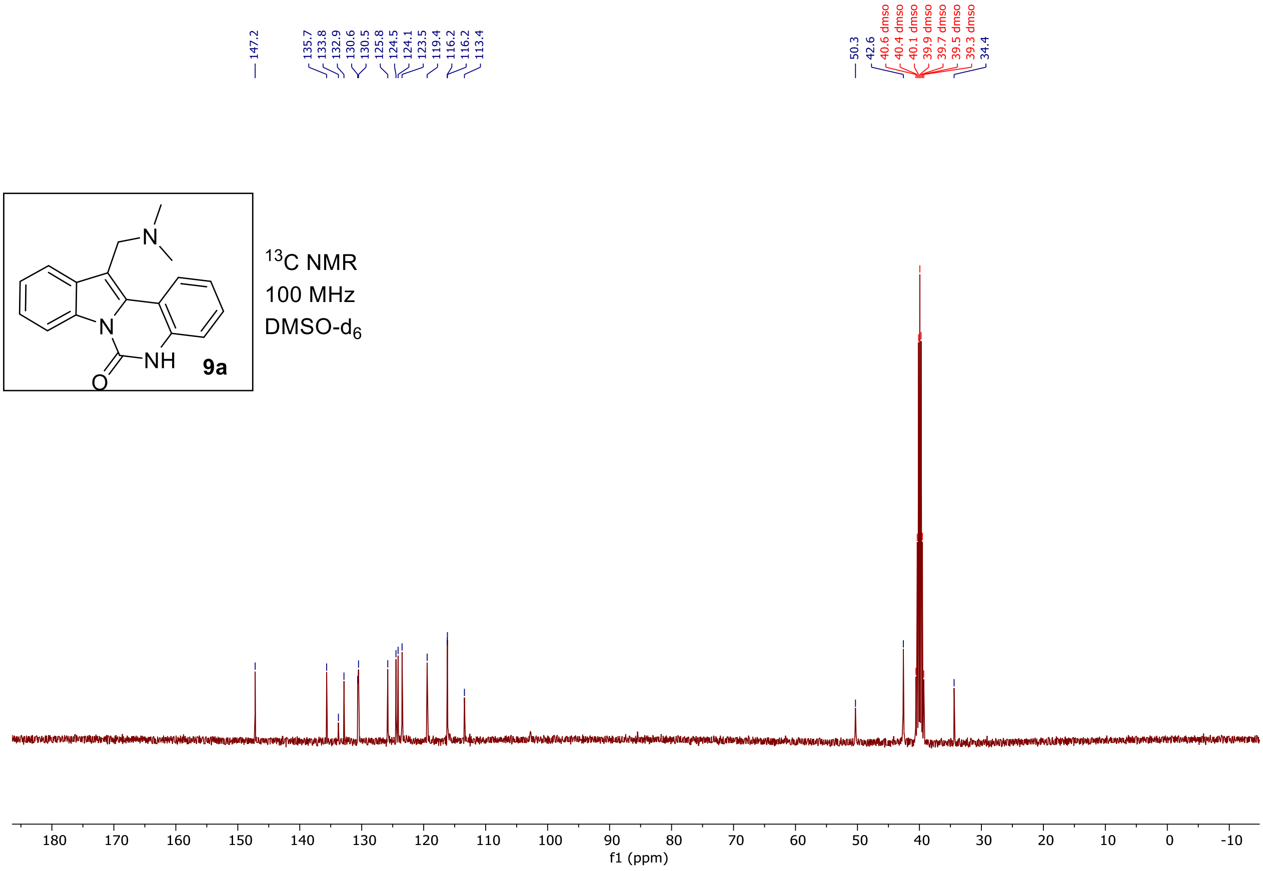

Figure S20. ^{1}H and ^{13}C NMR spectra of compound **9a**.

Compound Spectrum List Report

Analysis Info

Analysis Name	D:\Data\AST\KDV-19.d	Acquisition Date	6/27/2025
Method	tune_low_norm.m	Operator	Mitrokhov
Sample Name	Tune wide	Instrument / Ser#	micrOTOF-Q II 10225
Comment			

Acquisition Parameter

Source Type	ESI	Ion Polarity	Positive	Set Nebulizer	0.4 Bar
Focus	Active	Set Capillary	4500 V	Set Dry Heater	180 °C
Scan Begin	50 m/z	Set End Plate Offset	-500 V	Set Dry Gas	4.0 l/min
Scan End	3000 m/z	Set Collision Cell RF	150.0 Vpp	Set Divert Valve	Source

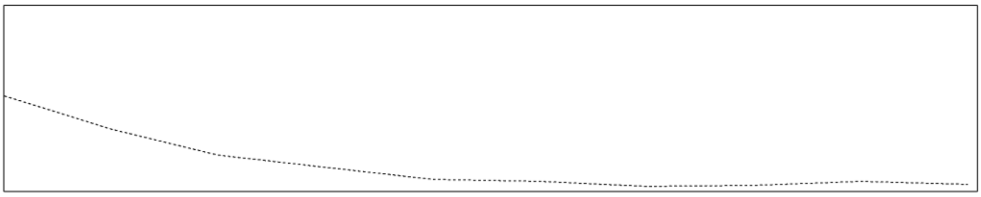

#	RT [min]	Area	Max. m/z
n.a.	0.1	n.a.	247.0883

+MS, 0.1-0.2min #(5-10)

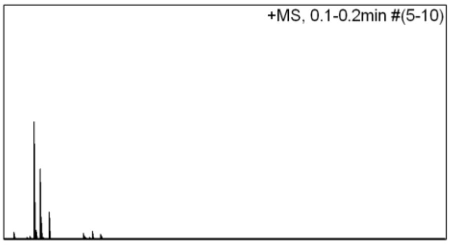

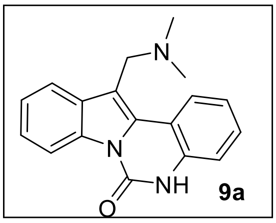

#	m/z	I	I %
1	115.0900	137779	5.4
2	247.0883	2556611	100.0
3	248.0943	1582119	61.9
4	249.0941	183317	7.2
5	261.0680	188773	7.4
6	292.1453	1521845	59.5
7	293.1498	415190	16.2
8	349.1681	571824	22.4
9	350.1689	137309	5.4
10	640.3008	153898	6.0

Bruker Compass DataAnalysis 4.0 printed: 6/27/2025 Page 1 of 1

Figure S21. Copy of HRMS (ESI) spectra of compound **9a**.

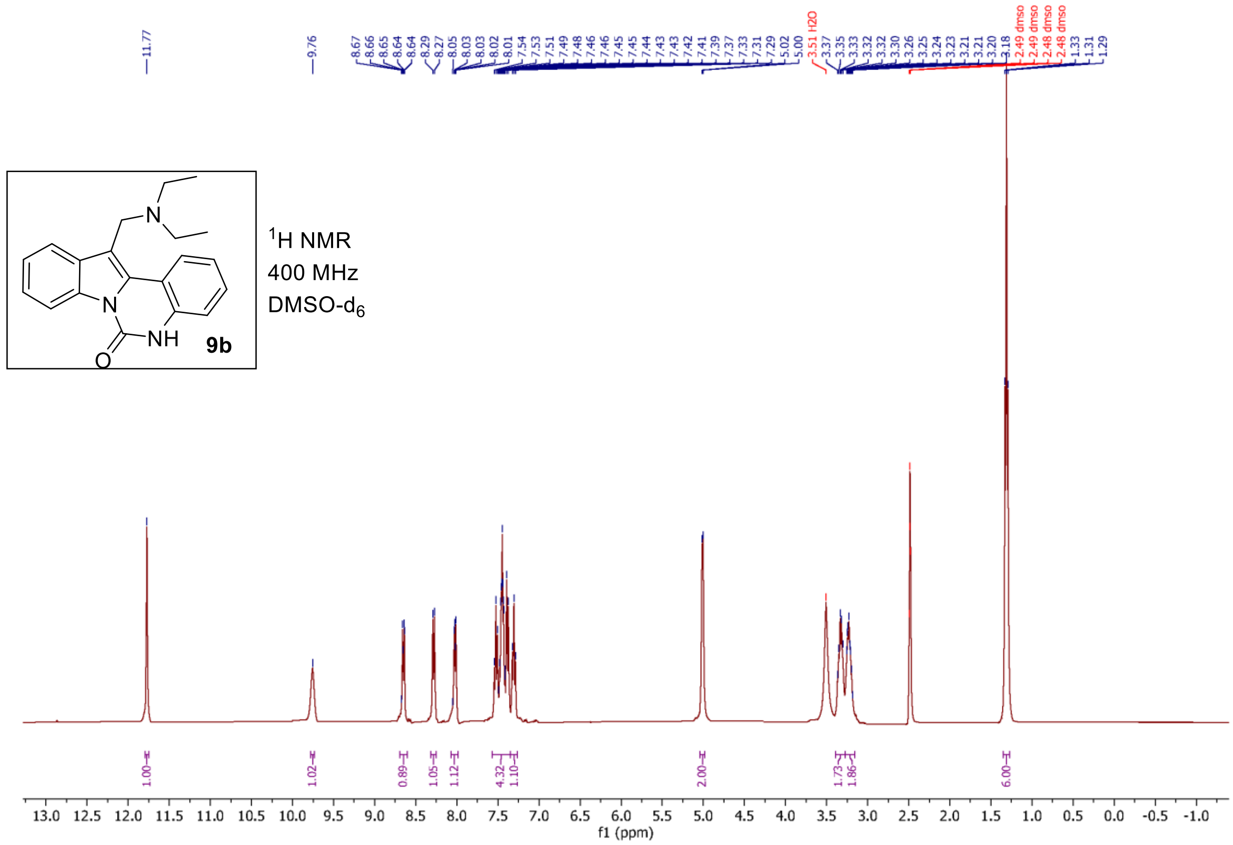

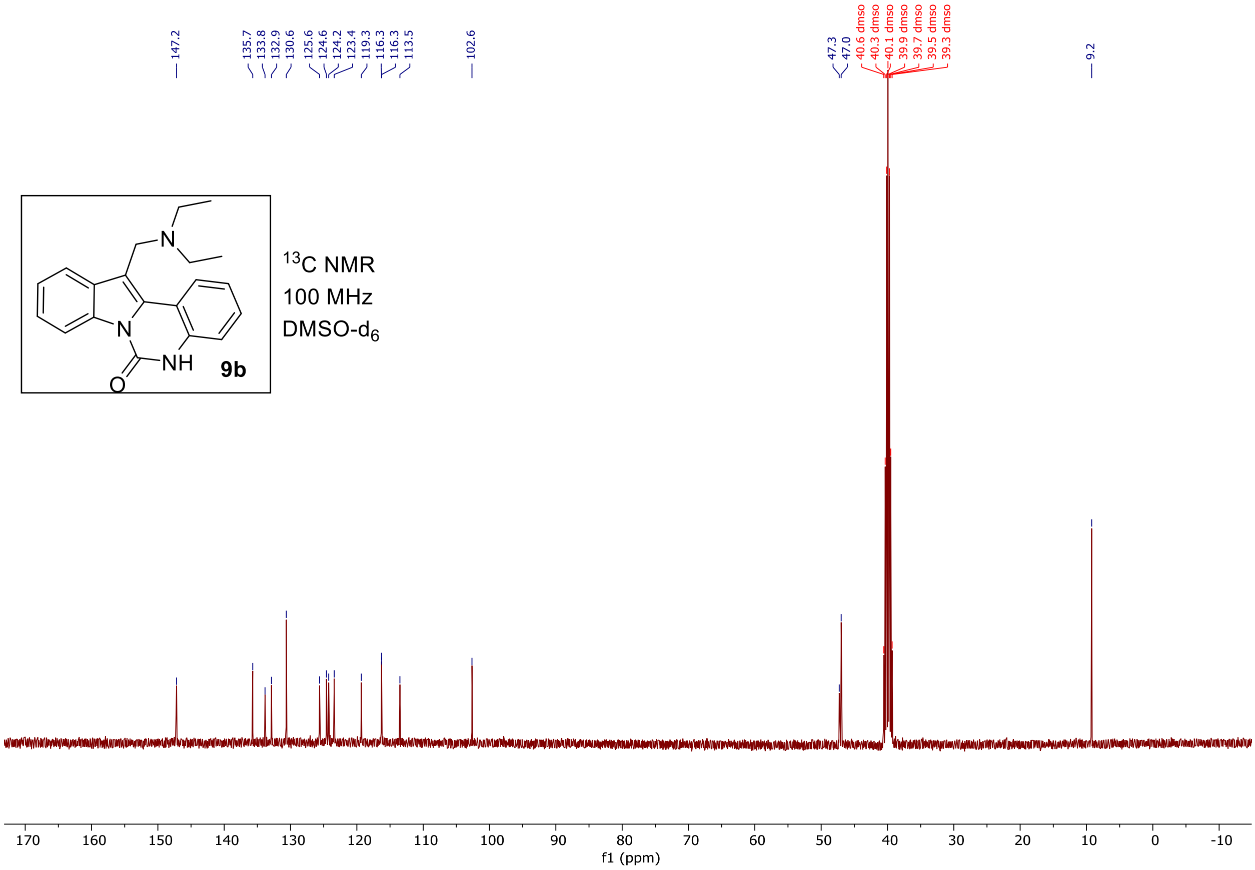

Figure S22. ^{1}H and ^{13}C NMR spectra of compound **9b**.

Compound Spectrum List Report

Analysis Info

Analysis Name	D:\Data\AST\KDV-44.d	Acquisition Date	6/27/2024
Method	tune_norm.m	Operator	Mitrokhov
Sample Name	Tune wide	Instrument / Ser#	micrOTOF-Q II 10225
Comment			

Acquisition Parameter

Source Type	ESI	Ion Polarity	Positive	Set Nebulizer	0.4 Bar
Focus	Not active	Set Capillary	4500 V	Set Dry Heater	180 °C
Scan Begin	50 m/z	Set End Plate Offset	-500 V	Set Dry Gas	4.0 l/min
Scan End	3000 m/z	Set Collision Cell RF	550.0 Vpp	Set Divert Valve	Source

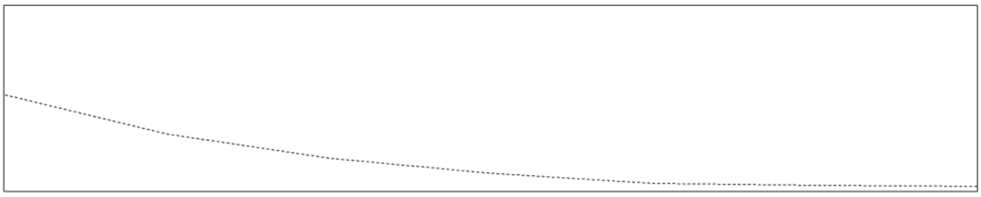

#	RT [min]	Area	Int. Type	Intens.	S/N	Chromatogram	Max. m/z
n.a.	0.1	n.a.	Average spectrum	n.a.	n.a.	n.a.	320.1773

+MS, 0.0-0.1min #(2-6)

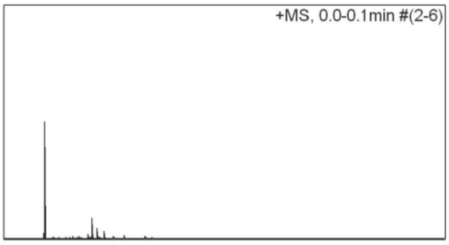

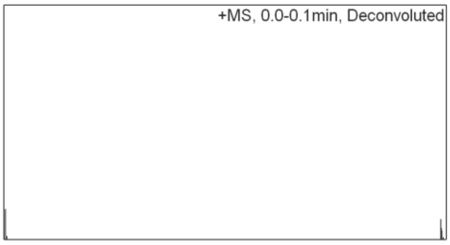

#	m/z	Res.	S/N	I	I %
1	318.1796	6044	841.3	47019	4.7
2	320.1773	5214	17569.6	989922	100.0
3	321.1996	5989	4628.9	261982	26.5
4	639.3817	6893	1521.8	171463	17.3
5	640.3845	7090	681.9	77084	7.8
6	675.3617	7048	697.9	85281	8.6
7	676.3639	7081	324.2	39773	4.0
8	677.3601	7233	299.6	36848	3.7
9	719.3121	7250	397.5	53214	5.4
10	721.3117	7249	422.2	56727	5.7

#	m/z	Res.	S/N	I	I %
1	320.1970			989922	100.0
2	639.3815			171463	17.3

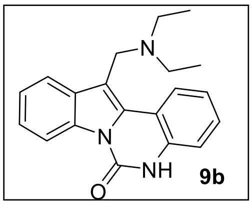

Figure S23. Copy of HRMS (ESI) spectra of compound **9b**.

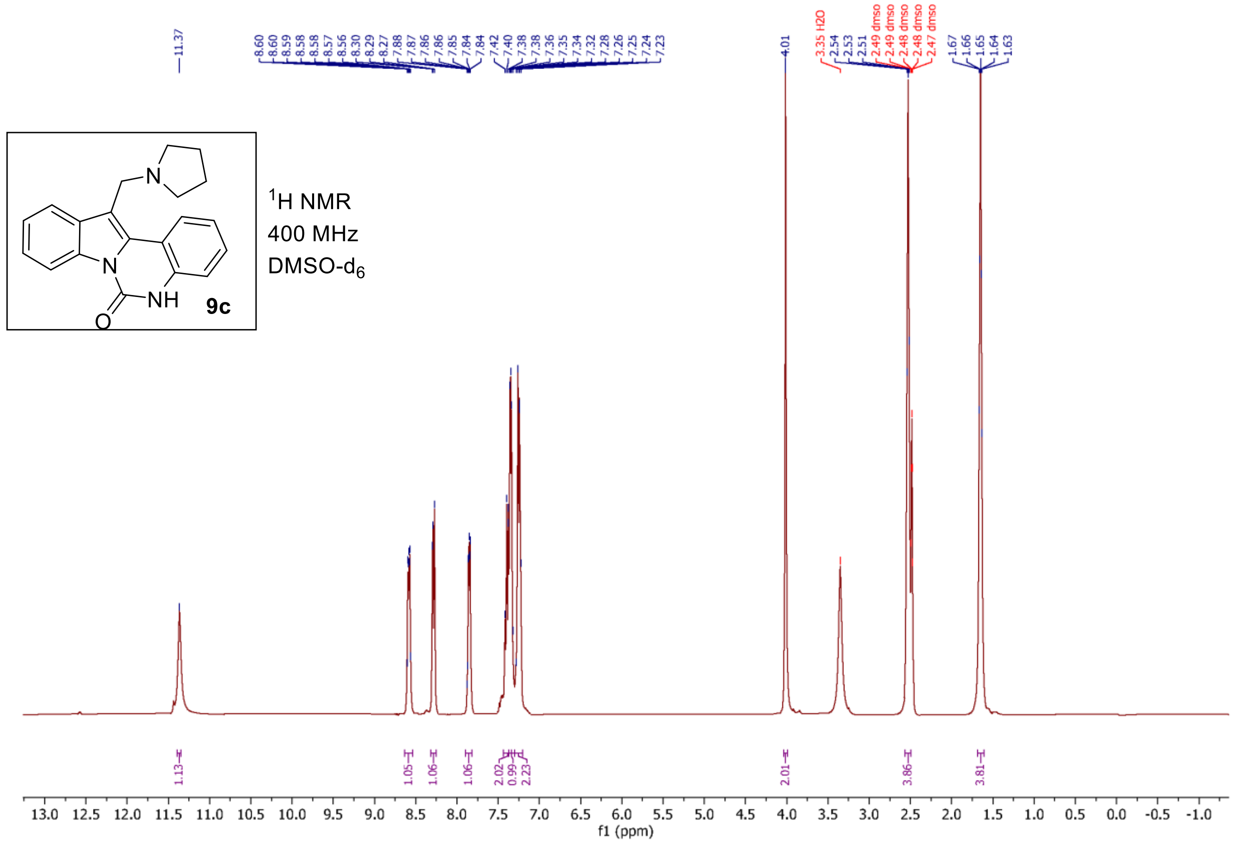

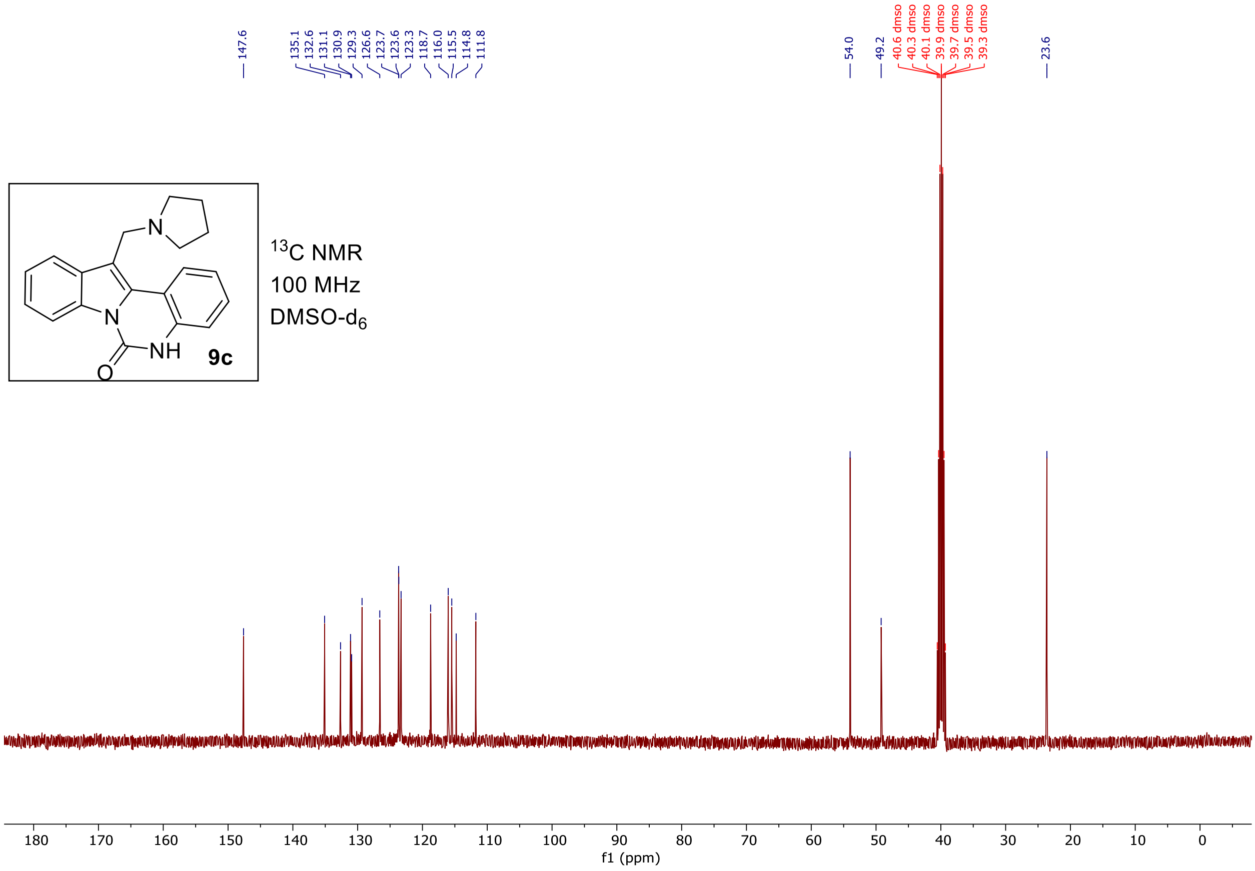

Figure S24. ^{1}H and ^{13}C NMR spectra of compound **9c**.

Compound Spectrum List Report

Analysis Info

Analysis Name	D:\Data\AST\KDV-46.d	Acquisition Date	6/29/2025
Method	tune_norm.m	Operator	Mitrokhov
Sample Name	Tune wide	Instrument / Ser#	micrOTOF-Q II 10225
Comment			

Acquisition Parameter

Source Type	ESI	Ion Polarity	Positive	Set Nebulizer	0.4 Bar
Focus	Not active	Set Capillary	4500 V	Set Dry Heater	180 °C
Scan Begin	50 m/z	Set End Plate Offset	-500 V	Set Dry Gas	4.0 l/min
Scan End	3000 m/z	Set Collision Cell RF	550.0 Vpp	Set Divert Valve	Source

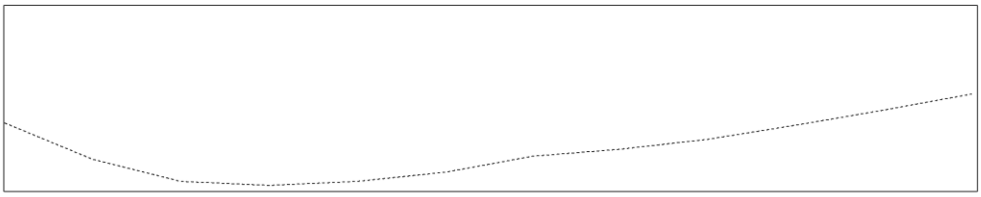

#	RT [min]	Area	Int. Type	Intens.	S/N	Chromatogram	Max. m/z
n.a.	0.1	n.a.	Average spectrum	n.a.	n.a.	n.a.	318.1613

+MS, 0.0-0.1min #(2-6)

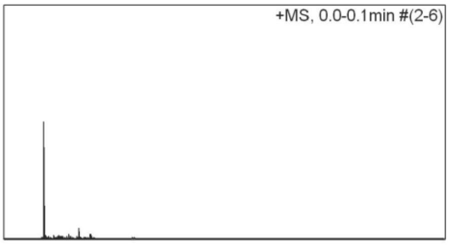

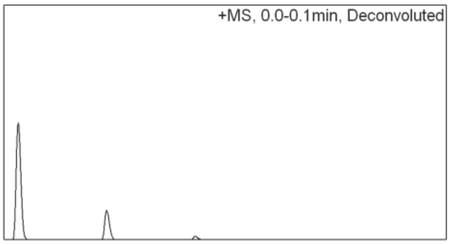

#	m/z	Res.	S/N	I	I %
1	316.1466	6032	705.0	70728	7.9
2	318.1613	5308	8870.8	897815	100.0
3	319.1660	5841	2266.7	230529	25.7
4	320.1676	5988	273.9	28028	3.1
5	383.2056	6230	222.7	26494	3.0
6	487.2371	6685	217.7	29474	3.3
7	552.2454	6624	541.9	76290	8.5
8	553.2467	6863	216.0	30452	3.4
9	625.2972	6726	246.2	31841	3.5
10	635.3166	6834	219.1	28003	3.1

#	m/z	Res.	S/N	I	I %
1	318.1635			897815	100.0

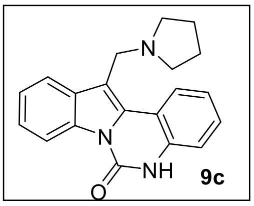

9c

Bruker Compass DataAnalysis 4.0 printed: 6/29/2025 8:52:06 PM Page 1 of 1

Figure S25. Copy of HRMS (ESI) spectra of compound **9c**.

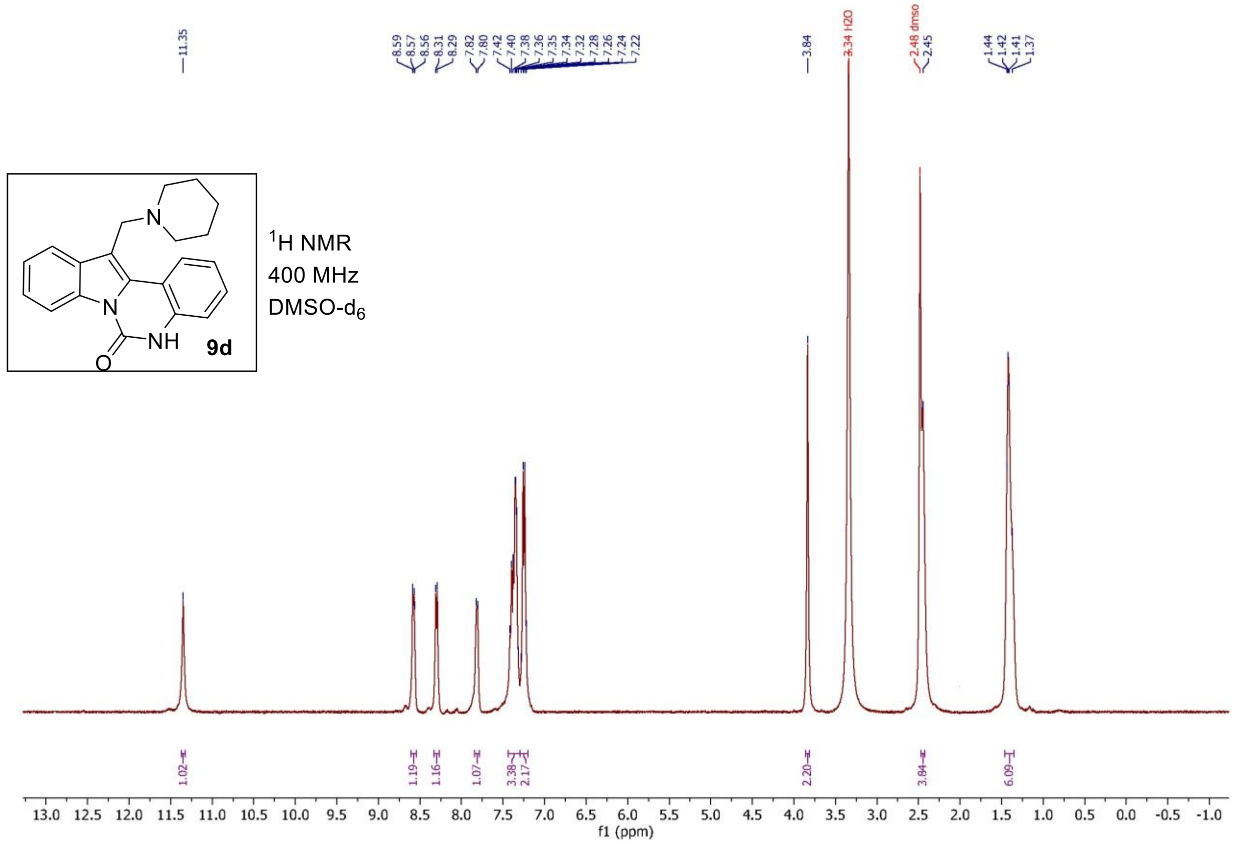

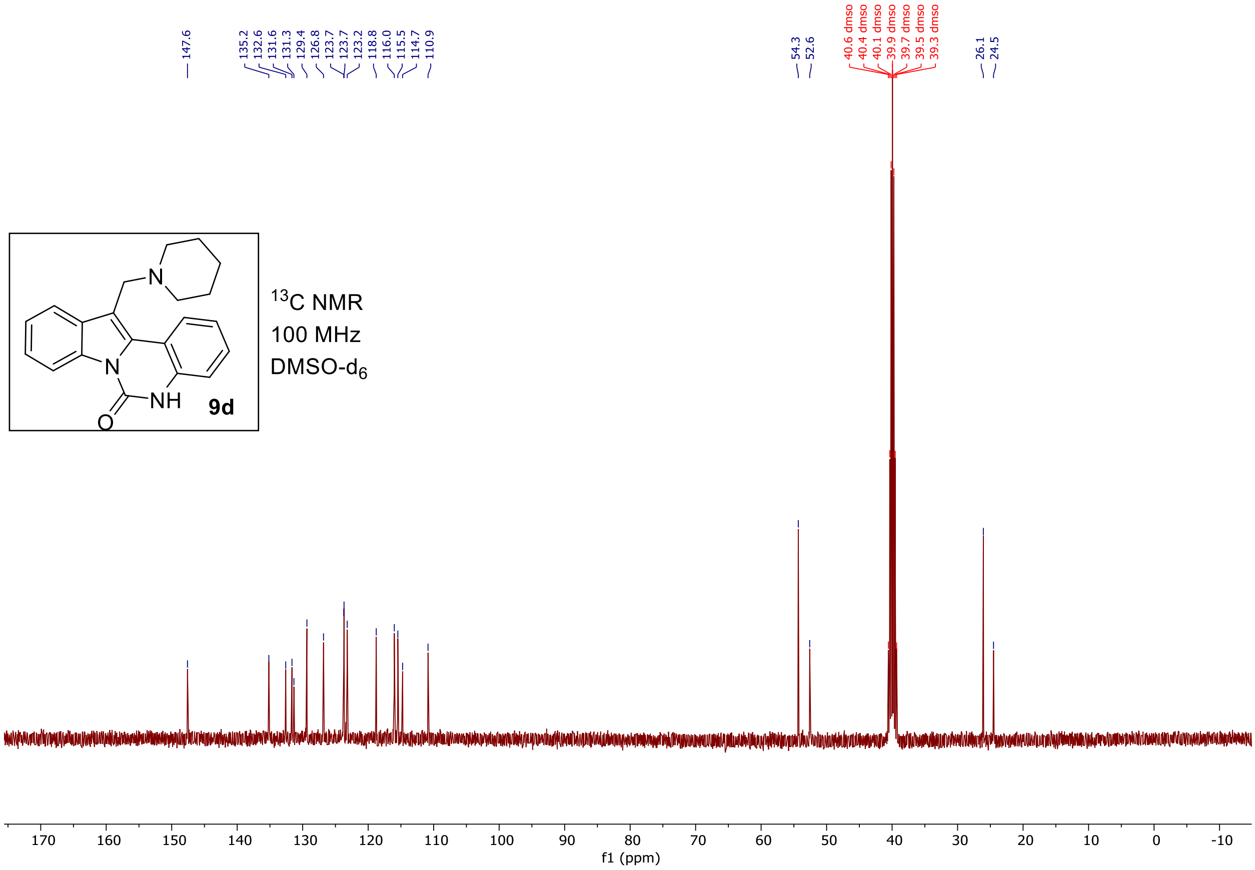

Figure S26. ^{1}H and ^{13}C NMR spectra of compound **9d**.

Compound Spectrum List Report

Analysis Info

Analysis Name	D:\Data\AST\KDV-25.d	Acquisition Date	10/27/2023
Method	tune_norm.m	Operator	Mitrokhov
Sample Name	Tune wide	Instrument / Ser#	micrOTOF-Q II 10225
Comment			

Acquisition Parameter

Source Type	ESI	Ion Polarity	Positive	Set Nebulizer	0.4 Bar
Focus	Active	Set Capillary	4500 V	Set Dry Heater	180 °C
Scan Begin	50 m/z	Set End Plate Offset	-500 V	Set Dry Gas	4.0 l/min
Scan End	3000 m/z	Set Collision Cell RF	550.0 Vpp	Set Divert Valve	Source

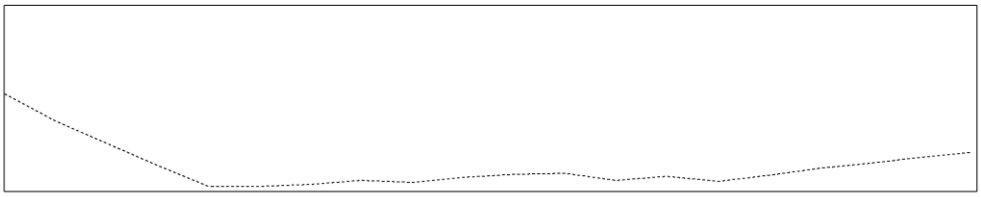

#	RT [min]	Area	Int. Type	Intens.	S/N	Chromatogram	Max. m/z
n.a.	0.2	n.a.	Average spectrum	n.a.	n.a.	n.a.	332.1743

+MS, 0.1-0.3min #(4-16)

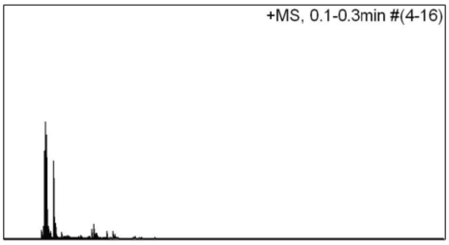

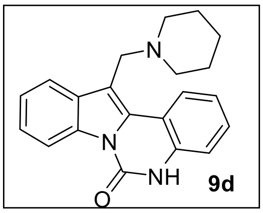

#	m/z	Res.	S/N	I	I %
1	321.1323	10768	1763.0	157431	75.2
2	322.1341	8857	331.2	29748	14.2
3	330.1572	9794	658.7	61624	29.4
4	332.1743	11492	2215.3	209297	100.0
5	333.1760	9009	486.1	46172	22.1
6	335.1488	11280	1937.4	185755	88.8
7	336.1502	8792	390.8	37672	18.0
8	383.1999	11592	1284.2	139226	66.5
9	384.2019	9950	262.1	28512	13.6
10	399.1708	10294	247.9	27839	13.3

Bruker Compass DataAnalysis 4.0 printed: 10/28/2023 6:19:17 PM Page 1 of 1

Figure S27. Copy of HRMS (ESI) spectra of compound **9d**.

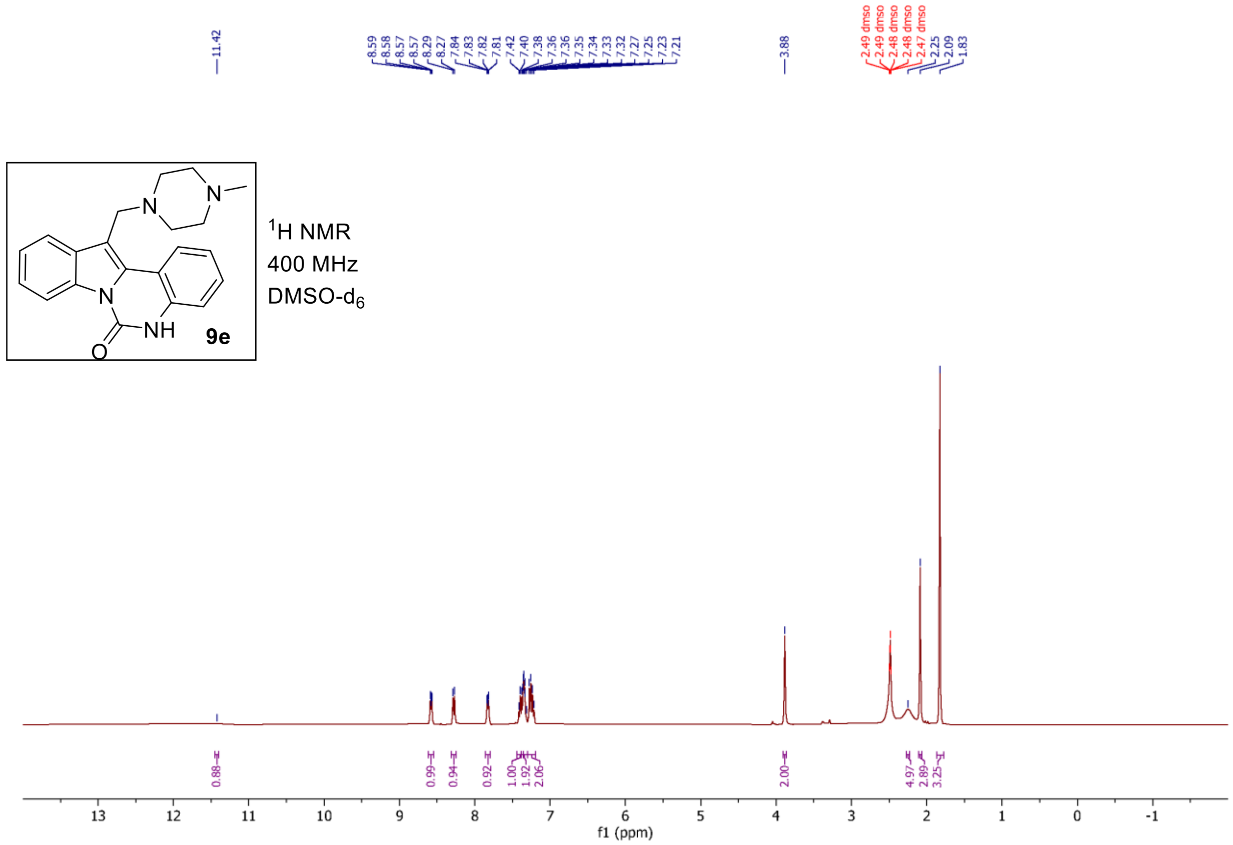

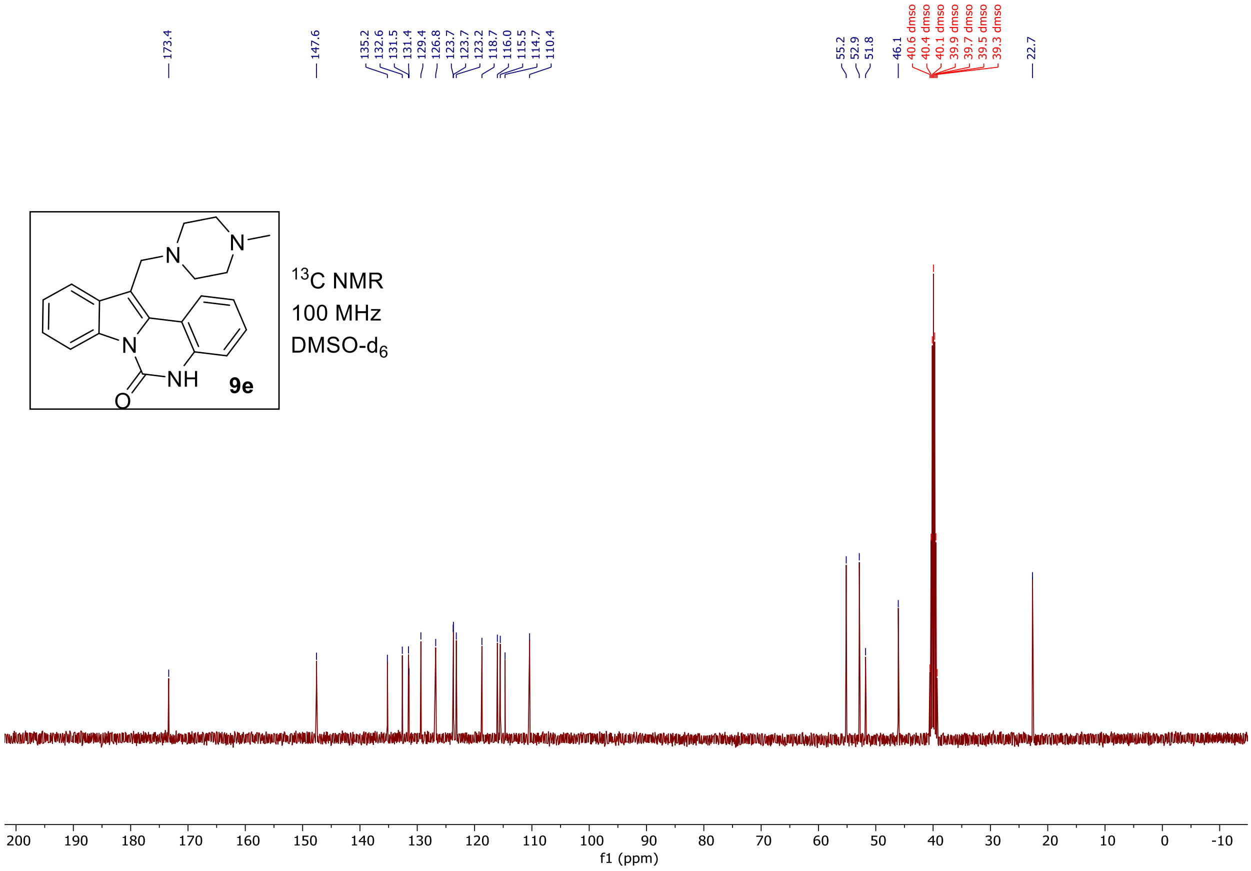

Figure S28. ^{1}H and ^{13}C NMR spectra of compound **9e**.

Compound Spectrum List Report

Analysis Info

Analysis Name	D:\Data\AST\KDV-23.d	Acquisition Date	6/27/2025
Method	tune_norm.m	Operator	Mitrokhov
Sample Name	Tune wide	Instrument / Ser#	micrOTOF-Q II 10225
Comment			

Acquisition Parameter

Source Type	ESI	Ion Polarity	Positive	Set Nebulizer	0.4 Bar
Focus	Active	Set Capillary	4500 V	Set Dry Heater	180 °C
Scan Begin	50 m/z	Set End Plate Offset	-500 V	Set Dry Gas	4.0 l/min
Scan End	3000 m/z	Set Collision Cell RF	550.0 Vpp	Set Divert Valve	Source

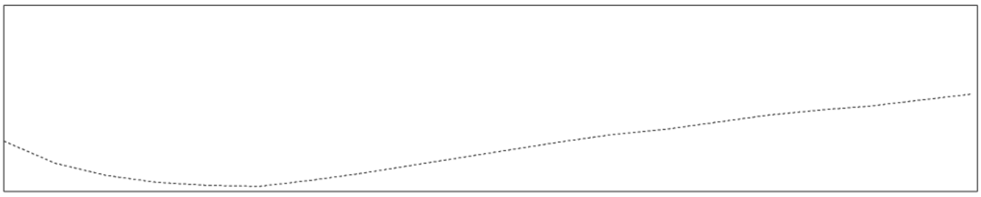

#	RT [min]	Area	Int. Type	Intens.	S/N	Chromatogram	Max. m/z
n.a.	0.2	n.a.	Average spectrum	n.a.	n.a.	n.a.	347.1881

+MS, 0.1-0.3min #(4-15)

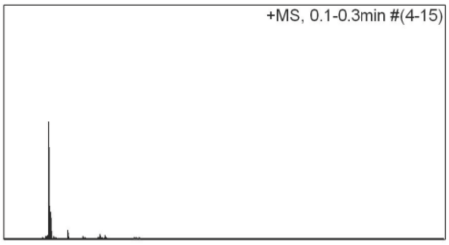

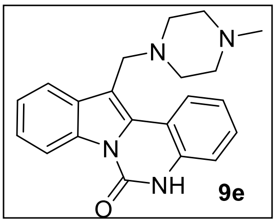

9e

#	m/z	Res.	S/N	I	I %
1	345.1687	10008	344.6	31916	2.4
2	347.1881	20336	14347.8	1328074	100.0
3	348.1889	12061	2464.3	228143	17.2
4	349.1892	8798	295.0	27338	2.1
5	363.1811	12595	3196.6	296385	22.3
6	364.1816	10243	749.7	69537	5.2
7	479.1453	10805	1024.0	96083	7.2
8	480.1474	9388	350.5	32925	2.5
9	693.3539	11944	398.2	50607	3.8
10	729.3343	9913	247.7	33393	2.5

Bruker Compass DataAnalysis 4.0 printed: 6/28/2025 6:17:33 PM Page 1 of 1

Figure S29. Copy of HRMS (ESI) spectra of compound **9e**.

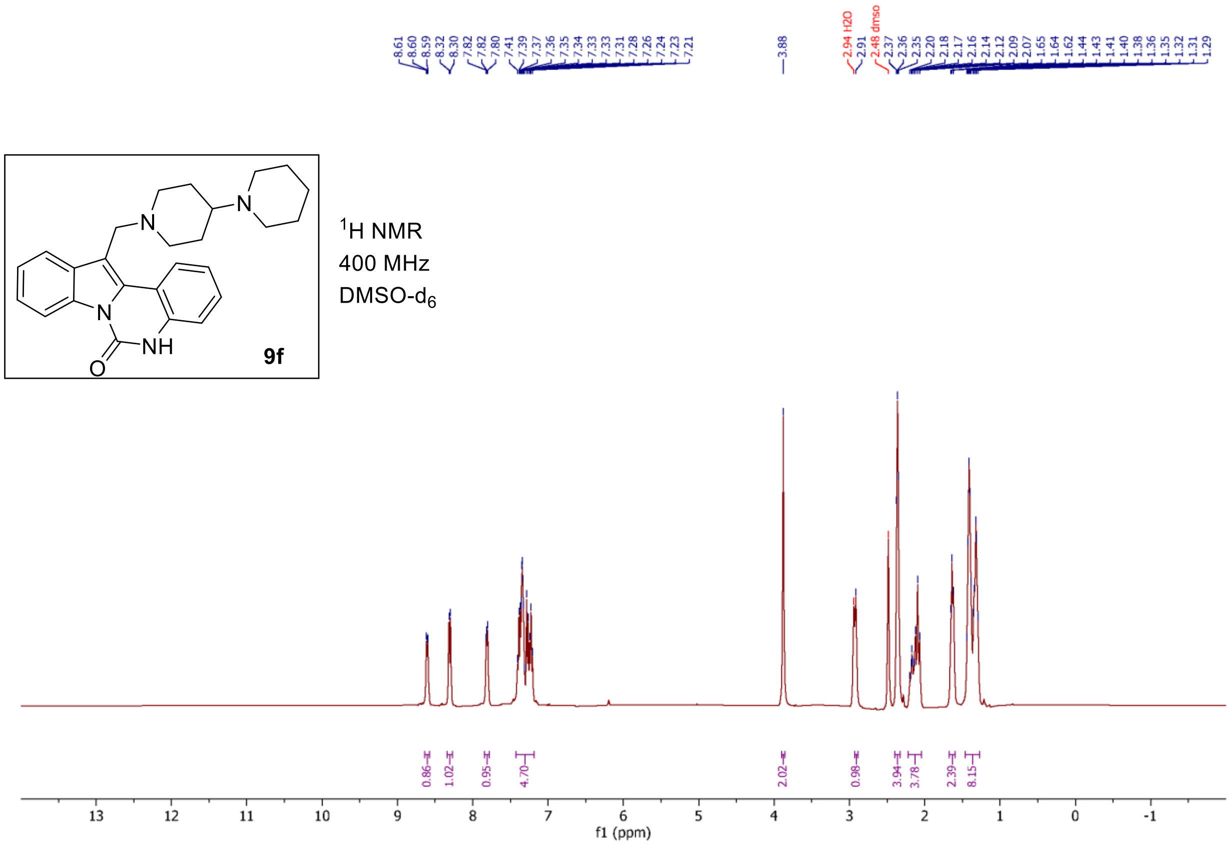

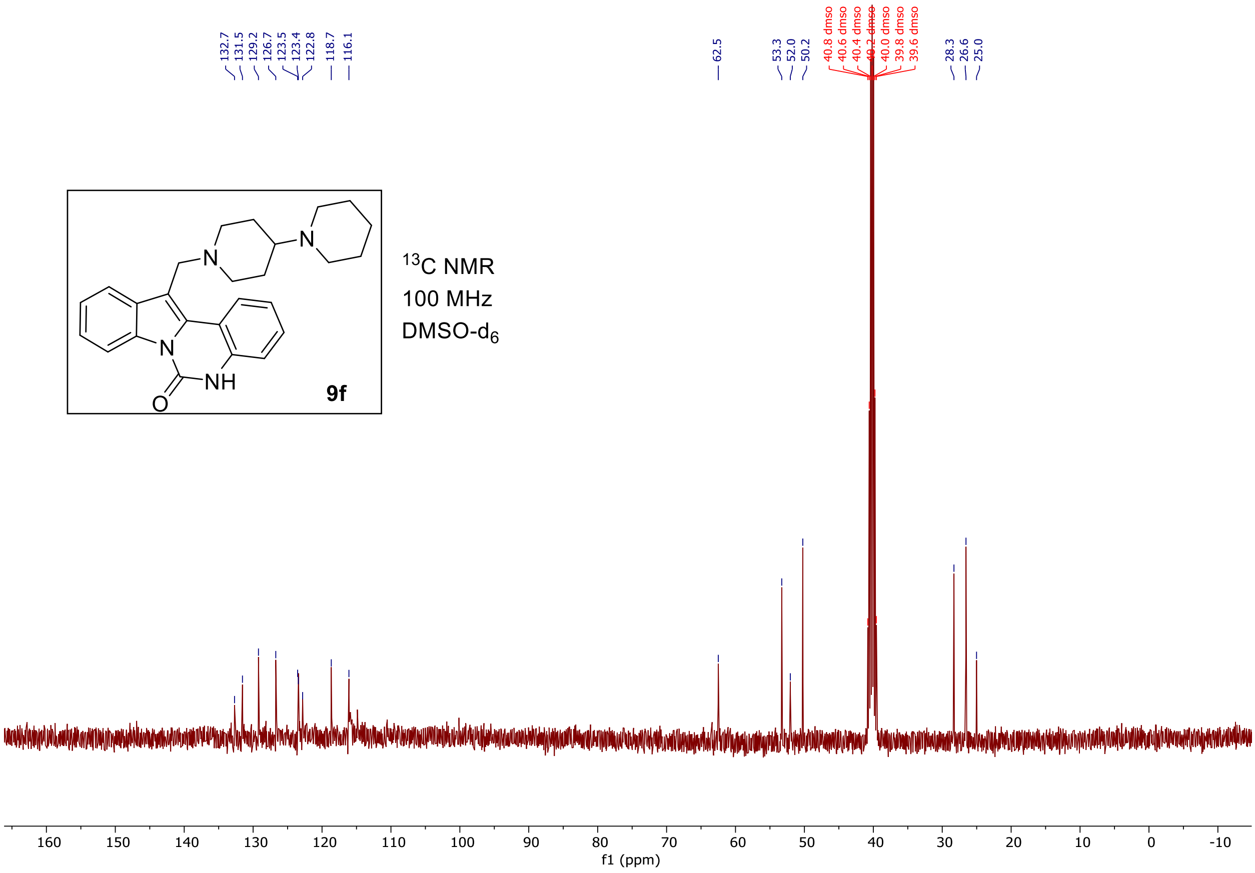

Figure S30. ^{1}H and ^{13}C NMR spectra of compound **9f**.

Compound Spectrum List Report

Analysis Info

Analysis Name	D:\Data\AST\KDV-43.d	Acquisition Date	6/27/2025
Method	tune_norm.m	Operator	Mitrokhov
Sample Name	Tune wide	Instrument / Ser#	micrOTOF-Q II 10225
Comment			

Acquisition Parameter

Source Type	ESI	Ion Polarity	Positive	Set Nebulizer	0.4 Bar
Focus	Not active	Set Capillary	4500 V	Set Dry Heater	180 °C
Scan Begin	50 m/z	Set End Plate Offset	-500 V	Set Dry Gas	4.0 l/min
Scan End	3000 m/z	Set Collision Cell RF	550.0 Vpp	Set Divert Valve	Source

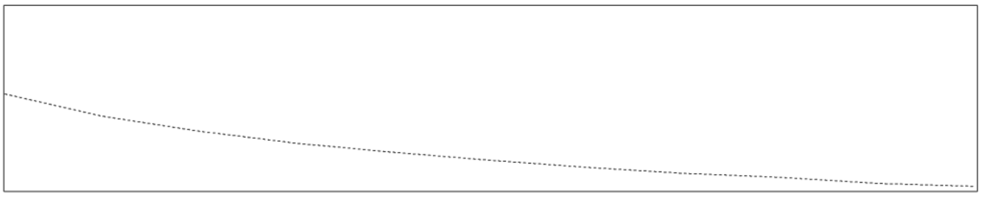

#	RT [min]	Area	Int. Type	Intens.	S/N	Chromatogram	Max. m/z
n.a.	0.1	n.a.	Average spectrum	n.a.	n.a.	n.a.	415.2498

+MS, 0.0-0.1min #(2-6)

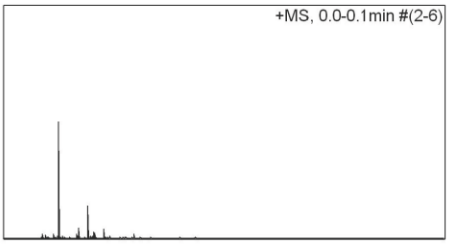

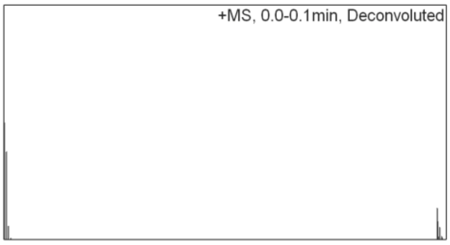

#	m/z	Res.	S/N	I	I %
1	415.2498	4350	11097.2	1274871	100.0
2	416.2595	5750	8475.9	973879	76.4
3	417.2611	6506	1264.0	145333	11.4
4	554.2263	6585	950.4	111419	8.7
5	615.2815	6542	2957.6	351538	27.6
6	616.2833	6647	1182.6	140675	11.0
7	657.2928	7077	540.6	64999	5.1
8	661.3388	7199	430.0	51777	4.1
9	722.3950	6978	787.5	96072	7.5
10	922.4232	6858	329.5	50065	3.9

#	m/z	Res.	S/N	I	I %
1	415.2558			1274870	100.0
2	615.2809			351537	27.6

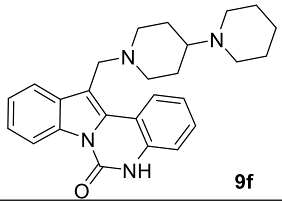

9f

Figure S31. Copy of HRMS (ESI) spectra of compound **9f**.

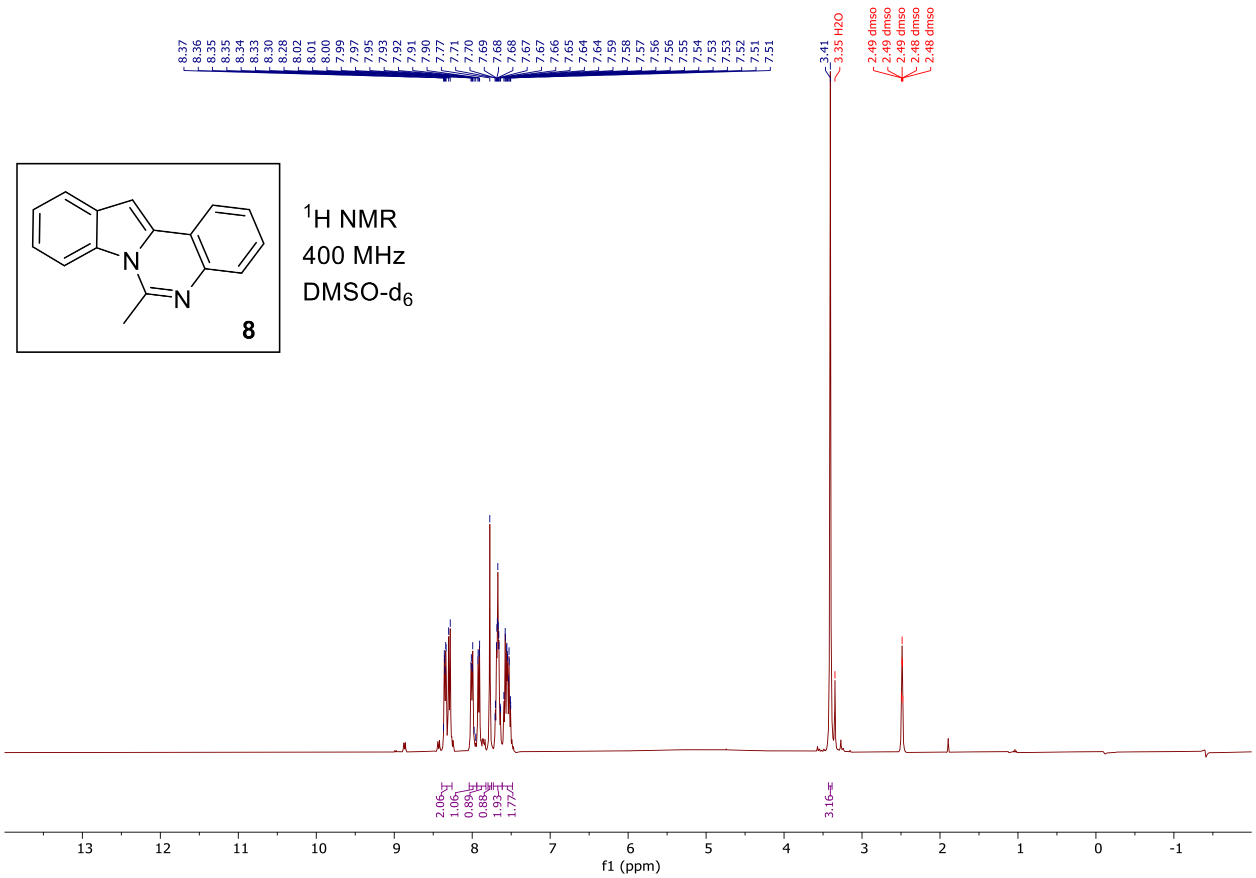

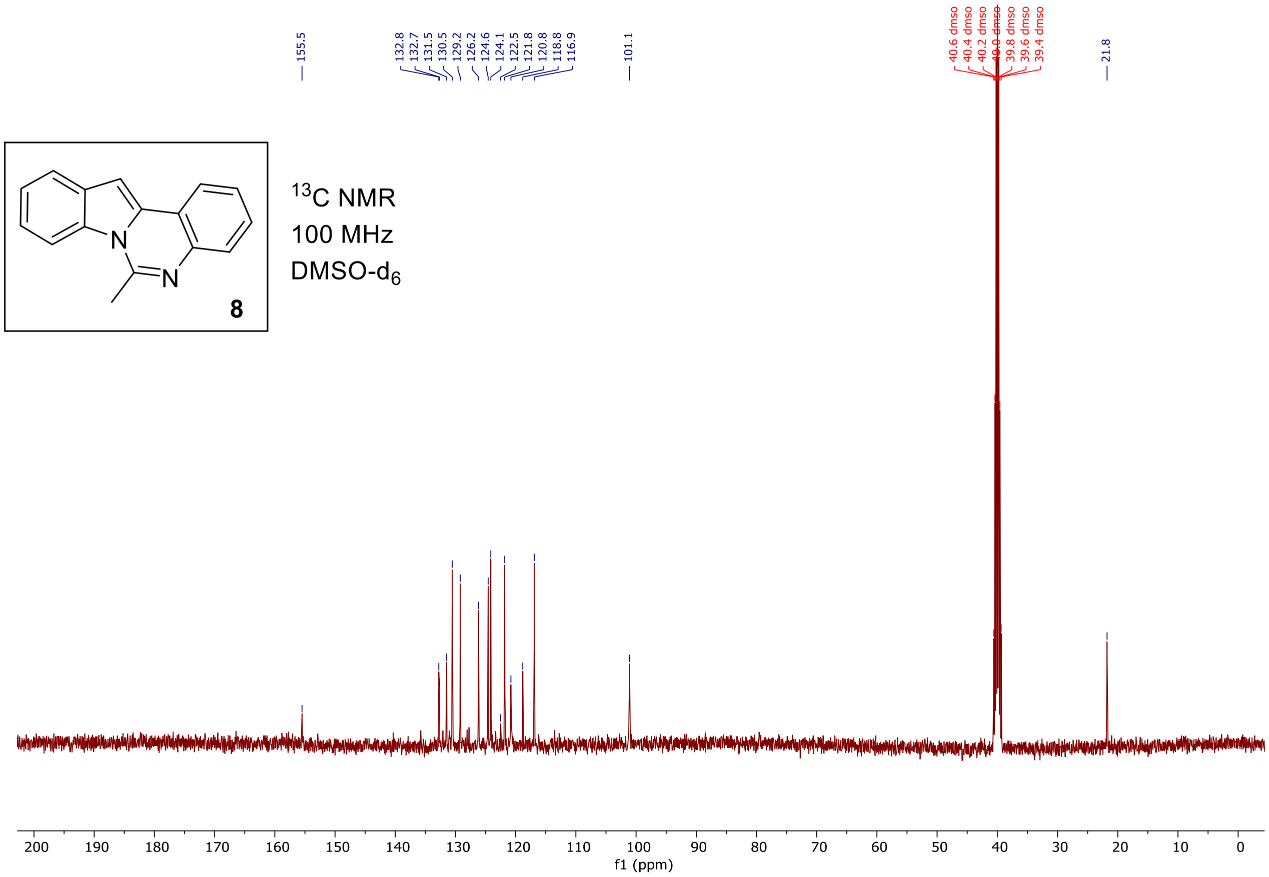

Figure S32. ^{1}H and ^{13}C NMR spectra of compound **9**.

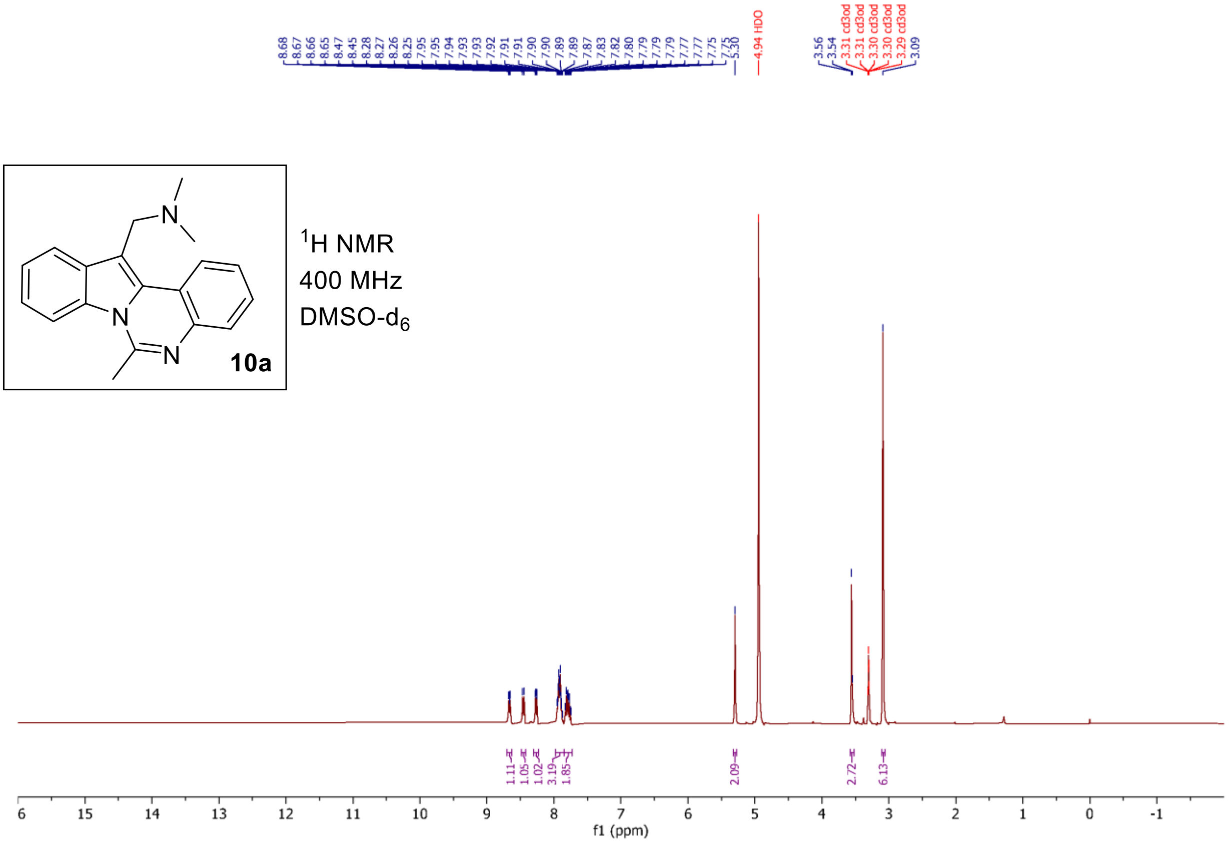

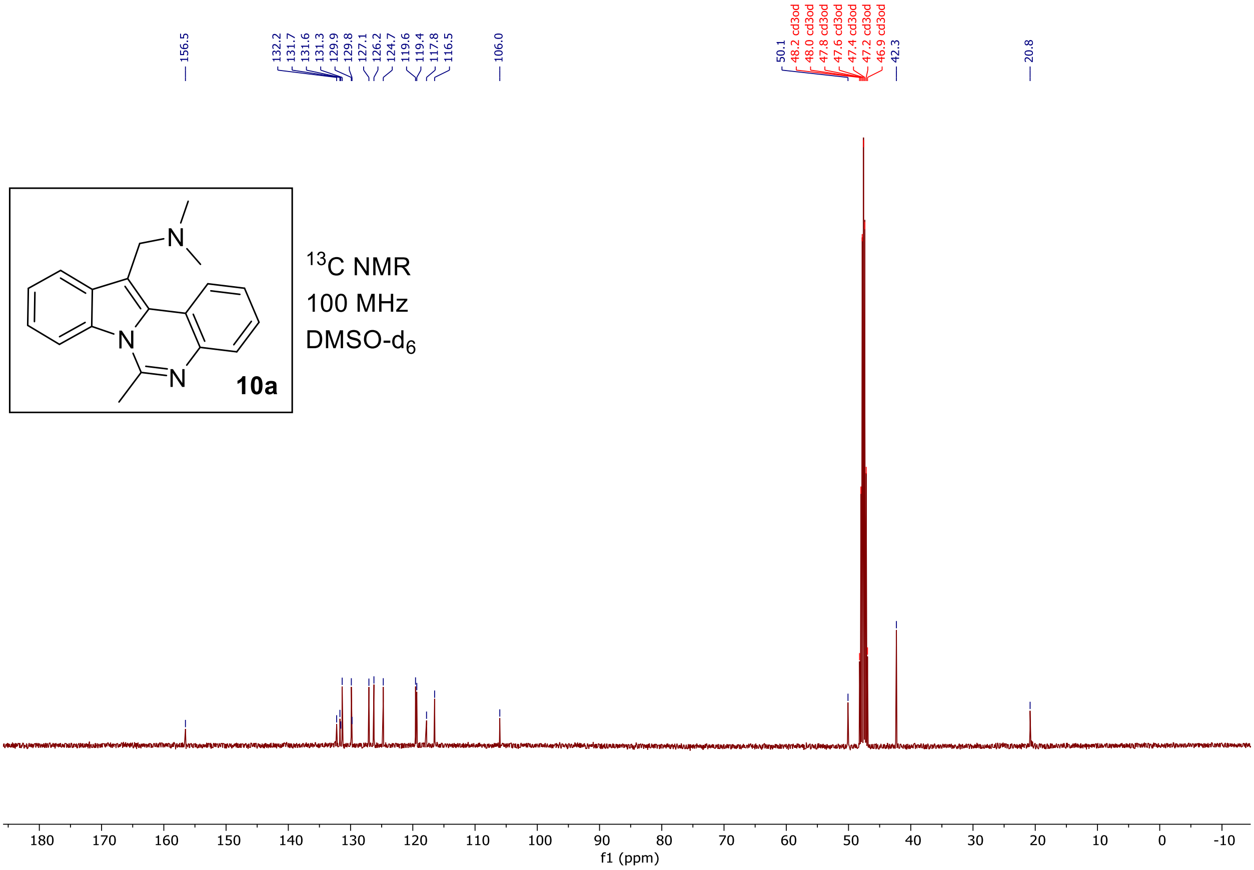

Figure S33. ^{1}H and ^{13}C NMR spectra of compound **10a**.

Compound Spectrum List Report

Analysis Info

Analysis Name	D:\Data\AST\KDV-39(1).d	Acquisition Date	6/26/2025
Method	tune_norm.m	Operator	Mitrokhov
Sample Name	Tune wide	Instrument / Ser#	micrOTOF-Q II 10225
Comment			

Acquisition Parameter

Source Type	ESI	Ion Polarity	Positive	Set Nebulizer	0.4 Bar
Focus	Not active	Set Capillary	4500 V	Set Dry Heater	180 °C
Scan Begin	50 m/z	Set End Plate Offset	-500 V	Set Dry Gas	4.0 l/min
Scan End	3000 m/z	Set Collision Cell RF	550.0 Vpp	Set Divert Valve	Source

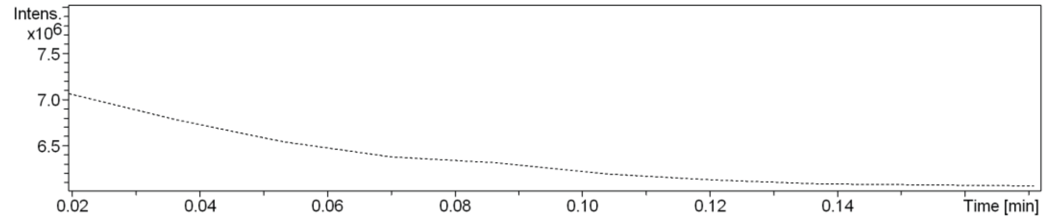

#	RT [min]	Area	Int. Type	Intens.	S/N	Chromatogram	Max. m/z
n.a.	0.1	n.a.	Average spectrum	n.a.	n.a.	n.a.	290.1664

+MS, 0.0-0.1min #(2-8)

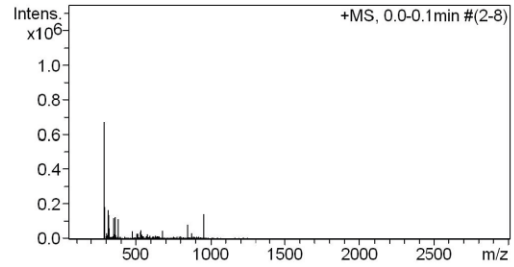

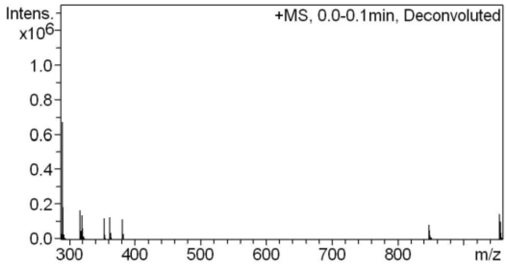

#	m/z	Res.	S/N	I	I %
1	288.1511	5309	60582.2	95201	14.2
2	290.1664	4711	425221.7	668206	100.0
3	291.1698	5231	113017.9	177600	26.6
4	317.1302	5261	103009.9	161873	24.2
5	320.1204	5216	84832.2	133308	20.0
6	353.2677	5462	73575.5	115619	17.3
7	362.1875	5499	77166.1	121261	18.1
8	381.2988	5442	69969.1	109951	16.5
9	952.6167	6326	89358.1	140420	21.0
10	953.6190	6412	61787.9	97095	14.5

#	m/z	Res.	S/N	I	I %
1	288.1511			95200	14.2
2	290.1677			668205	100.0
3	317.1312			161872	24.2
4	320.1230			133307	20.0
5	353.2678			115618	17.3
6	362.1871			121261	18.1
7	381.2988			109951	16.5
8	845.4804			76859	11.5
9	952.6159			140419	21.0

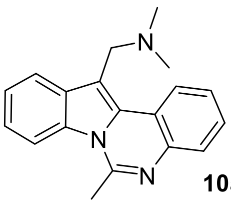

10a

Bruker Compass DataAnalysis 4.0 printed: 6/26/2025 3:34:11 PM Page 1 of 1

Figure S34. Copy of HRMS (ESI) spectra of compound **10a**.

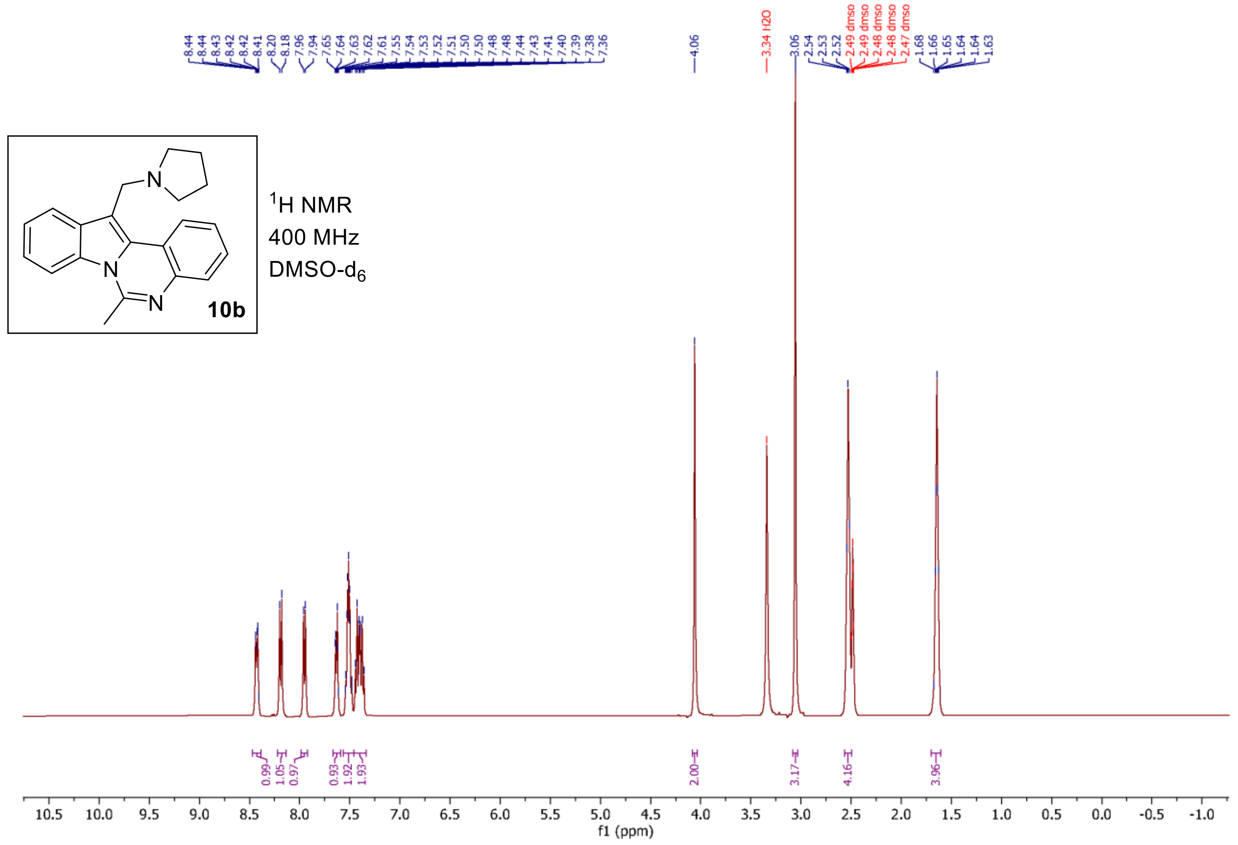

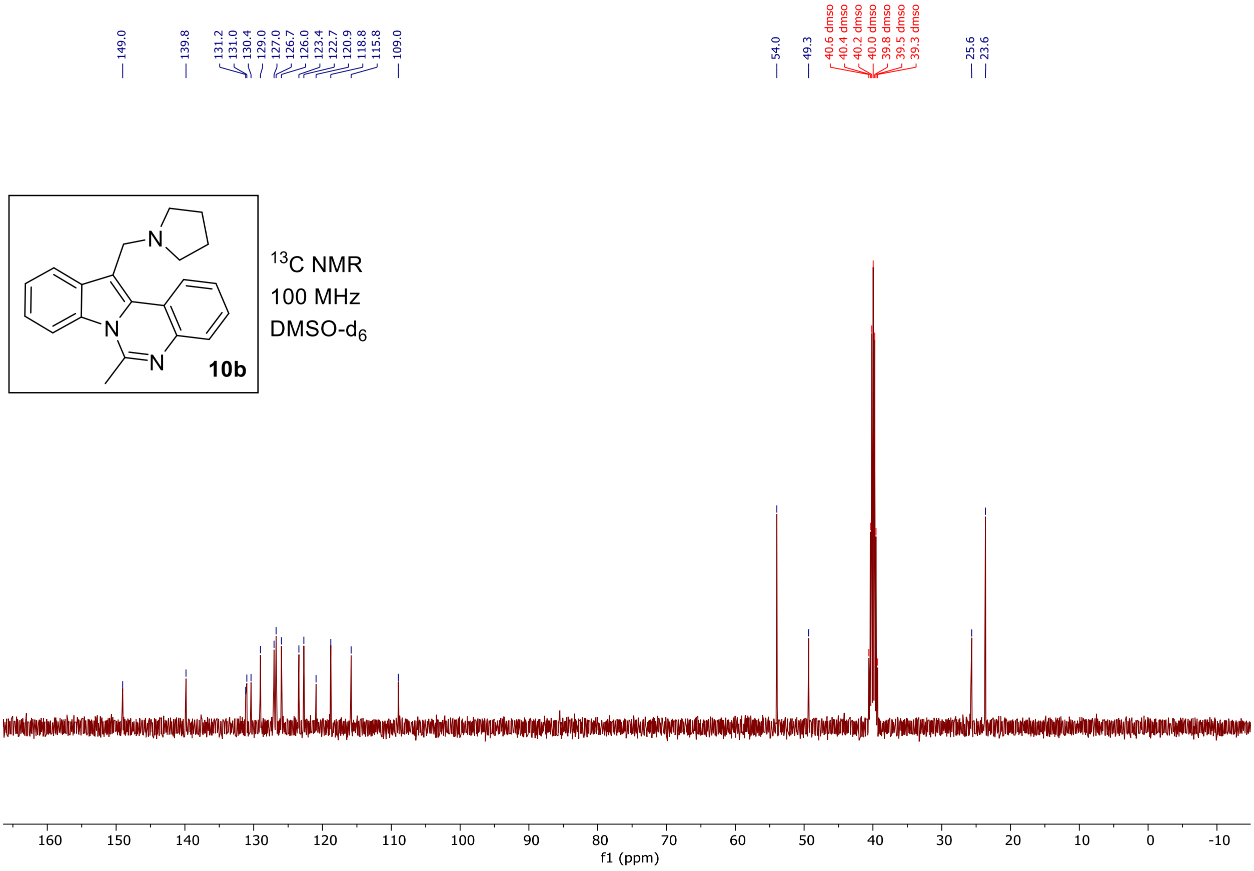

Figure S35. ^{1}H and ^{13}C NMR spectra of compound **10b**.

Compound Spectrum List Report

Analysis Info

Analysis Name	D:\Data\AST\KDV-69.d	Acquisition Date	3/1/2025
Method	tune_norm.m	Operator	Mitrokhov
Sample Name	Tune wide	Instrument / Ser#	micrOTOF-Q II 10225
Comment			

Acquisition Parameter

Source Type	ESI	Ion Polarity	Positive	Set Nebulizer	0.4 Bar
Focus	Not active	Set Capillary	4500 V	Set Dry Heater	180 °C
Scan Begin	50 m/z	Set End Plate Offset	-500 V	Set Dry Gas	4.0 l/min
Scan End	3000 m/z	Set Collision Cell RF	550.0 Vpp	Set Divert Valve	Source

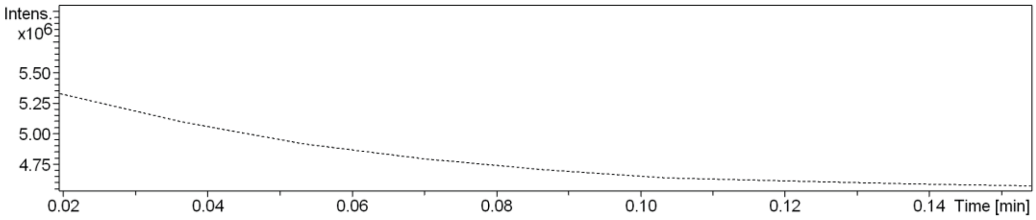

#	RT [min]	Area	Int. Type	Intens.	S/N	Chromatogram	Max. m/z
n.a.	0.1	n.a.	Average spectrum	n.a.	n.a.	n.a.	316.1819

+MS, 0.0-0.1min #(2-8)

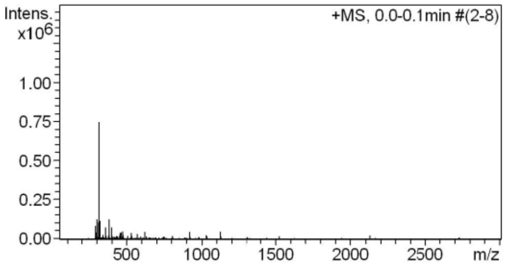

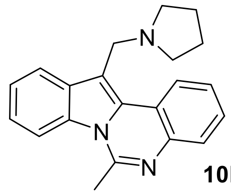

10b

#	m/z	Res.	S/N	I	I %
1	293.1746	5204	51249.3	80535	10.8
2	302.1653	5318	76733.9	120582	16.2
3	314.1629	5204	65558.2	103020	13.9
4	316.1819	4845	472432.5	742394	100.0
5	317.1835	5344	129812.6	203991	27.5
6	322.0483	5424	71338.6	112104	15.1
7	361.2228	5456	44991.9	70702	9.5
8	383.2037	5372	78445.2	123271	16.6
9	399.1779	5609	45014.5	70737	9.5
10	473.1098	5751	28759.5	45193	6.1

Bruker Compass DataAnalysis 4.0 printed: 3/1/2025 6:54:26 PM Page 1 of 1

Figure S36. Copy of HRMS (ESI) spectra of compound **10b**.

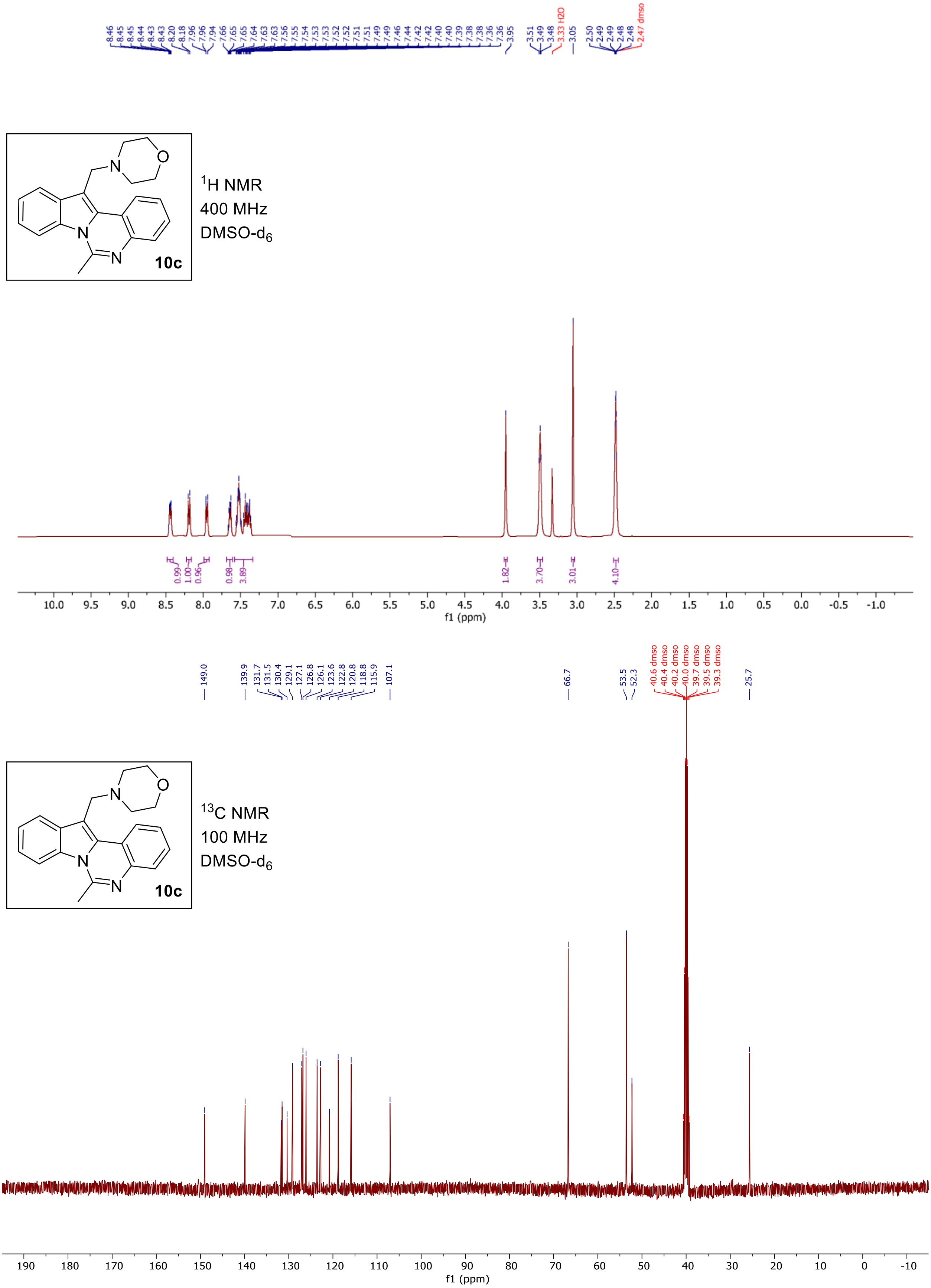

Figure S37. ^{1}H and ^{13}C NMR spectra of compound **10c**.

Compound Spectrum List Report

Analysis Info

Analysis Name	D:\Data\AST\KDV-45().d	Acquisition Date	1/19/2025
Method	tune_norm.m	Operator	Mitrokhov
Sample Name	Tune wide	Instrument / Ser#	micrOTOF-Q II 10225
Comment			

Acquisition Parameter

Source Type	ESI	Ion Polarity	Positive	Set Nebulizer	0.4 Bar
Focus	Not active	Set Capillary	4500 V	Set Dry Heater	180 °C
Scan Begin	50 m/z	Set End Plate Offset	-500 V	Set Dry Gas	4.0 l/min
Scan End	3000 m/z	Set Collision Cell RF	550.0 Vpp	Set Divert Valve	Source

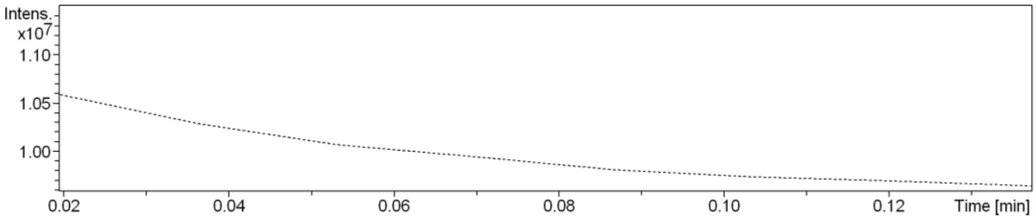

#	RT [min]	Area	Int. Type	Intens.	S/N	Chromatogram	Max. m/z
n.a.	0.1	n.a.	Average spectrum	n.a.	n.a.	n.a.	332.1748

+MS, 0.0-0.1min #(2-7)

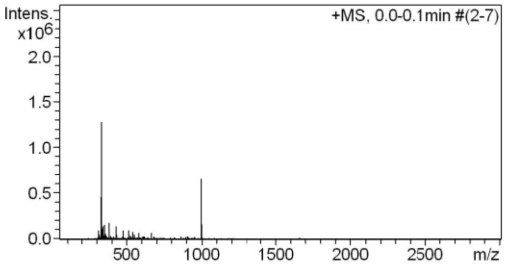

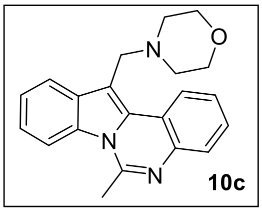

#	m/z	Res.	S/N	I	I %
1	330.1603	5112	246424.1	451778	35.5
2	332.1748	3834	693610.7	1271620	100.0
3	333.1800	4984	405887.6	744127	58.5
4	341.3039	5470	71185.0	130506	10.3
5	353.2660	5397	80656.5	147870	11.6
6	381.2970	5432	91733.2	168178	13.2
7	431.2425	5568	72098.7	132181	10.4
8	994.6232	6345	357164.6	654802	51.5
9	995.6252	6532	253116.3	464047	36.5
10	996.6272	6549	87736.6	160851	12.6

Bruker Compass DataAnalysis 4.0 printed: 1/19/2025 3:33:33 PM Page 1 of 1

Figure S38. Copy of HRMS (ESI) spectra of compound **10c**.

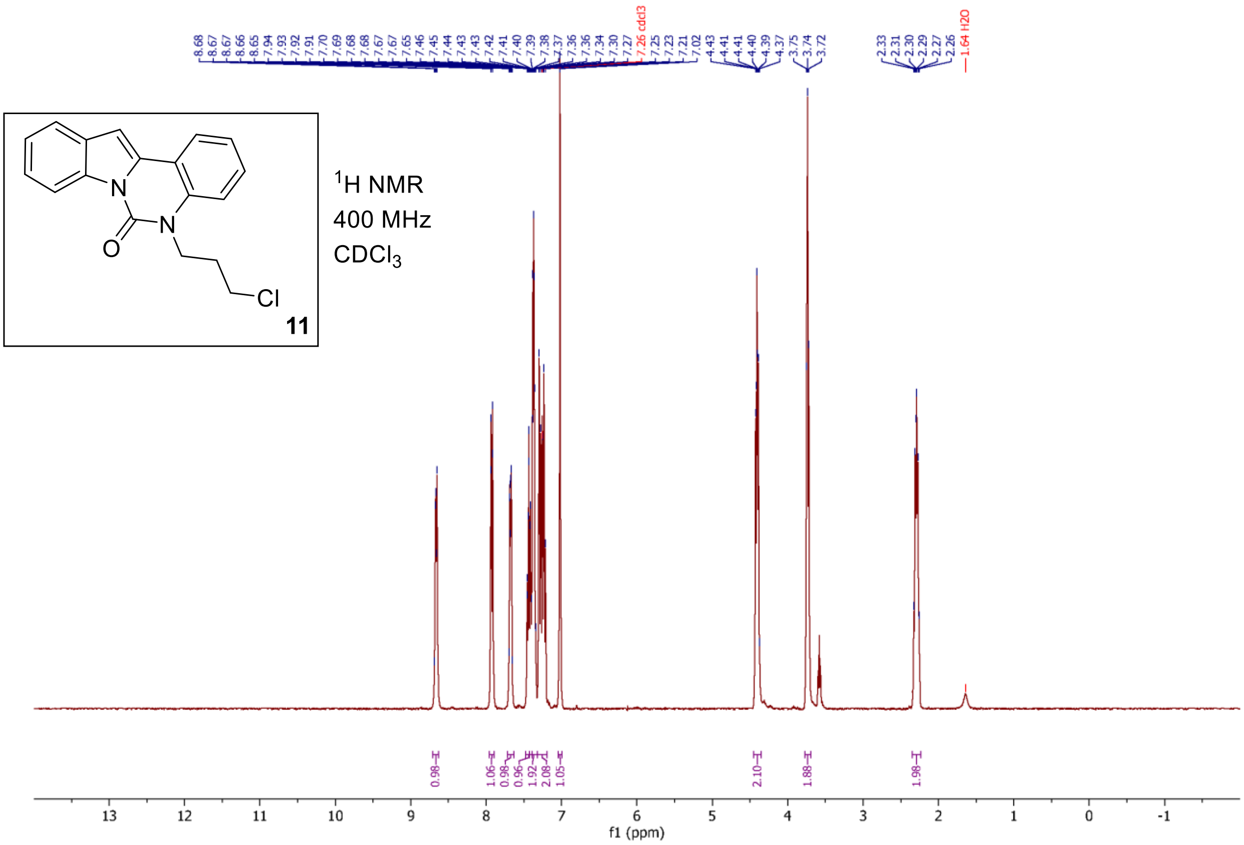

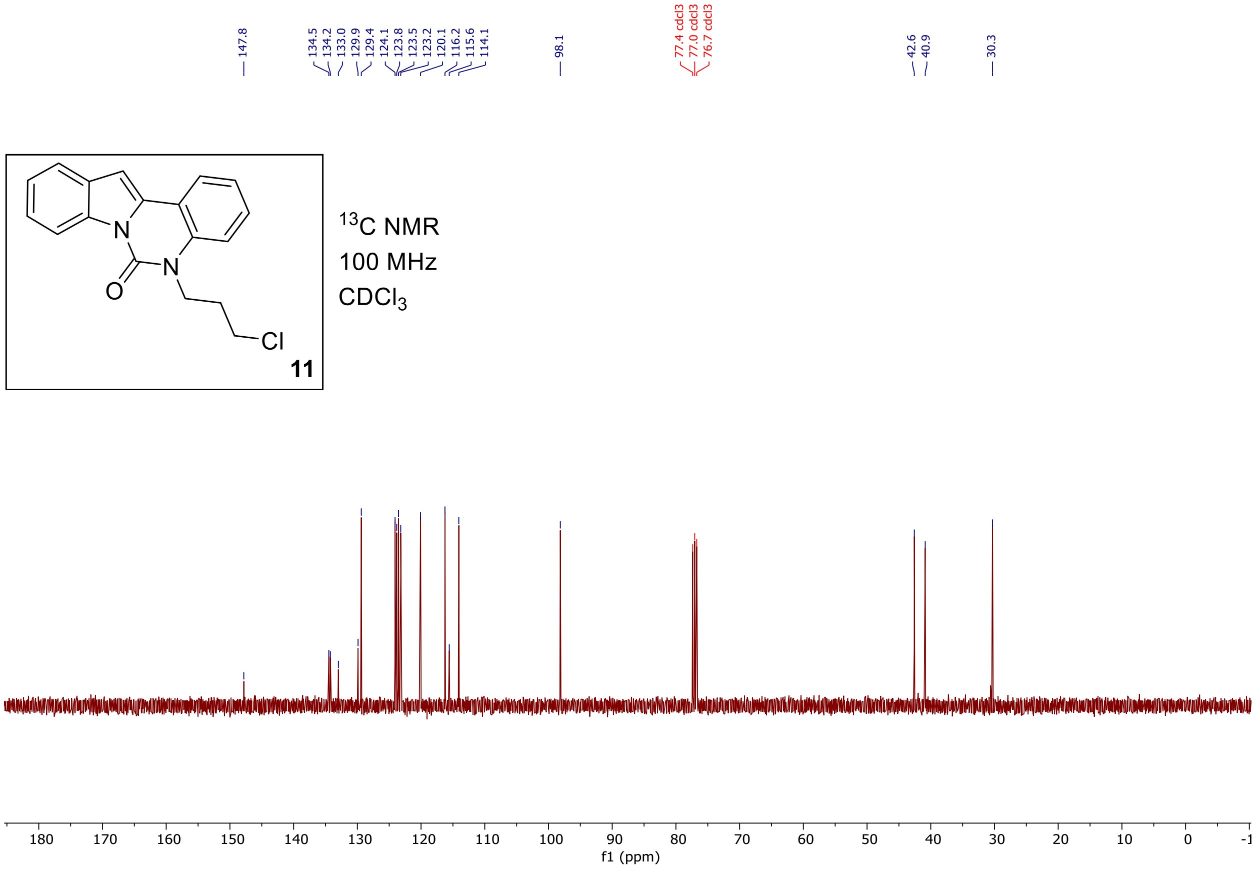

Figure S39. ^{1}H and ^{13}C NMR spectra of compound **11**.

Compound Spectrum List Report

Analysis Info

Analysis Name	D:\Data\AST\VAL-142().d	Acquisition Date	6/29/2025
Method	tune_norm.m	Operator	Mitrokhov
Sample Name	Tune wide	Instrument / Ser#	micrOTOF-Q II 10225
Comment			

Acquisition Parameter

Source Type	ESI	Ion Polarity	Positive	Set Nebulizer	0.4 Bar
Focus	Not active	Set Capillary	4500 V	Set Dry Heater	180 °C
Scan Begin	50 m/z	Set End Plate Offset	-500 V	Set Dry Gas	4.0 l/min
Scan End	3000 m/z	Set Collision Cell RF	550.0 Vpp	Set Divert Valve	Source

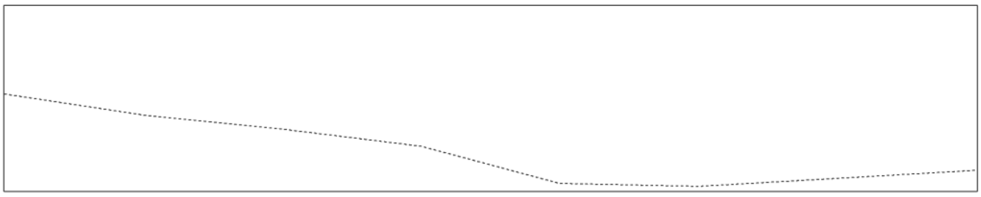

#	RT [min]	Area	Int. Type	Intens.	S/N	Chromatogram	Max. m/z
n.a.	0.1	n.a.	Average spectrum	n.a.	n.a.	n.a.	311.0955

+MS, 0.0-0.1min #(2-6)

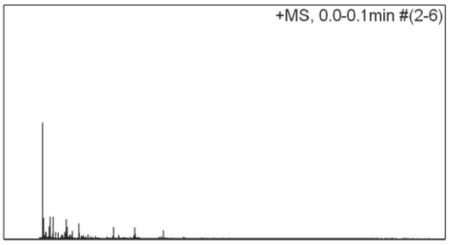

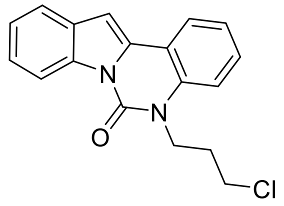

11

#	m/z	Res.	S/N	I	I %
1	311.0955	5250	124893.2	274765	100.0
2	312.1040	5394	25271.4	55597	20.2
3	313.0984	5510	46011.9	101226	36.8
4	319.0745	5789	21882.3	48141	17.5
5	357.0491	5506	12727.7	28001	10.2
6	361.2283	5591	22926.4	50438	18.4
7	383.2122	5590	23325.5	51316	18.7
8	471.1119	5493	20567.5	45248	16.5
9	477.8143	6243	12427.9	27341	10.0
10	555.1395	5818	16022.9	35250	12.8

Bruker Compass DataAnalysis 4.0 printed: 6/29/2025 1:29:20 PM Page 1 of 1

Figure S40. Copy of HRMS (ESI) spectra of compound **11**.

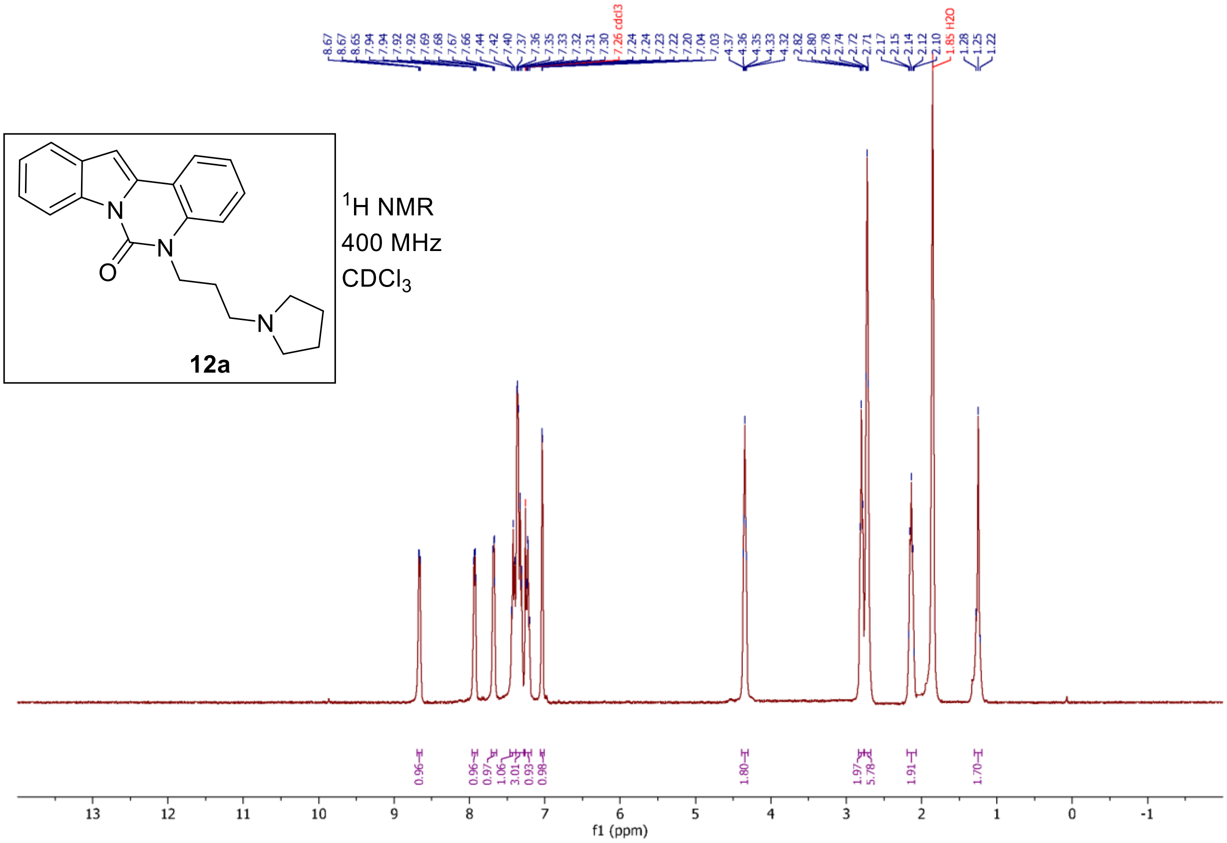

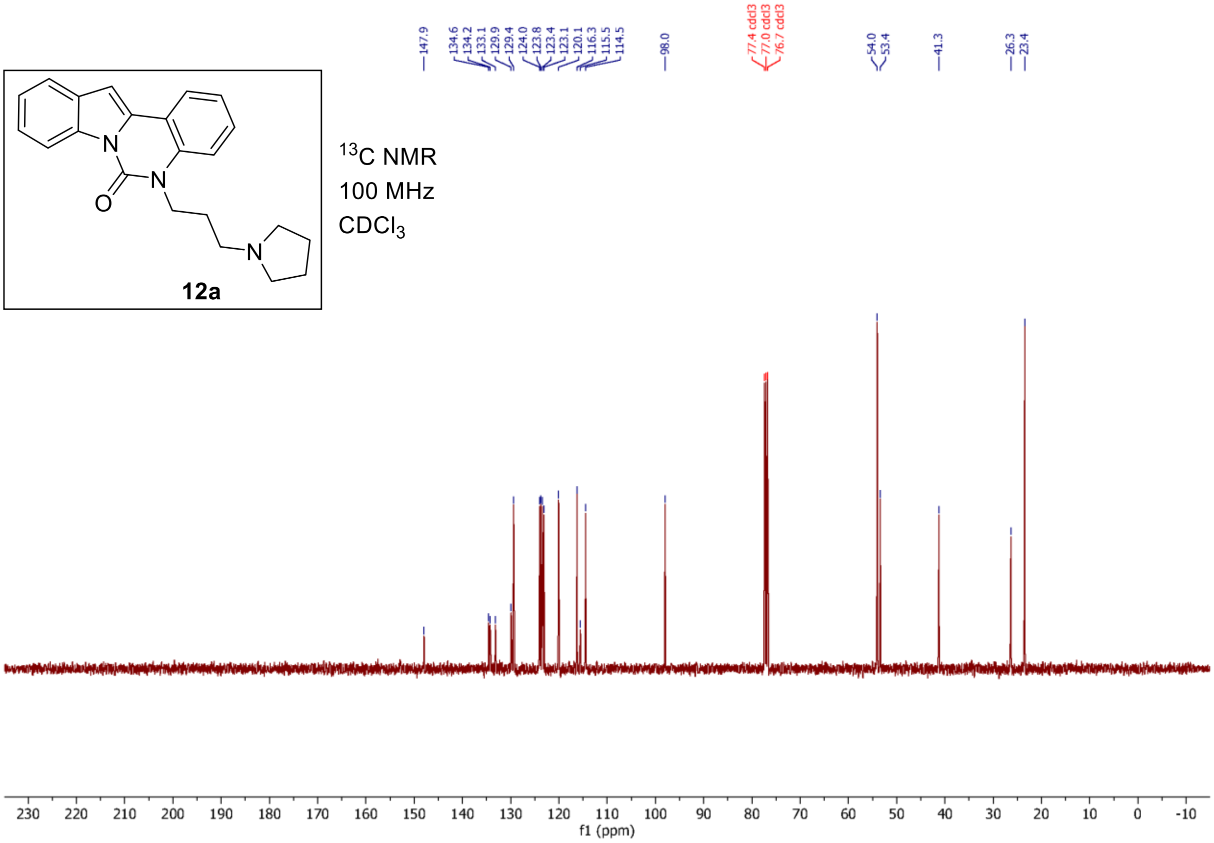

Figure S41. ^{1}H and ^{13}C NMR spectra of compound **12a**.

Compound Spectrum List Report

Analysis Info

Analysis Name	D:\Data\Litvinova\VAL-146.d	Acquisition Date	6/28/2025
Method	tune_norm.m	Operator	Mitrokhov
Sample Name	Tune wide	Instrument / Ser#	micrOTOF-Q II 10225
Comment			

Acquisition Parameter

Source Type	ESI	Ion Polarity	Positive	Set Nebulizer	0.4 Bar
Focus	Active	Set Capillary	4500 V	Set Dry Heater	180 °C
Scan Begin	50 m/z	Set End Plate Offset	-500 V	Set Dry Gas	4.0 l/min
Scan End	3000 m/z	Set Collision Cell RF	550.0 Vpp	Set Divert Valve	Source

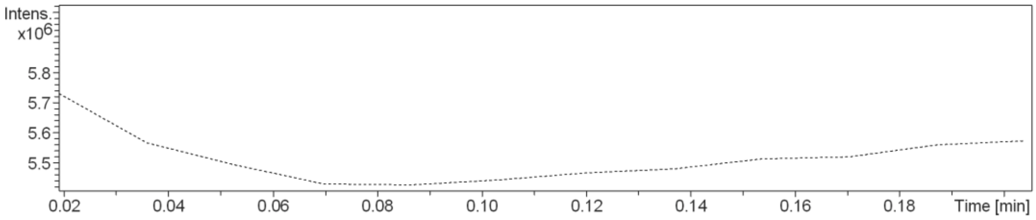

#	RT [min]	Area	Int. Type	Intens.	S/N	Chromatogram	Max. m/z
n.a.	0.1	n.a.	Average spectrum	n.a.	n.a.	n.a.	346.1925

+MS, 0.1-0.2min #(3-10)

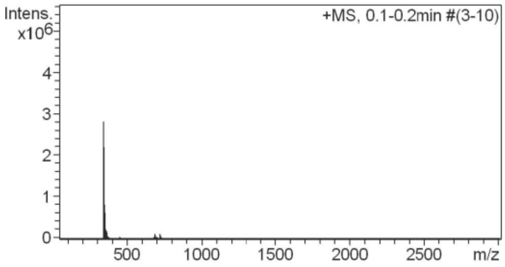

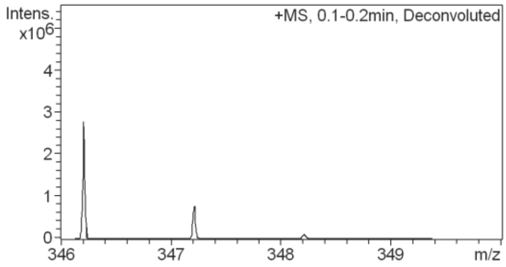

#	m/z	Res.	S/N	I	I %
1	344.1878	10152	415.1	52987	1.9
2	346.1925	19625	22135.5	2817008	100.0
3	347.2109	14022	6340.3	805829	28.6
4	348.2099	10120	694.3	88151	3.1
5	362.2000	11023	1317.9	164192	5.8
6	363.2025	9808	308.2	38359	1.4
7	685.3503	12603	722.2	93417	3.3
8	686.3535	11497	346.2	44872	1.6
9	723.3893	12342	640.5	88550	3.1
10	724.3917	11397	312.4	43281	1.5

#	m/z	Res.	S/N	I	I %
1	346.2091			2817008	100.0

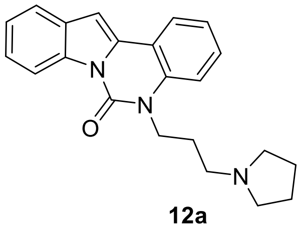

12a

Figure S42. Copy of HRMS (ESI) spectra of compound **12a**.

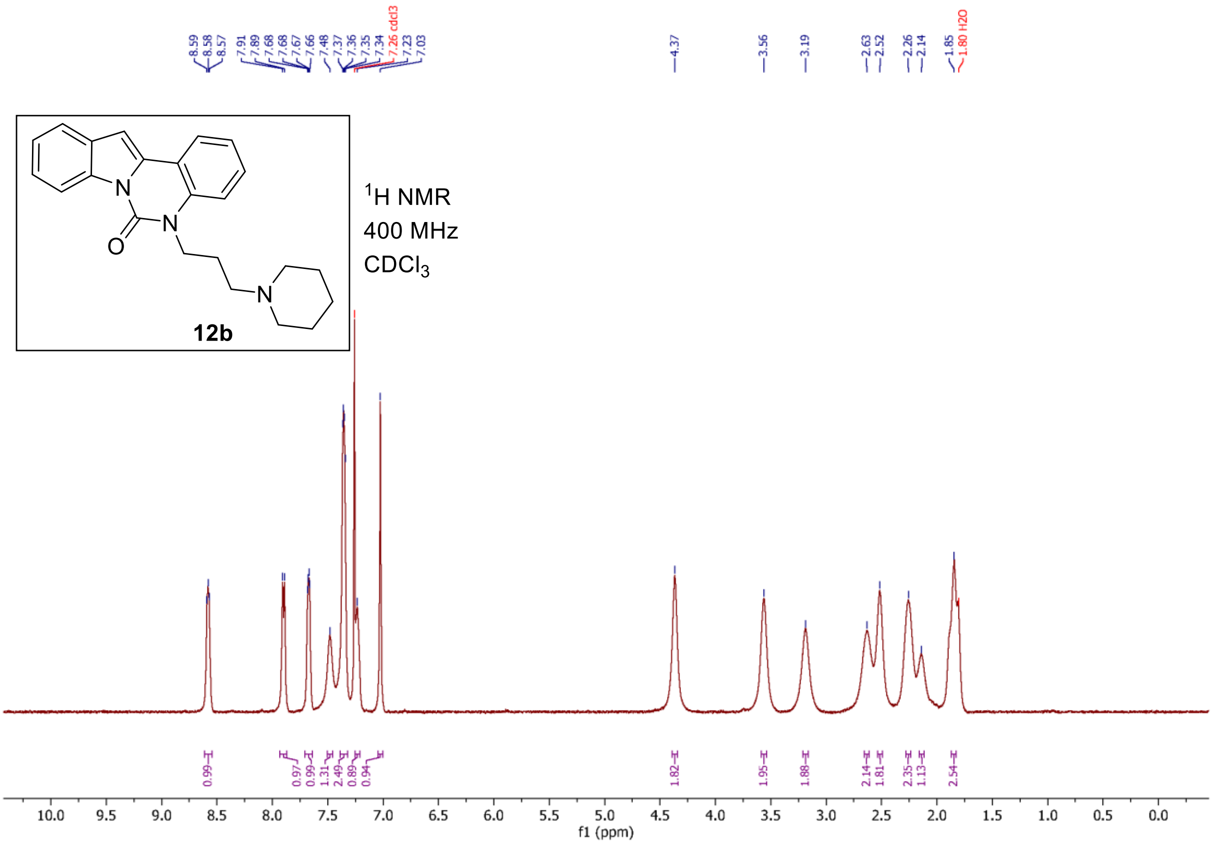

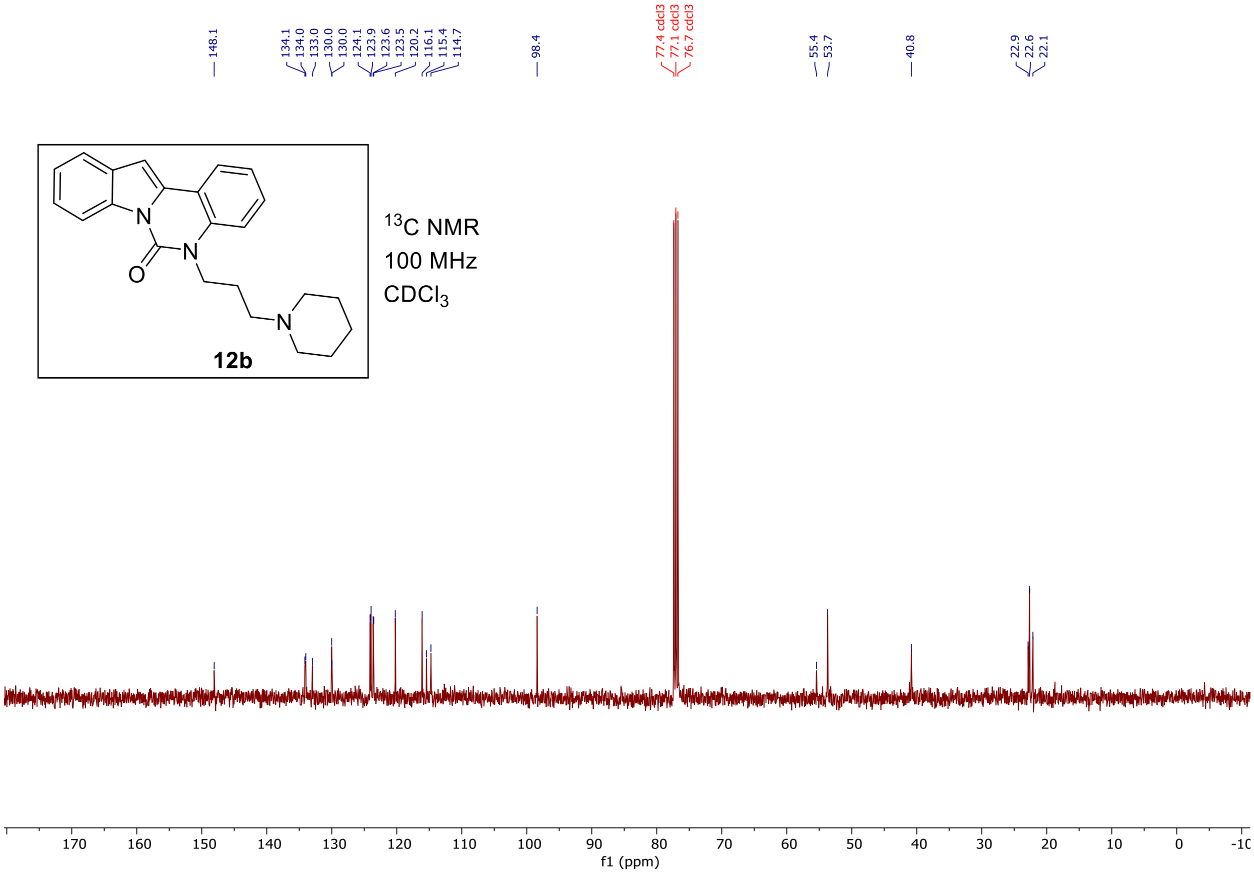

Figure S43. ^{1}H and ^{13}C NMR spectra of compound **12b**.

Compound Spectrum List Report

Analysis Info

Analysis Name	D:\Data\Litvinova\VAL-153().d	Acquisition Date	4/23/2023
Method	tune_norm.m	Operator	Mitrokhov
Sample Name	Tune wide	Instrument / Ser#	micrOTOF-Q II 10225
Comment			

Acquisition Parameter

Source Type	ESI	Ion Polarity	Positive	Set Nebulizer	0.4 Bar
Focus	Active	Set Capillary	4500 V	Set Dry Heater	180 °C
Scan Begin	50 m/z	Set End Plate Offset	-500 V	Set Dry Gas	4.0 l/min
Scan End	3000 m/z	Set Collision Cell RF	550.0 Vpp	Set Divert Valve	Source

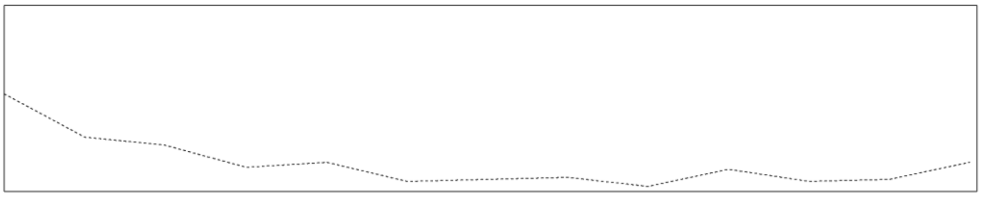

#	RT [min]	Area	Int. Type	Intens.	S/N	Chromatogram	Max. m/z
n.a.	0.1	n.a.	Average spectrum	n.a.	n.a.	n.a.	360.2081

+MS, 0.1-0.2min #(4-11)

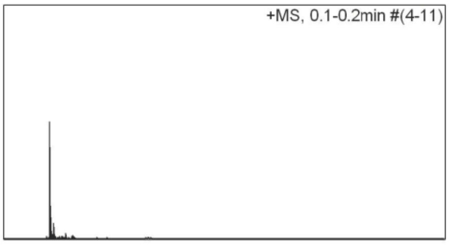

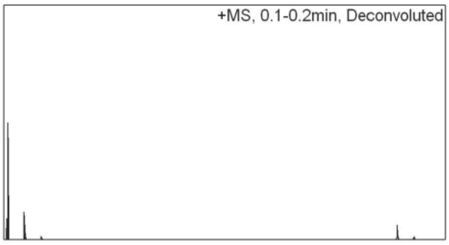

#	m/z	Res.	S/N	I	I %
1	360.2081	14165	5870.3	1307348	100.0
2	361.2088	11824	1374.6	307183	23.5
3	362.2087	10045	192.5	43217	3.3
4	374.1831	9435	150.2	35064	2.7
5	375.1878	9563	132.6	31066	2.4
6	376.1969	9421	186.0	43681	3.3
7	383.2008	11215	727.3	174299	13.3
8	384.2015	9706	143.4	34555	2.6
9	464.2303	10660	197.3	59156	4.5
10	515.7894	11083	86.7	29427	2.3

#	m/z	Res.	S/N	I	I %
1	360.2086			1307347	100.0
2	383.2003			174298	13.3

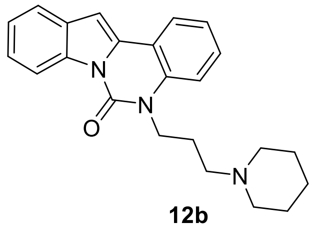

12b

Bruker Compass DataAnalysis 4.0 printed: 4/23/2023 8:04:17 PM Page 1 of 1

Figure S44. Copy of HRMS (ESI) spectra of compound **12b**.

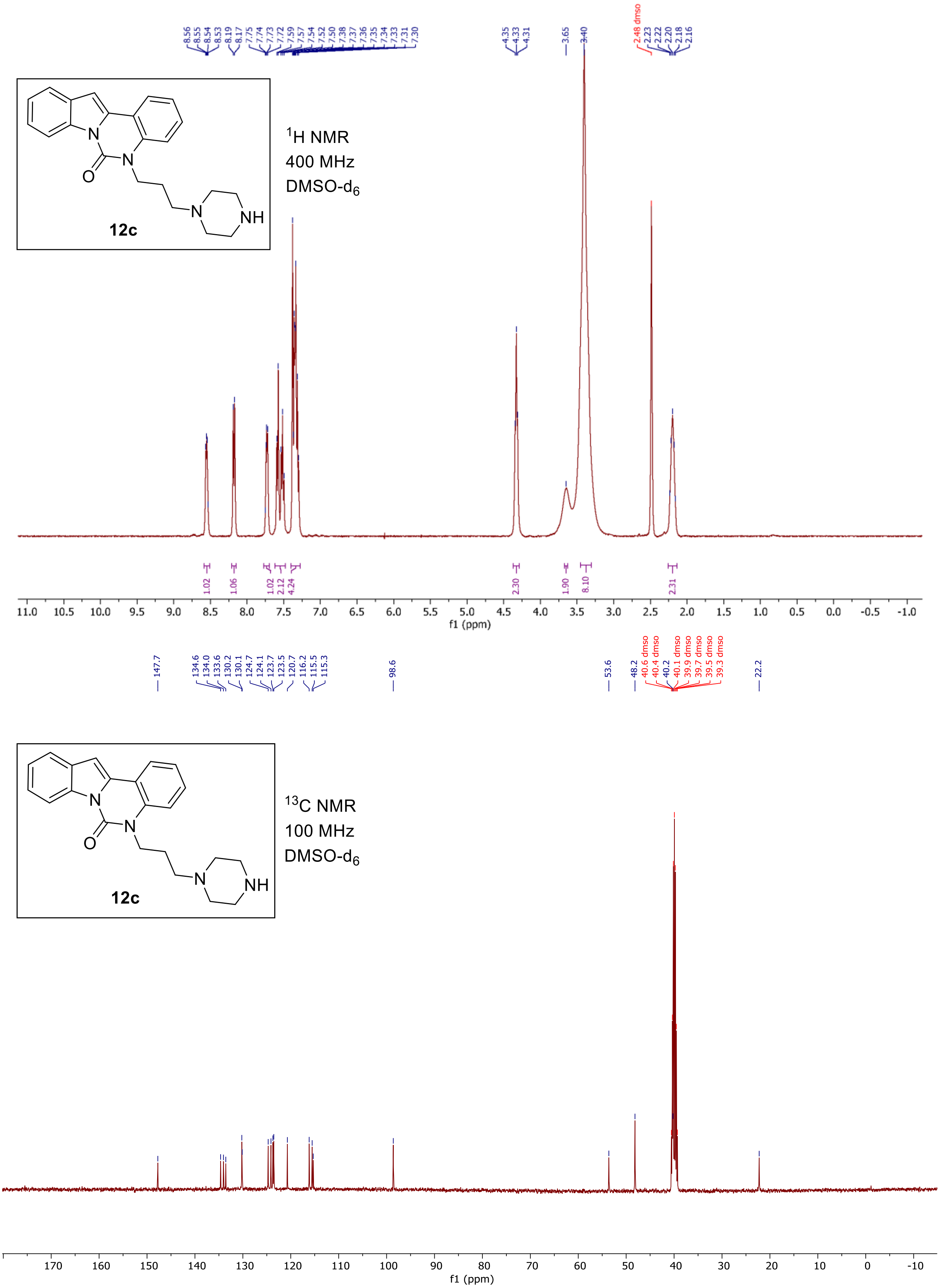

Figure S45. ^{1}H and ^{13}C NMR spectra of compound **12c**.

Compound Spectrum List Report

Analysis Info

Analysis Name	D:\Data\Litvinova\VAL-157.d	Acquisition Date	6/29/2025
Method	tune_norm.m	Operator	Mitrokhov
Sample Name	Tune wide	Instrument / Ser#	micrOTOF-Q II 10225
Comment			

Acquisition Parameter

Source Type	ESI	Ion Polarity	Positive	Set Nebulizer	0.4 Bar
Focus	Active	Set Capillary	4500 V	Set Dry Heater	180 °C
Scan Begin	50 m/z	Set End Plate Offset	-500 V	Set Dry Gas	4.0 l/min
Scan End	3000 m/z	Set Collision Cell RF	550.0 Vpp	Set Divert Valve	Source

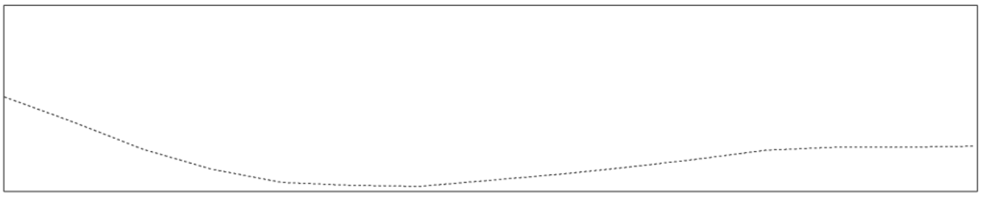

#	RT [min]	Area	Int. Type	Intens.	S/N	Chromatogram	Max. m/z
n.a.	0.1	n.a.	Average spectrum	n.a.	n.a.	n.a.	361.2013

+MS, 0.1-0.2min #(3-13)

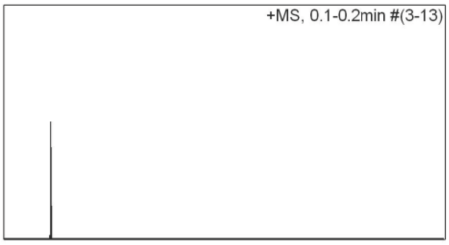

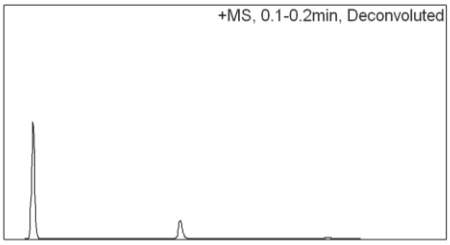

#	m/z	Res.	S/N	I	I %
1	341.2985	11666	114.6	2009	0.3
2	357.1664	10869	346.8	6195	1.0
3	358.1704	9970	88.8	1588	0.3
4	359.1808	11816	871.4	15599	2.5
5	360.1830	11636	182.5	3271	0.5
6	361.2013	19220	34114.9	612088	100.0
7	362.1996	12248	4986.4	89569	14.6
8	363.2028	10300	338.4	6087	1.0
9	375.1758	10555	146.8	2677	0.4
10	383.1891	7747	97.0	1785	0.3

#	m/z	Res.	S/N	I	I %
1	361.1991			612088	100.0

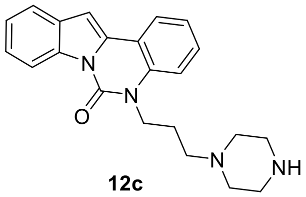

12c

Bruker Compass DataAnalysis 4.0 printed: 6/29/2025 7:36:54 PM Page 1 of 1

Figure S46. Copy of HRMS (ESI) spectra of compound **12c**.

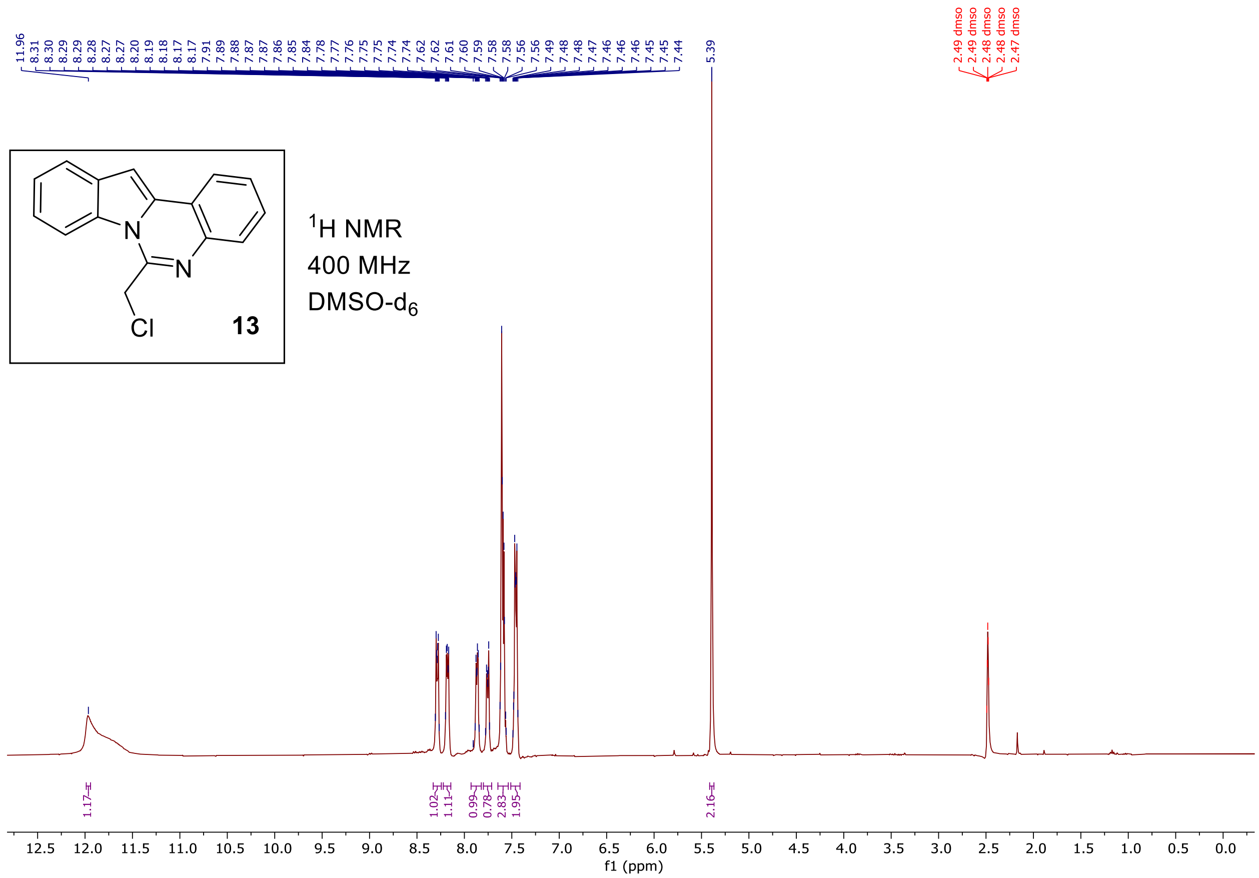

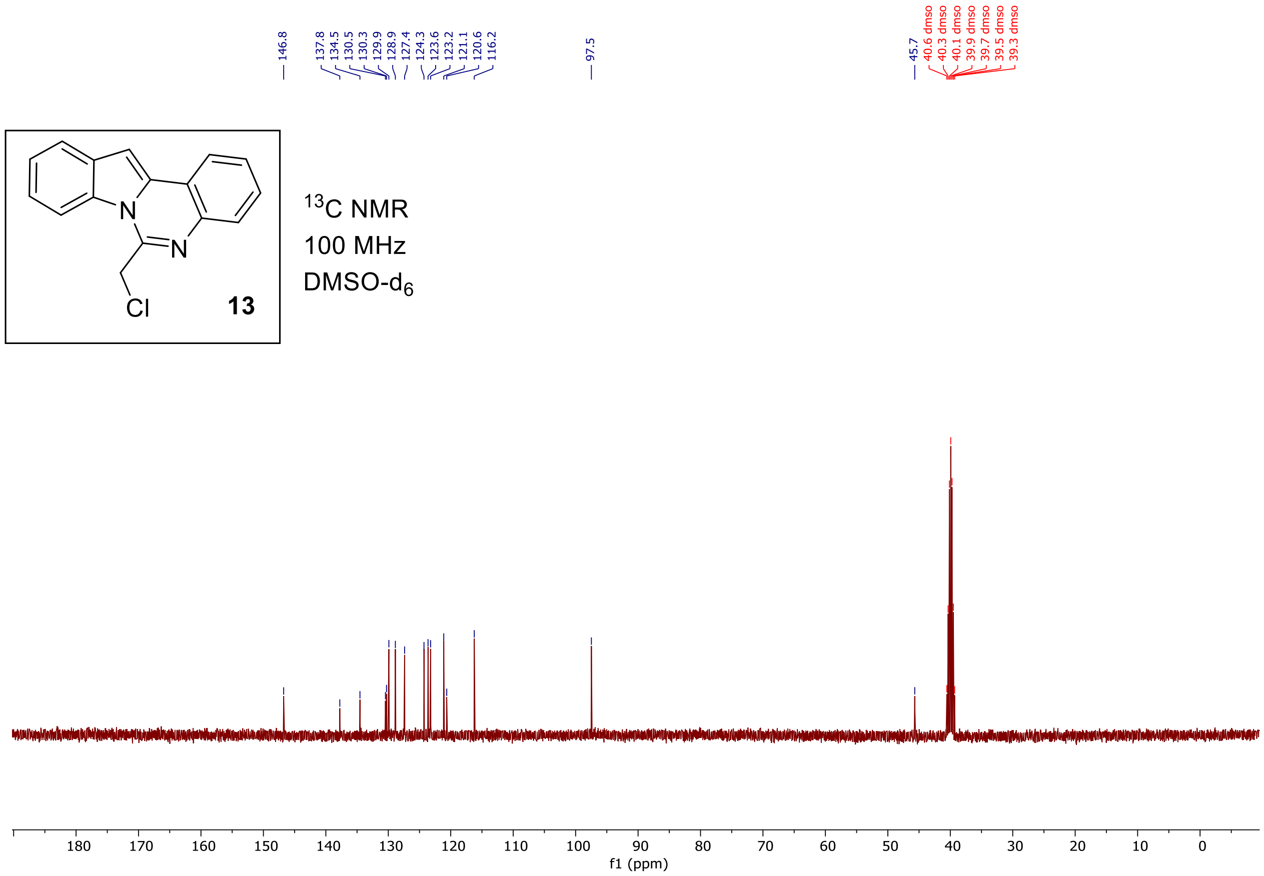

Figure S47. Copy of HRMS (ESI) spectra of compound **12c**.

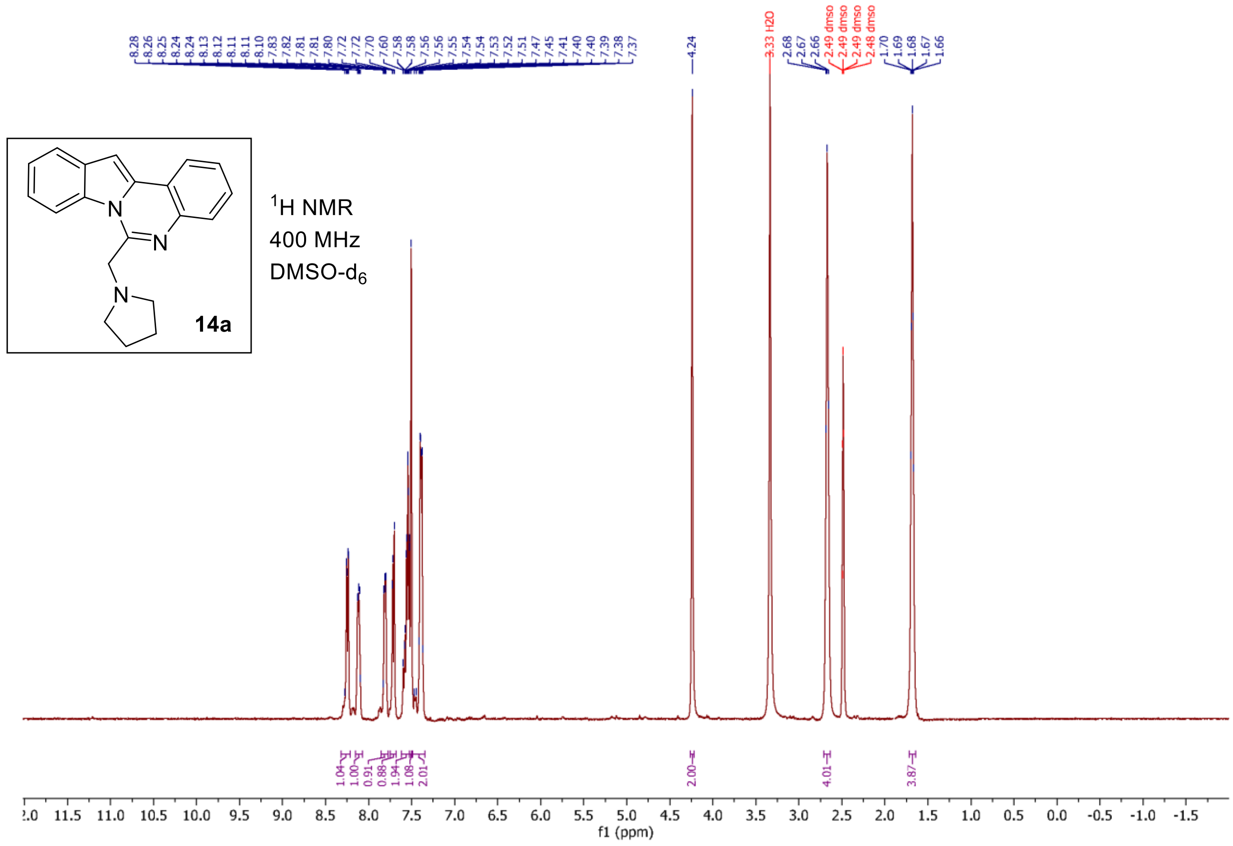

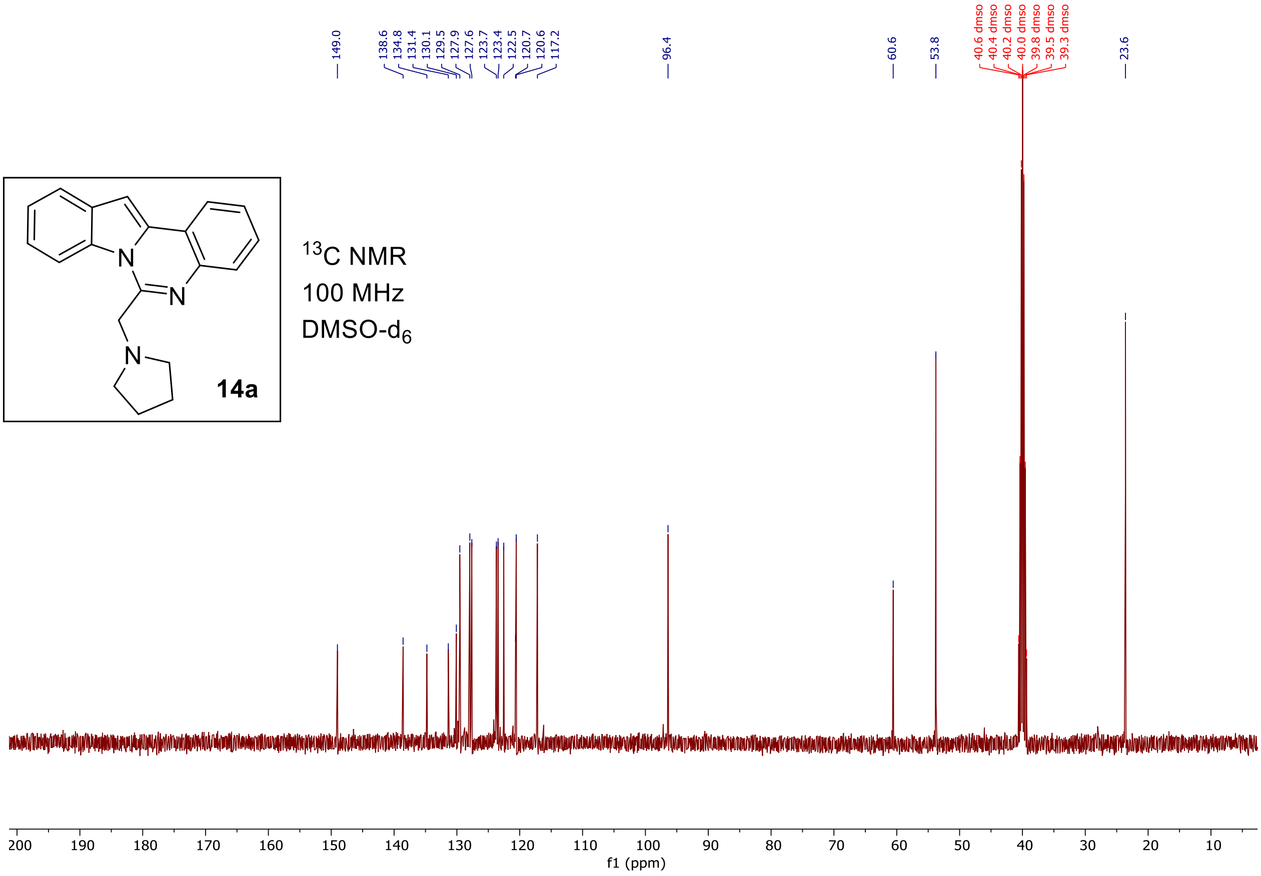

Figure S48. ^{1}H and ^{13}C NMR spectra of compound **14a**.

Compound Spectrum List Report

Analysis Info

Analysis Name	D:\Data\AST\KDV-68.d	Acquisition Date	6/27/2025
Method	tune_norm.m	Operator	Mitrokhov
Sample Name	Tune wide	Instrument / Ser#	micrOTOF-Q II 10225
Comment			

Acquisition Parameter

Source Type	ESI	Ion Polarity	Positive	Set Nebulizer	0.4 Bar
Focus	Not active	Set Capillary	4500 V	Set Dry Heater	180 °C
Scan Begin	50 m/z	Set End Plate Offset	-500 V	Set Dry Gas	4.0 l/min
Scan End	3000 m/z	Set Collision Cell RF	550.0 Vpp	Set Divert Valve	Source

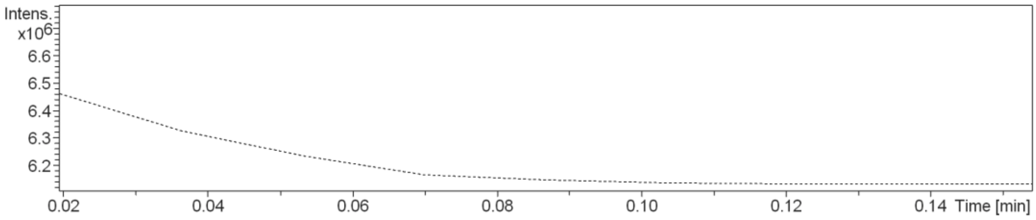

#	RT [min]	Area	Int. Type	Intens.	S/N	Chromatogram	Max. m/z
n.a.	0.1	n.a.	Average spectrum	n.a.	n.a.	n.a.	302.1661

+MS, 0.0-0.1min #(2-7)

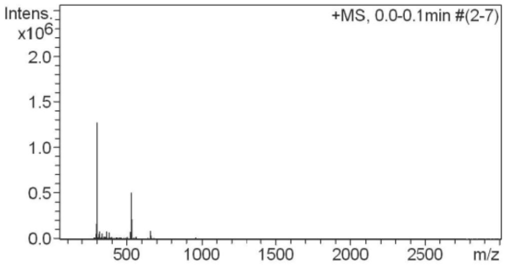

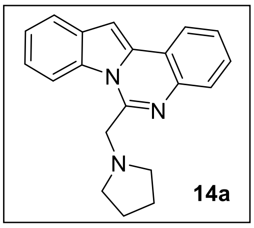

#	m/z	Res.	S/N	I	I %
1	298.1386	5305	40042.5	73411	5.8
2	300.1544	5275	87086.4	159658	12.5
3	302.1661	3304	695297.9	1274713	100.0
4	303.1749	4553	519779.4	952929	74.8
5	304.1766	5572	73691.5	135101	10.6
6	318.2415	5296	41333.8	75779	5.9
7	367.1970	5537	41340.5	75791	5.9
8	532.2594	5719	272699.1	499948	39.2
9	533.2616	5887	114669.4	210227	16.5
10	660.2596	6306	44630.5	81823	6.4

Bruker Compass DataAnalysis 4.0 printed: 6/27/2025 6:51:53 PM Page 1 of 1

Figure S49. Copy of HRMS (ESI) spectra of compound **14a**.

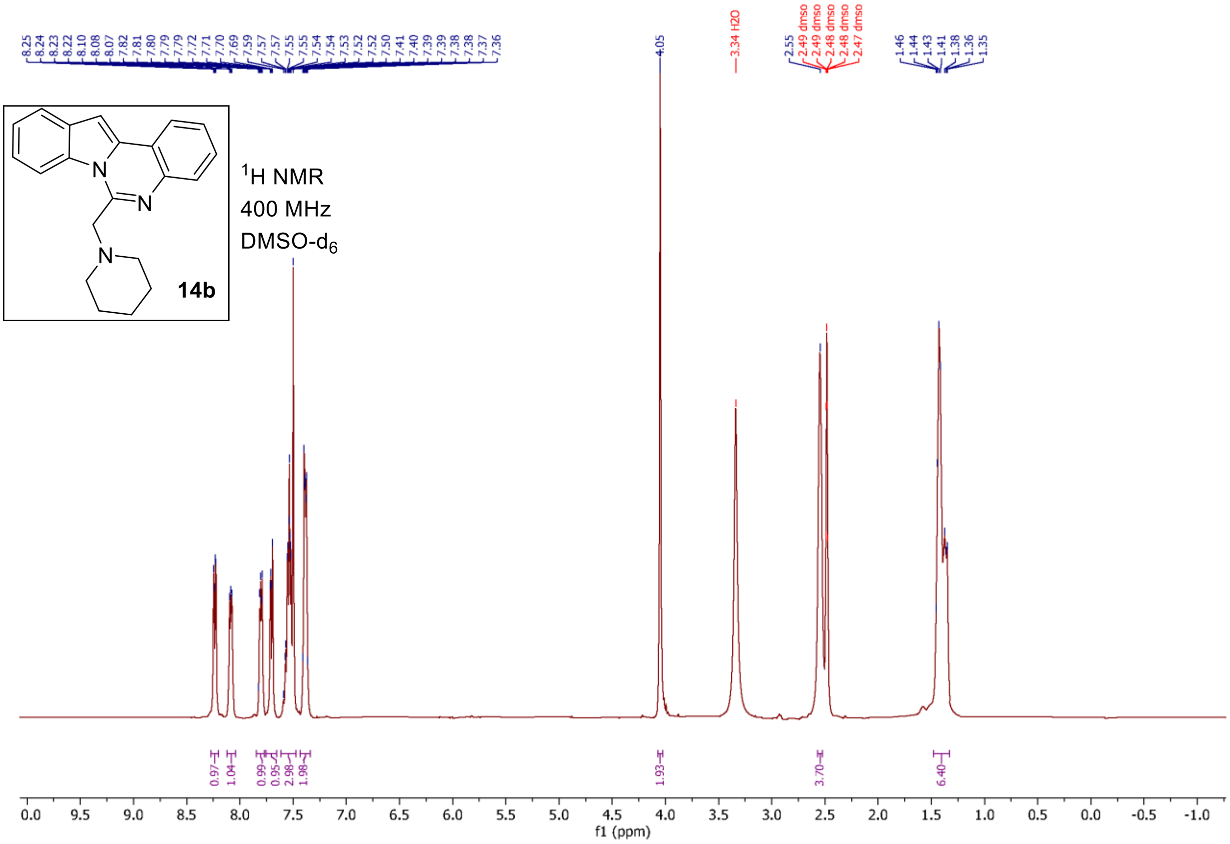

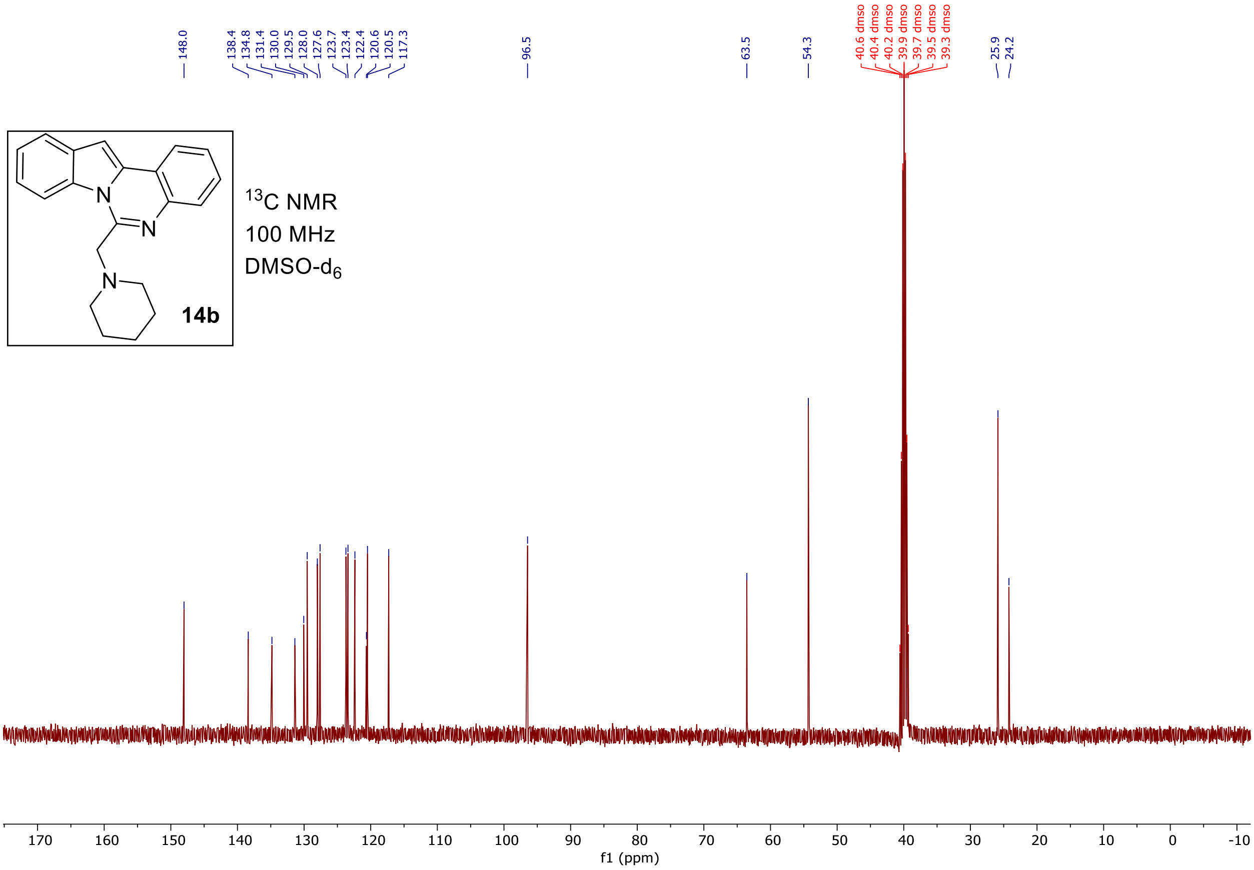

Figure S50. ^{1}H and ^{13}C NMR spectra of compound **14b**.

Compound Spectrum List Report

Analysis Info

Analysis Name	D:\Data\AST\KDV-51.d	Acquisition Date	11/22/2024
Method	tune_low_norm.m	Operator	Mitrokhov
Sample Name	Tune wide	Instrument / Ser#	micrOTOF-Q II 10225
Comment			

Acquisition Parameter

Source Type	ESI	Ion Polarity	Positive	Set Nebulizer	0.4 Bar
Focus	Active	Set Capillary	4500 V	Set Dry Heater	180 °C
Scan Begin	50 m/z	Set End Plate Offset	-500 V	Set Dry Gas	4.0 l/min
Scan End	3000 m/z	Set Collision Cell RF	150.0 Vpp	Set Divert Valve	Source

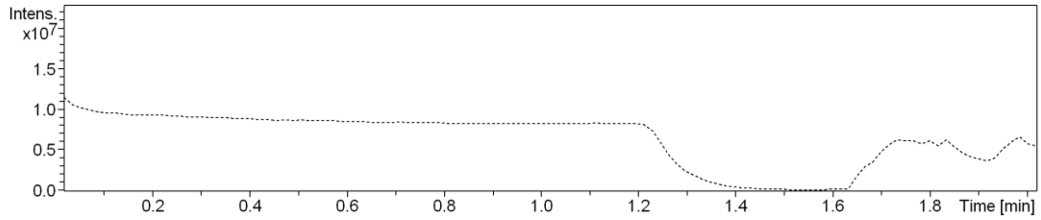

#	RT [min]	Area	Max. m/z
n.a.	0.1	n.a.	316.1816

+MS, 0.1-0.2min #(3-13)

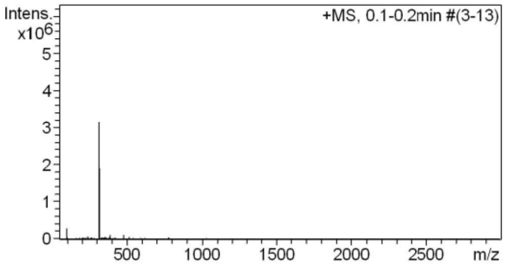

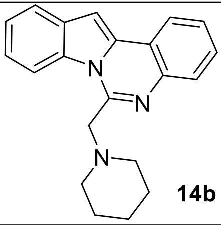

14b

#	m/z	I	I %
1	98.0988	275078	8.8
2	242.2875	52502	1.7
3	314.1680	305100	9.7
4	315.1699	79574	2.5
5	316.1816	3138921	100.0
6	317.1898	1876999	59.8
7	318.1880	279458	8.9
8	389.2641	98243	3.1
9	477.8087	89475	2.9
10	478.3101	49494	1.6

Figure S51. Copy of HRMS (ESI) spectra of compound **14b**.

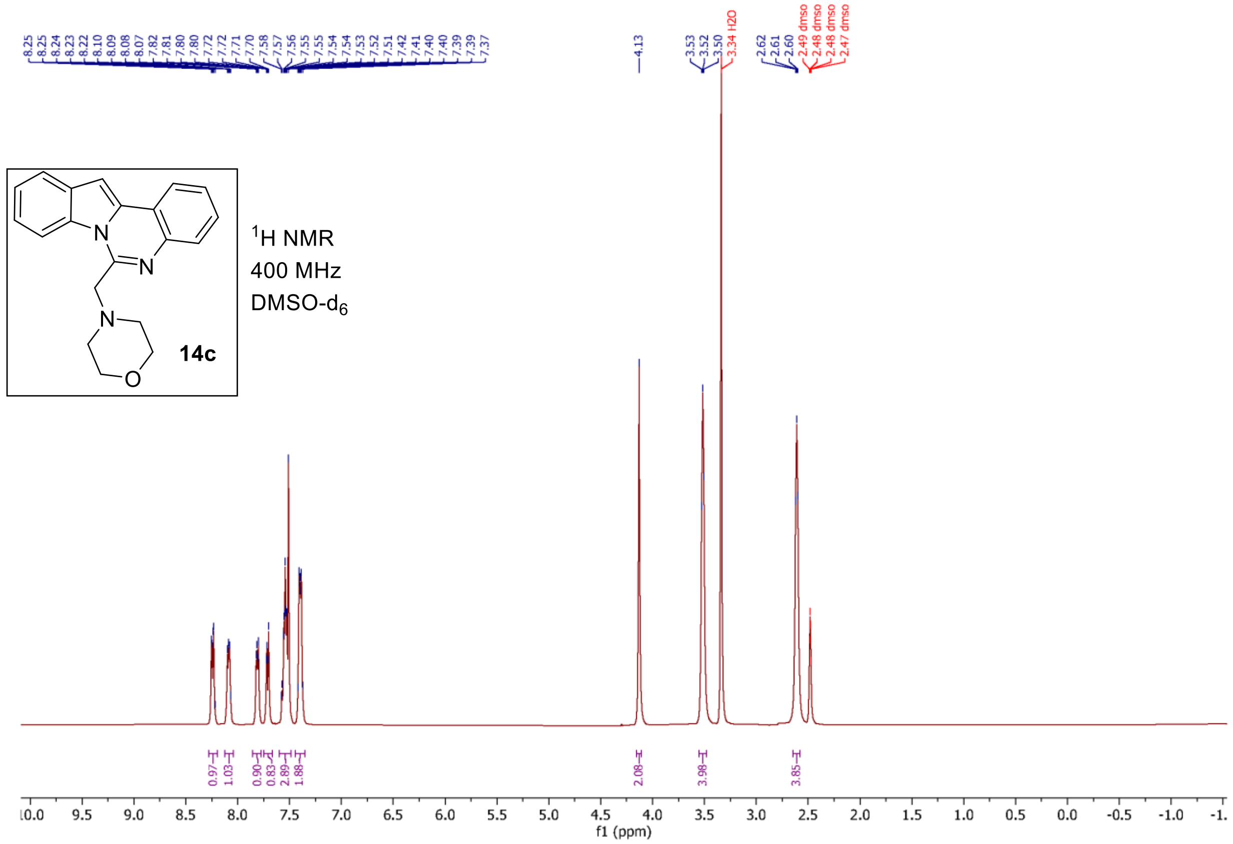

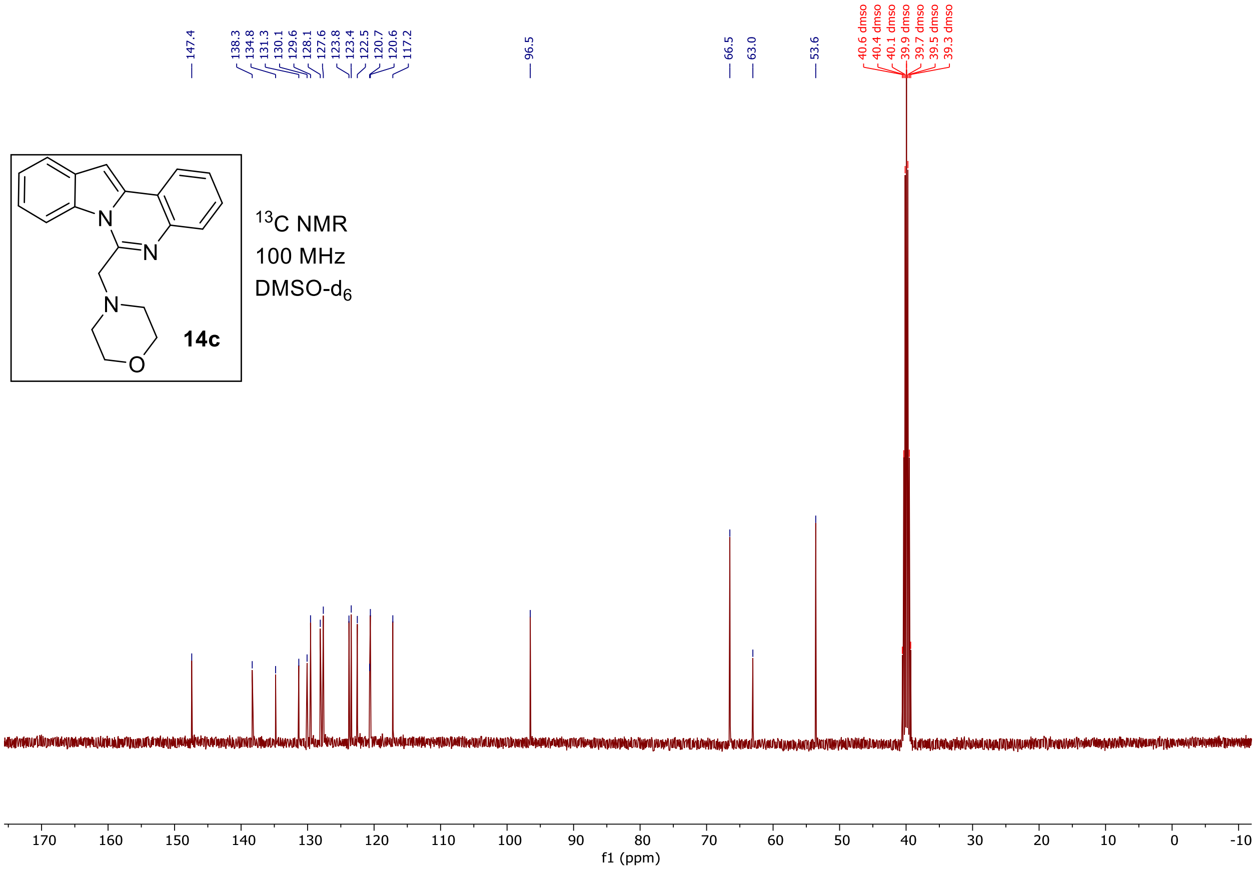

Figure S52. ^{1}H and ^{13}C NMR spectra of compound **14c**.

Compound Spectrum List Report

Analysis Info

Analysis Name	D:\Data\AST\KDV-53().d	Acquisition Date	6/28/2025
Method	tune_low_norm.m	Operator	Mitrokhov
Sample Name	Tune wide	Instrument / Ser#	micrOTOF-Q II 10225
Comment			

Acquisition Parameter

Source Type	ESI	Ion Polarity	Negative	Set Nebulizer	0.4 Bar
Focus	Active	Set Capillary	3500 V	Set Dry Heater	180 °C
Scan Begin	50 m/z	Set End Plate Offset	-500 V	Set Dry Gas	4.0 l/min
Scan End	3000 m/z	Set Collision Cell RF	150.0 Vpp	Set Divert Valve	Source

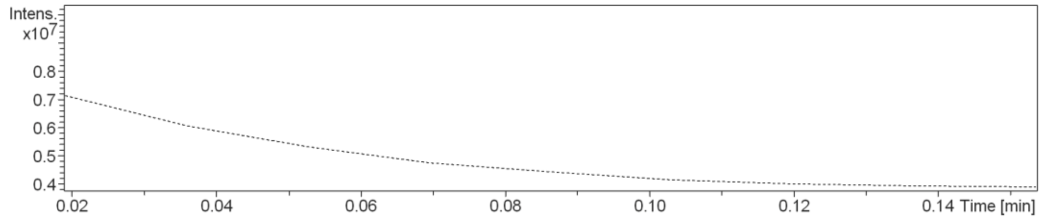

#	RT [min]	Area	Max. m/z
n.a.	0.1	n.a.	316.1439

-MS, 0.1-0.1min #(3-8)

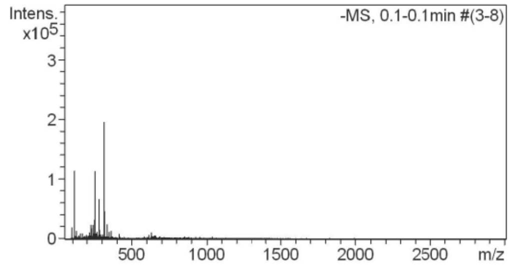

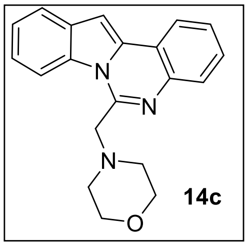

14c

#	m/z	I	I %
1	112.9812	112917	57.9
2	227.1952	22360	11.5
3	241.2097	22377	11.5
4	253.2088	31152	16.0
5	255.2266	112395	57.6
6	281.2408	24633	12.6
7	283.2567	65619	33.7
8	316.1439	194993	100.0
9	317.1405	45266	23.2
10	334.1486	23280	11.9

Bruker Compass DataAnalysis 4.0 printed: 6/28/2025 8:02:36 PM Page 1 of 1

Figure S53. Copy of HRMS (ESI) spectra of compound **14c**.

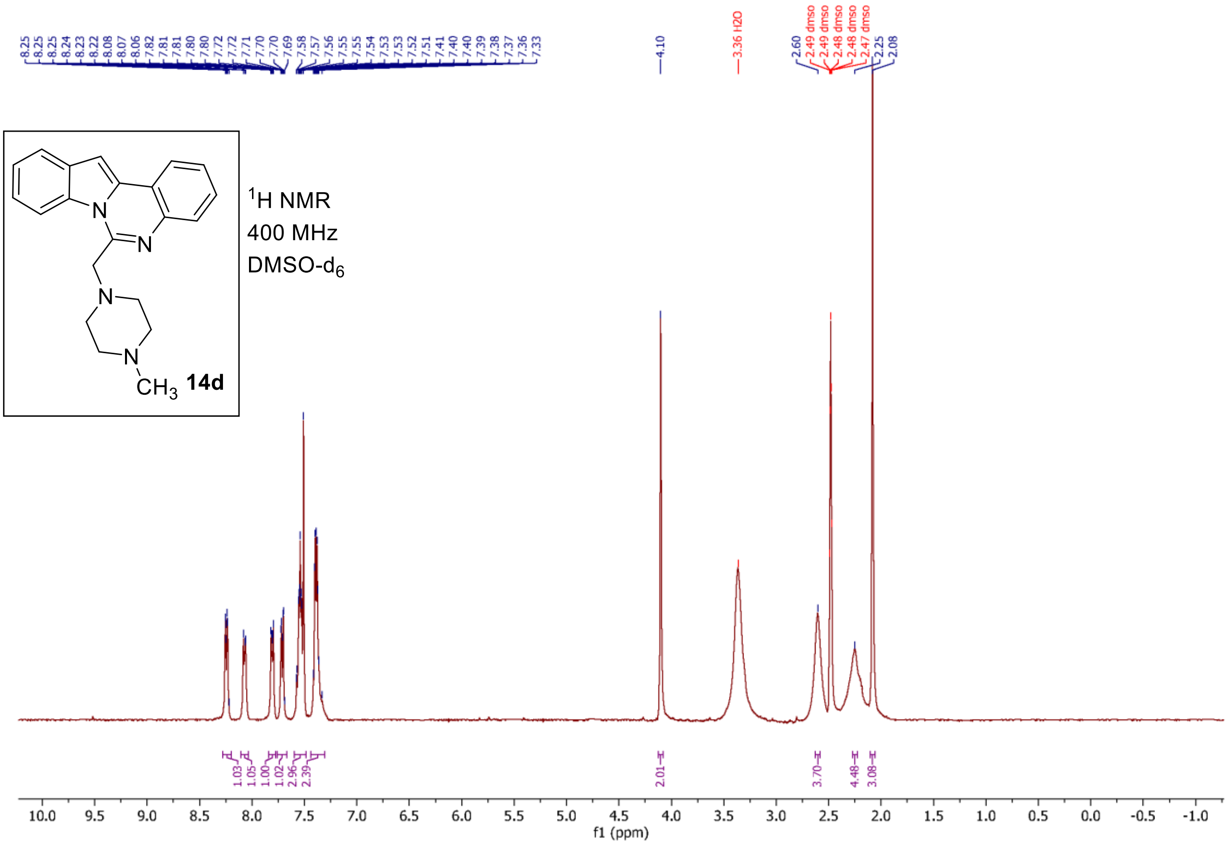

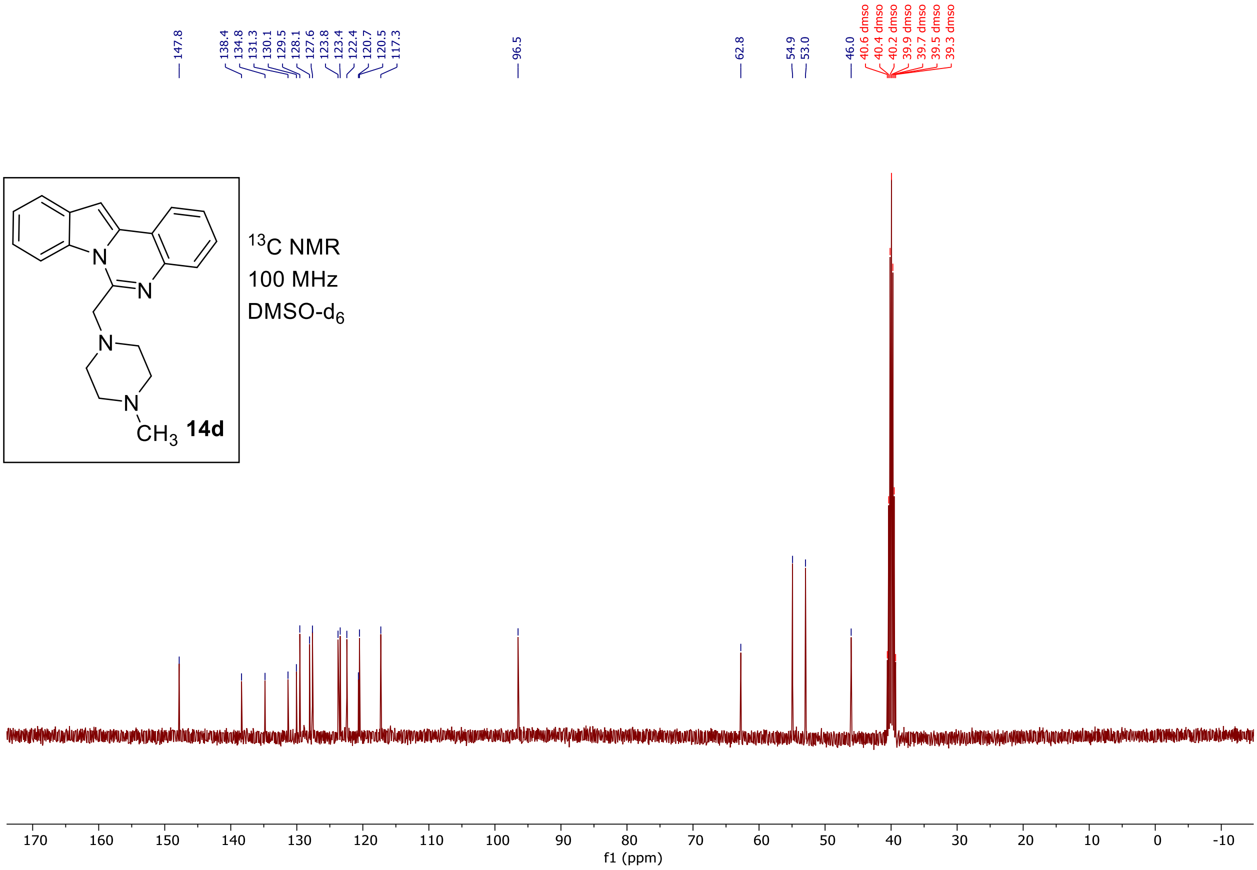

Figure S54. ^{1}H and ^{13}C NMR spectra of compound **14d**.

Compound Spectrum List Report

Analysis Info

Analysis Name	D:\Data\AST\KDV-52.d	Acquisition Date	6/29/2025
Method	tune_norm.m	Operator	Mitrokhov
Sample Name	Tune wide	Instrument / Ser#	micrOTOF-Q II 10225
Comment			

Acquisition Parameter

Source Type	ESI	Ion Polarity	Positive	Set Nebulizer	0.4 Bar
Focus	Not active	Set Capillary	4500 V	Set Dry Heater	180 °C
Scan Begin	50 m/z	Set End Plate Offset	-500 V	Set Dry Gas	4.0 l/min
Scan End	3000 m/z	Set Collision Cell RF	550.0 Vpp	Set Divert Valve	Source

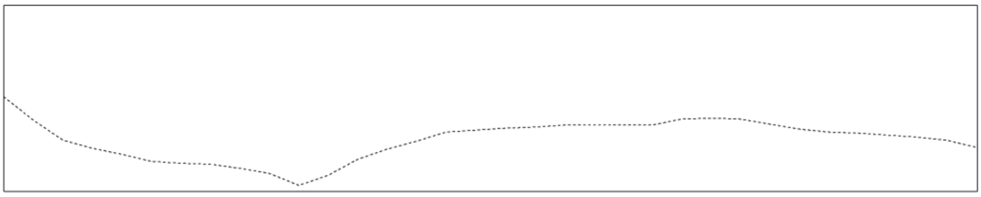

#	RT [min]	Area	Int. Type	Intens.	S/N	Chromatogram	Max. m/z
n.a.	0.3	n.a.	Average spectrum	n.a.	n.a.	n.a.	331.1922

+MS, 0.1-0.5min #(5-28)

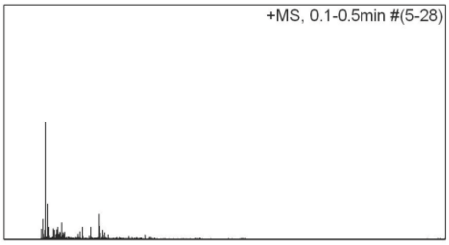

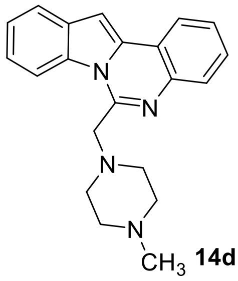

14d

#	m/z	Res.	S/N	I	I %
1	317.1741	5399	465509.8	213359	16.7
2	331.1922	2740	2781818.0	1275000	100.0
3	332.1941	4292	2708072.8	1241200	97.3
4	333.1945	5746	576094.9	264044	20.7
5	347.1845	5554	823752.3	377553	29.6
6	413.2401	5684	279561.5	128132	10.0
7	439.2215	5810	385652.8	176758	13.9
8	577.2676	6252	278484.6	127639	10.0
9	689.3326	6394	586869.3	268982	21.1
10	690.3352	6592	293402.7	134476	10.5

Bruker Compass DataAnalysis 4.0 printed: 6/29/2025 8:54:11 PM Page 1 of 1

Figure S55. Copy of HRMS (ESI) spectra of compound **14d**.
